# Identification of optimal fluorescent probes for G-quadruplex nucleic acids through systematic exploration of mono- and distyryl dye libraries

**DOI:** 10.3762/bjoc.15.183

**Published:** 2019-08-06

**Authors:** Xiao Xie, Michela Zuffo, Marie-Paule Teulade-Fichou, Anton Granzhan

**Affiliations:** 1 CNRS UMR9187, INSERM U1196, Institut Curie, Université Paris Sud, Université Paris Saclay, Bât. 110, Centre universitaire Paris Sud, F-91405 Orsay, France

**Keywords:** fluorescent probes, G-quadruplex DNA, G-quadruplex RNA, nucleic acids, styryl dyes

## Abstract

A library of 52 distyryl and 9 mono-styryl cationic dyes was synthesized and investigated with respect to their optical properties, propensity to aggregation in aqueous medium, and capacity to serve as fluorescence “light-up” probes for G-quadruplex (G4) DNA and RNA structures. Among the 61 compounds, 57 dyes showed preferential enhancement of fluorescence intensity in the presence of one or another G4-DNA or RNA structure, while no dye displayed preferential response to double-stranded DNA or single-stranded RNA analytes employed at equivalent nucleotide concentration. Thus, preferential fluorimetric response towards G4 structures appears to be a common feature of mono- and distyryl dyes, including long-known mono-styryl dyes used as mitochondrial probes or protein stains. However, the magnitude of the G4-induced “light-up” effect varies drastically, as a function of both the molecular structure of the dyes and the nature or topology of G4 analytes. Although our results do not allow to formulate comprehensive structure–properties relationships, we identified several structural motifs, such as indole- or pyrrole-substituted distyryl dyes, as well as simple mono-stryryl dyes such as DASPMI [2-(4-(dimethylamino)styryl)-1-methylpyridinium iodide] or its 4-isomer, as optimal fluorescent light-up probes characterized by high fluorimetric response (*I*/*I*_0_ of up to 550-fold), excellent selectivity with respect to double-stranded DNA or single-stranded RNA controls, high quantum yield in the presence of G4 analytes (up to 0.32), large Stokes shift (up to 150 nm) and, in certain cases, structural selectivity with respect to one or another G4 folding topology. These dyes can be considered as promising G4-responsive sensors for in vitro or imaging applications. As a possible application, we implemented a simple two-dye fluorimetric assay allowing rapid topological classification of G4-DNA structures.

## Introduction

Development of fluorescent probes for G-quadruplex (G4) DNA and RNA is an active research area. In fact, these non-canonical nucleic acid structures appear to be biologically relevant, although a complete understanding of their roles is still missing [1–3]. At the same time, they represent versatile building blocks for artificial nano-architectures and nanodevices [4–5]. In this context, small-molecule fluorescent probes find applications for in vitro detection of G4 structures and their differentiation from other DNA or RNA forms [6–10], topological characterization of G4 structures [11–14], real-time detection of G4 formation [15], and implementation of G4-based molecular devices [16–17] and biosensors [18–23]. Also, there have been promising reports on cellular imaging of G4-DNA [24–29] and G4-RNA [30–33] structures using small-molecule probes. A large number of fluorescent probes for G4-DNA and RNA have thus emerged in the last years, as summarized in several recent reviews on this subject [34–39]. Moreover, novel probes continue to be regularly reported. However, in most cases, the discovery of novel probes is based on serendipitous findings or limited variations of already established fluorogenic scaffolds. This provokes a flood of “one-molecule” papers that report on novel exciting probes, but do not compare their performance with that of already established ones [40–51]. Systematic approaches to the development of fluorescent probes are still rare and explore only a limited range of the chemical space [52–57]. This is a major hurdle to the establishment of solid structure–properties relationships. Therefore, the choice of the best probe for a particular application, as well as the development of novel probes with improved or tailored properties, still remain problematic tasks.

**Figure 1:**
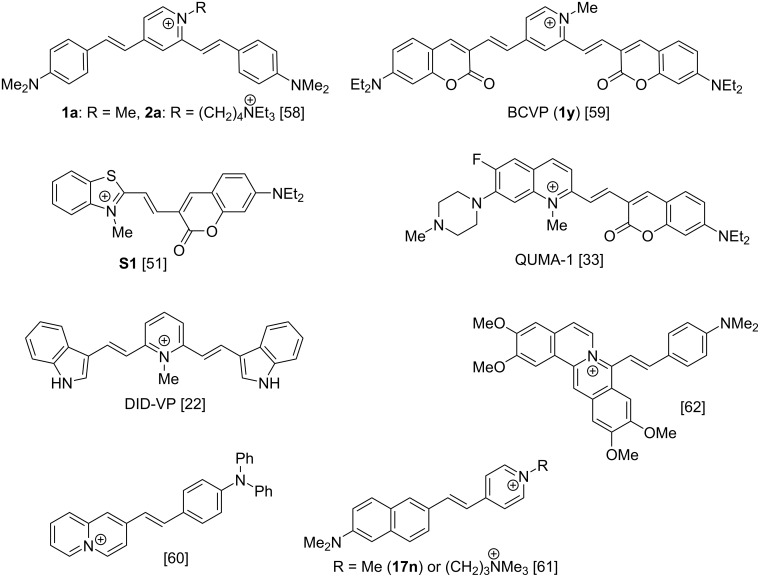
Some di- and mono-styryl dyes previously reported as fluorescent “light-up” probes for G4-DNA and RNA. Counter-ions are omitted for the sake of clarity.

Along these lines, we have previously reported that cationic styryl-type dyes, such as distyrylpyridinium derivatives **1a** and **2a** (Figure 1) represent a promising starting point for the development of fluorescent probes selective for a variety of G4-DNA structures [58]. Another distyryl dye, namely coumarin derivative **1y** (**BCVP**), provides a bimodal (colorimetric and fluorimetric) output towards G4-DNA through the selective disruption of H-aggregates formed in buffered solution [59]. In the meantime, numerous other styryl derivatives were reported as efficient “light-up” probes for G4-DNA and RNA, validating the potential of this molecular scaffold (Figure 1) [22,33,60–63]. Nevertheless, the structural determinants for the desired properties of the probes (i.e., high selectivity for G4-DNA or G4-RNA with respect to double-stranded or single-stranded nucleic acids, high fluorimetric response and quantum yield, low background fluorescence) are still poorly understood, mostly due to the lack of comparative studies. To explore this aspect, we report the synthesis and systematic study of a library of 61 di- and mono-styryl dyes, as potential “light-up” probes for G4 structures. The study aims at the improvement of photophysical properties of the dyes and the establishment of structure–properties relationships.

## Results

### Design and synthesis of the dye library

**Figure 2:**
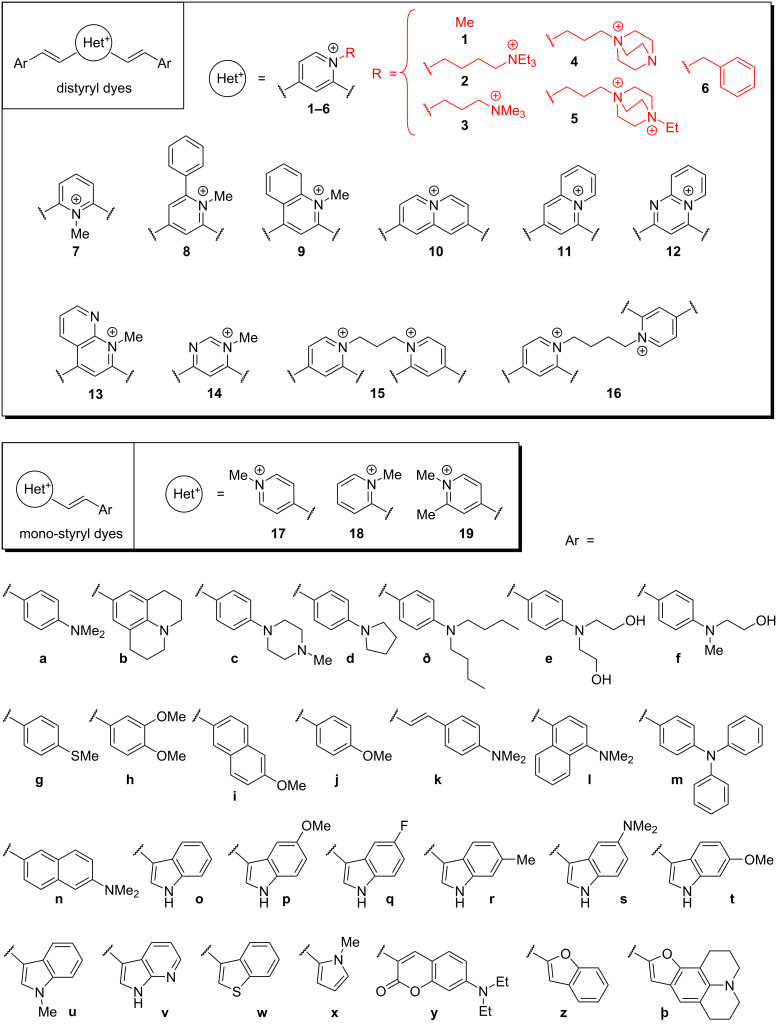
Design of a library of di- and mono-styryl dyes. Counter-ions are omitted for the sake of clarity.

On the basis of the previously established distyryl scaffold, we designed 49 novel derivatives through systematic variation of the electron-donating lateral aromatic groups Ar (**a**–**Þ**), the cationic heterocyclic core Het^+^ (**1**–**16**) and, in the case of 2,4-disubstituted derivatives (**1–6**), the substituent R (Figure 2). Among these, several distyryl dyes (**1o**, **1x, 7x** [64] and **10a** [65]) have been previously reported as fluorescent probes for detection of double-stranded DNA. Compounds **15a** and **16a** are homo-dimeric derivatives, featuring two distyryl moieties connected via a C_3_ (**15a**) or a C_4_ (**16a**) linker. In addition, we included 9 mono-styryl derivatives. Among these, compounds **17a** and **18a** are long-known [66–67]; however, to the best of our knowledge, they have not been studied as fluorescent probes for G4 structures so far. On the contrary, dye **17n** (Figure 1) was reported as a fluorescent probe for G4-DNA during the preparation of the present work earlier this year [61]. Of note, numerous mono-styryl dyes combining indole and quinolinium or pyridinium fragments have been described as bright, photostable stains for double-stranded DNA, although their interaction with G4 structures has not been assessed [68–70].

**Scheme 1:**
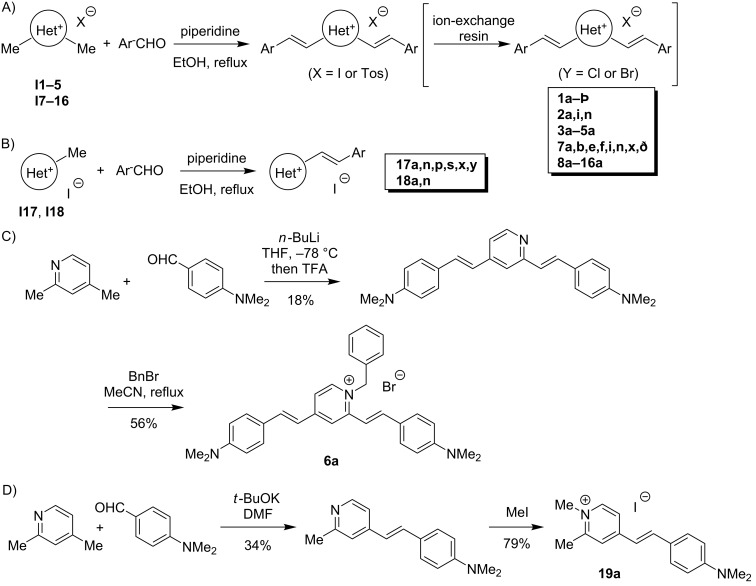
A, B) General synthesis of A) distyryl and B) mono-styryl dyes via Knoevenagel condensation route. C) Synthesis of the dye **6a**. D) Synthesis of the dye **19a**.

**Scheme 2:**
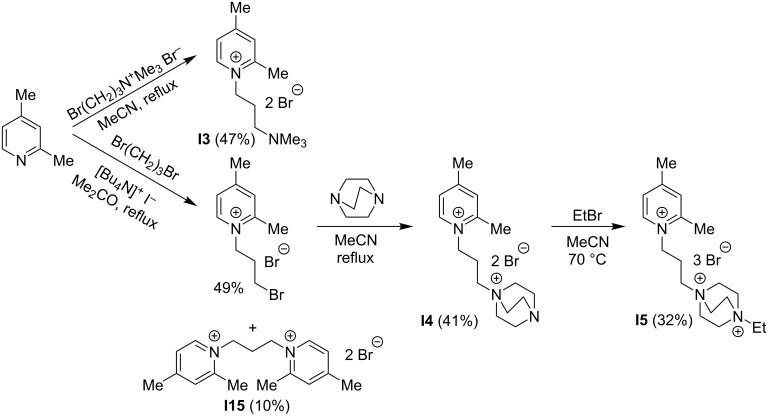
Synthesis of **I3–5** and **I15**.

All dyes, except for distyryl derivative **6a** and mono-styryl derivative **19a**, were obtained through a piperidine-catalyzed Knoevenagel condensation of the corresponding heterocyclic precursors **I1–5** and **I7–16** with 1.5 molar equivalents (per styryl unit) of aromatic aldehydes ArCHO (Scheme 1A,B). The synthesis of precursors **I3–5** and **I15** is presented in Scheme 2 and detailed in Supporting Information File 1. Dyes **6a** and **19a**, which could not be obtained by this route, were synthesized through quaternization of the corresponding neutral styryl precursors with alkyl halides (Scheme 1C,D).

Most dyes of the library were prepared and handled as iodide salts. However, in the case of very lipophilic dyes, the solubility of iodide salts in the high-ionic-strength aqueous buffer required for native G4 structures was insufficient. In these cases, ion exchange to bromide (**1b**, **1ð**, **1u**, **6a**, **7b**, **7n**) or chloride (**1d**, **1k–1q**, **1t**, **1w**, **7ð**, as well as **8a–12a** and **14a–16a**) was performed using ion-exchange resins (cf. Supporting Information File 1 and Table 1), in order to achieve a satisfactory level of solubility in aqueous buffer (i.e., no visible precipitation at a dye concentration of 10 µM in K-100 buffer: 10 mM LiAsO_2_Me_2_, 100 mM KCl, pH 7.2). Dyes containing side-chain substituents (**2a–6a**) were obtained directly as bromide salts, sufficiently soluble in the aqueous buffer. All dyes were rigorously purified by recrystallization and their identity and purity were confirmed by ^1^H and ^13^C NMR, LC–MS and elemental analysis data.

### Optical properties

**Figure 3:**
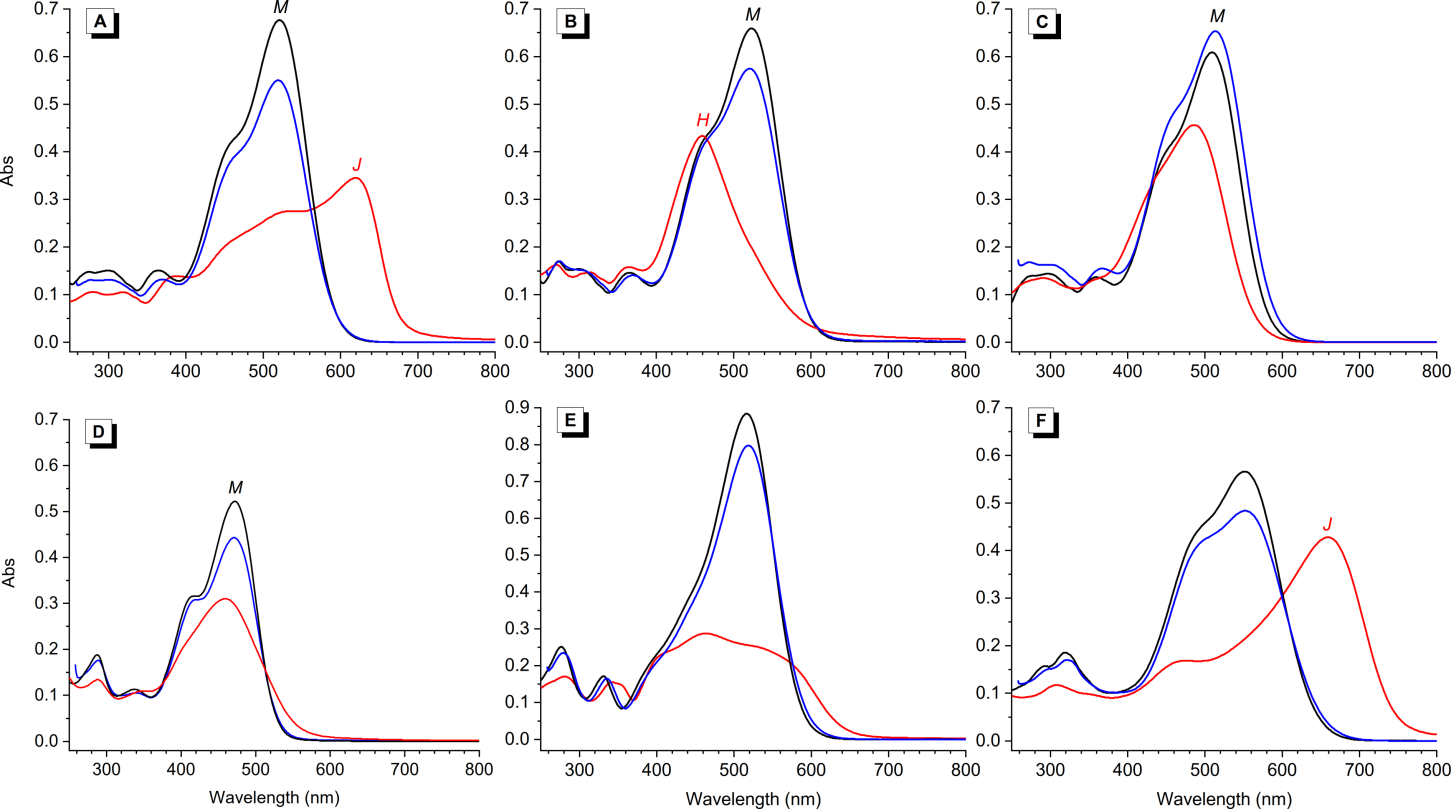
Representative absorption spectra of distyryl dyes: A) **1d**, B) **1ð**, C) **1f**, D) **1u,** E) **10a** and F) **12a** in DMSO (blue), MeOH (black) and K-100 aqueous buffer (red lines); *c* = 10 µM in all cases. Band assignment (when possible): M, monomer; H, H-aggregate; J, J-aggregate.

The library of styryl dyes covers a broad spectral range, with absorption maxima ranging, in MeOH, from 407 nm (**1j**) to 605 nm (**1þ**), and molar extinction coefficients from around 45,000 to 60,000 cm^−1^ M^−1^ (Table 1). Several representative absorption spectra are shown in Figure 3. In aqueous buffer solutions (K-100: 10 mM LiAsO_2_Me_2_, 100 mM KCl, pH 7.2) and at dye concentration of 10 µM, the absorption bands of most dyes are blue-shifted by 10 to 30 nm and undergo a hypochromic effect, compared with organic solvents such as MeOH or DMSO. This behavior evidences a more or less significant aggregation propensity of dyes in aqueous medium, even though, in all tested cases, no visible precipitation occurred. In addition, some dyes (**1c**, **1ð**, **1Þ**, **9a**, **10a**) display even larger (>50 nm) blue shifts of their absorption bands (Figure 3B). This is a characteristic feature for the formation of H-aggregates, as already described for dye **1y** [59]. On the other hand, several dyes displayed new, strong absorption bands, red-shifted by ≈70 nm (**14p**) or more than 100 nm (**1d** and **12a**) in aqueous buffer solution, with respect to organic solvents. These could be ascribed to the formation of J-aggregates (Figure 3A and 3F). This phenomenon was already observed, although at a lower extent, with dye **1a** [58]. With respect to the molecular structure of dyes, it may be concluded that lipophilic substituents (**1c**, **1d**, **1ð**, **1y**, **1Þ**) and/or π-expanded heterocyclic cores (**9a**, **10a**, **12a**, **14p**) promote the dye aggregation, but the nature of the resulting aggregate (H vs J) is unpredictable. Conversely, small or hydrophilic substituents (**1e**, **1f**, **1h**, **1x**, **7e**, **7x**) or charged aminoalkyl chains (**3a**, **4a**) reduce the tendency of the dyes to self-aggregate, as suggested by the reduced hypochromism of their absorption bands in aqueous solutions. Of note, our assessment of the aggregation behavior of the dyes is only preliminary, as it was performed at a single concentration (10 µM) and fixed ionic strength of the medium (110 mM). A complete investigation of this phenomenon is outside the scope of the present work. Finally, as typically observed for styryl dyes, most of the library members displayed very weak fluorescence both in organic solvents (MeOH, DMSO) and in aqueous buffer, as assessed by visual inspection of the respective solutions.

**Table 1:** Positions of maxima and intensity of long-wavelength absorption bands of dyes in MeOH and K-100 aqueous buffer.^a^

Dye	Anion	MeOH		Buffer K-100	
		λ_max_ [nm]^b^	ε [10^3^ cm^−1^ M^−1^]	λ_max_ [nm]^b^	ε [10^3^ cm^−1^ M^−1^]

distyryl dyes					

**1a**	I^–^	507	61.7	476, 616 (sh) (J)	39.9, 1.4
**1b**	Br^–^	551	61.4	510	28.7
**1c**	I^–^	472	53.7	422 (H)	43.6
**1d**	Cl^–^	521	67.7	620 (J), 531 (sh)	34.5, 27.5
**1ð**	Br^–^	524	65.9	459 (H)	43.3
**1e**	I^–^	508	63.7	486	53.6
**1f**	I^–^	510	61.9	486	45.6
**1g**	I^–^	418	50.4	404	40.6
**1h**	I^–^	422	44.6	403	40.8
**1i**	I^–^	425	54.7	414	23.2
**1j**	I^–^	407	46.7	393	393
**1k**	Cl^–^	533	66.4	510	26.3
**1l**	Cl^–^	468	32.8	443	22.4
**1m**	Cl^–^	493	61.0	479	38.3
**1o**	Cl^–^	466, 409 (sh)	56.2, 32.6	458	27.0
**1p**	Cl^–^	474	57.2	465	29.3
**1q**	Cl^–^	457, 404 (sh)	55.3, 34.0	487	27.3
**1r**	I^–^	473, 417 (sh)	56.9, 32.2	462	28.8
**1s**	I^–^	482	33.4	456	24.6
**1t**	Cl^–^	476	55.5	455	20.8
**1u**	Br^–^	472, 418 (sh)	52.2, 31.6	459	31.0
**1v**	I^–^	435, 391 (sh)	48.8, 33.6	413, 367	22.0, 19.9
**1w**	Cl^–^	410	32.8	404	22.5
**1x**	I^–^	473, 419 (sh)	50.9, 32.5	455	42.7
**1y**	I^–^	527	95.3	580, 460 (H)	14.9, 44.0
**1z**	I^–^	423	55.9	416	45.3
**1Þ**	I^–^	605	62.6	542 (H)	30.4
**2a**	2 Br^–^	516	62.9	483	43.1
**2i**	2 Br^–^	430	53.2	411	32.8
**2n**	2 Br^–^	508	56.9	475	28.3
**3a**	2 Br^–^	523	65.1	493	44.9
**4a**	2 Br^–^	524	57.4	493	48.1
**5a**	3 Br^–^	528	64.5	495	43.3
**6a**	Br^–^	520	64.7	491	25.0
**7a**	I^–^	494	70.3	465	44.9
**7b**	Br^–^	535	66.4	507	29.0
**7ð**	Cl^–^	511	79.5	480	43.4
**7e**	I^–^	497	73.5	475	60.2
**7f**	I^–^	497	68.9	474	52.3
**7i**	I^–^	420	62.5	400	20.1
**7n**	Br^–^	489	64.0	480	21.2
**7x**	I^–^	467	57.3	448	48.7
**8a**	Cl^–^	512	62.1	476	38.6
**9a**	Cl^–^	565, 497	56.6, 48.3	492 (H)	33.5
**10a**	Cl^–^	516	87.0	463 (H)	28.7
**11a**	Cl^–^	473	61.1	432	32.8
**12a**	Cl^–^	551	56.7	659 (J), 471	42.8, 16.8
**13a**	Tos^–^	597, 512	53.9, 42.0	570 (sh), 515	24.9, 27.0
**14a**	Cl^–^	569, 471	82.3, 29.7	564	54.4
**14p**	Cl^–^	523, 420	74.4, 20.5	590 (J)	67.5
**15a**	2 Cl^–^	520	115.2	523	61.4
**16a**	2 Cl^–^	510	111.7	490	69.1

mono-styryl dyes					

**17a**	I^–^	475	47.1	448	31.5
**17n**	I^–^	473	37.1	422	29.1
**17p**	I^–^	444	42.3	422	35.5
**17s**	I^–^	449	33.2	424	32.2
**17x**	I^–^	440	35.1	424	24.3
**17y**	I^–^	495	61.2	491	54.1
**18a**	I^–^	461	42.1	435	28.4
**18n**	I^–^	459	33.4	409	26.0
**19a**	I^–^	466	45.9	438	31.2

other					

**ThT**^c^	Cl^–^	415	28.1	410	24.7

^a^10 mM LiAsMe_2_O_2_, 100 mM KCl, pH 7.2. ^b^sh: shoulder, H: H-aggregate band, J: J-aggregate band. ^c^Thioflavin T.

**Figure 4:**
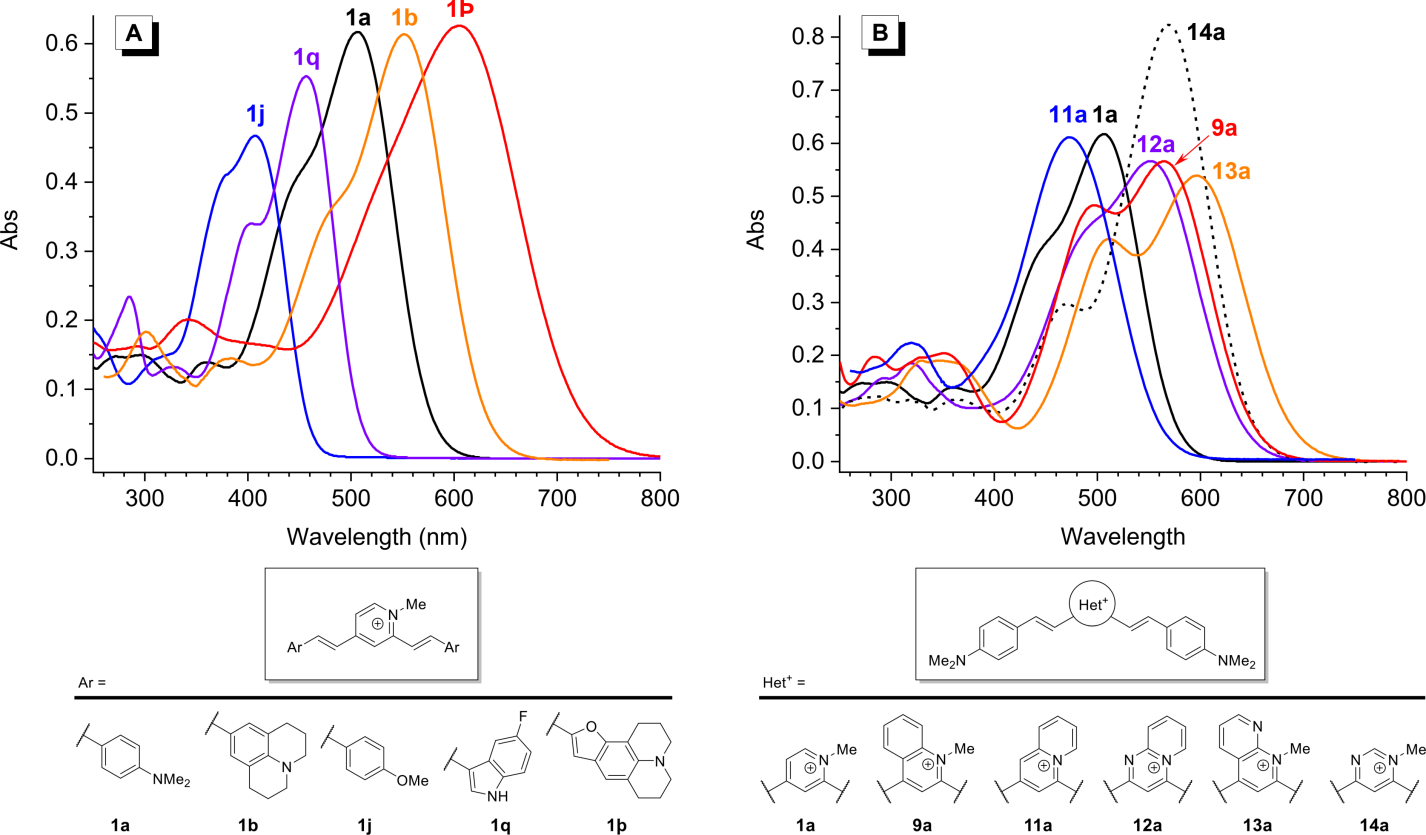
Representative absorption spectra of the distyryl dyes (*c* = 10 µM in MeOH) demonstrating the influence of the molecular structure on the optical properties. A) Variation of Ar groups. B) Variation of the Het^+^ core.

In non-aggregating conditions, the influence of the molecular structure of the dyes on their absorption bands can be clearly observed. Thus, when Ar contains poor electron-donating substituents (**1g–1j**), the absorption spectra of the dyes are blue-shifted with respect to the prototype dye **1a**. Conversely, strongly electron-donating (**1b**, **1d**) or π-extended (**1k**, **1Þ**) Ar units lead to bathochromic shifts of absorption bands (Table 1 and Figure 4A). The influence of the heterocyclic core is equally important: replacement of the 2,4-pyridinium unit in dye **1a** with a 2,6-pyridinium (**7a**) or a 2,4-quinolizinium moiety (**11a**) leads to a blue shift of the absorption maximum, whereas all other heterocyclic units lead to significantly stronger (**10a**, **14a**) and/or red-shifted (**9a**, **12a**, **13a**) absorption bands (Table 1 and Figure 4B). On the other hand, the nature of the substituent R in the 2,4-pyridinium unit has only a minor influence on the optical properties, and the absorption bands of the dyes **2a–6a** are only slightly red-shifted (by 10–20 nm in MeOH) with respect to that of **1a**.

### Fluorimetric response of dyes towards DNA and RNA structures

The fluorimetric response of the dye library was investigated against a set of 14 diverse nucleic acid structures (Table 2), including ten G4-DNA structures of different topologies (parallel G4: *c-kit2*, *25CEB*, *c-kit87up*, *c-myc, c-src1*; parallel dimer G4: *c-myb;* hybrid G4: *22AG*, *46AG*; antiparallel G4: *TBA*, *HRAS*), two G4-RNA structures (*TERRA* and *NRAS*), as well as genomic double-stranded DNA (calf thymus DNA, *ct DNA*) and RNA from calf liver (*cl RNA*). Screened samples contained fixed concentrations of dyes (2.5 µM) and nucleic acids (5 µM). Corrections to the concentration of nucleic acids were made to take into account the peculiarities of some of the samples. In particular, *46AG* was tested at 2.5 µM, to account for its dimeric G4 nature, and *ct DNA* and *cl RNA* were tested at 110 µM nucleotide concentration, which is equivalent to the total nucleotide concentration in a 5 µM sample of a 22 nt oligonucleotide. All samples were prepared in a K^+^-rich buffer solution (K-100, see Table 1 footnote). Thioflavin T (ThT), which is widely used for detection of G4 structures, was included for comparison. The fluorescence intensity was measured using a microplate reader. In order to screen a large panel of dyes against a number of analytes, the measurements were performed at fixed excitation and emission wavelengths, selected with a set of filters and adapted to the absorption and emission characteristics of each dye (Supporting Information File 1, Table S1).

**Table 2:** Nucleic acid samples used in the first screening round.

Acronym	Sequence (5′ → 3′)	Topology	Number of G-tetrads	Ref.

G4-DNA				

*c-kit2*	GGGCGGGCGCGAGGGAGGGG	parallel	3	[71]
*25CEB*	AGGGTGGGTGTAAGTGTGGGTGGGT	parallel with a long loop	3	[72]
*c-kit87up*	AGGGAGGGCGCTGGGAGGAGGG	parallel with a snap-back loop	3	[73]
*c-myc*	TGAGGGTGGGTAGGGTGGGTAA	parallel	3	[74]
*c-src1*	GGGCGGCGGGCTGGGCGGGG	parallel	3	[75–76]
*c-myb*	GGAGGAGGAGGA	parallel (dimer)	2	[77]
*22AG*	A(GGGTTA)_3_GGG	hybrid (mixture of isoforms)	3	[78]
*46AG*	A(GGGTTA)_7_GGG	hybrid (dimeric G4)^a^	2 × 3	[79]
*HRAS*	TCGGGTTGCGGGCGCAGGGCACGGGCG	antiparallel	3	[80]
*TBA*	GGTTGGTGTGGTTGG	antiparallel	2	[81]

G4-RNA				

*TERRA*	r(AGGGUUAGGGUUAGGGUUAGGGU)	parallel	3	[82]
*NRAS*	r(GGGAGGGGCGGGUCUGGG)	parallel	3	[83]

controls				

*ct DNA*	calf thymus DNA	double-stranded DNA	N/A	
*cl RNA*	calf liver RNA	single-stranded RNA	N/A	

^a^Used at half of the oligonucleotide concentration with respect to other G4 samples.

**Figure 5:**
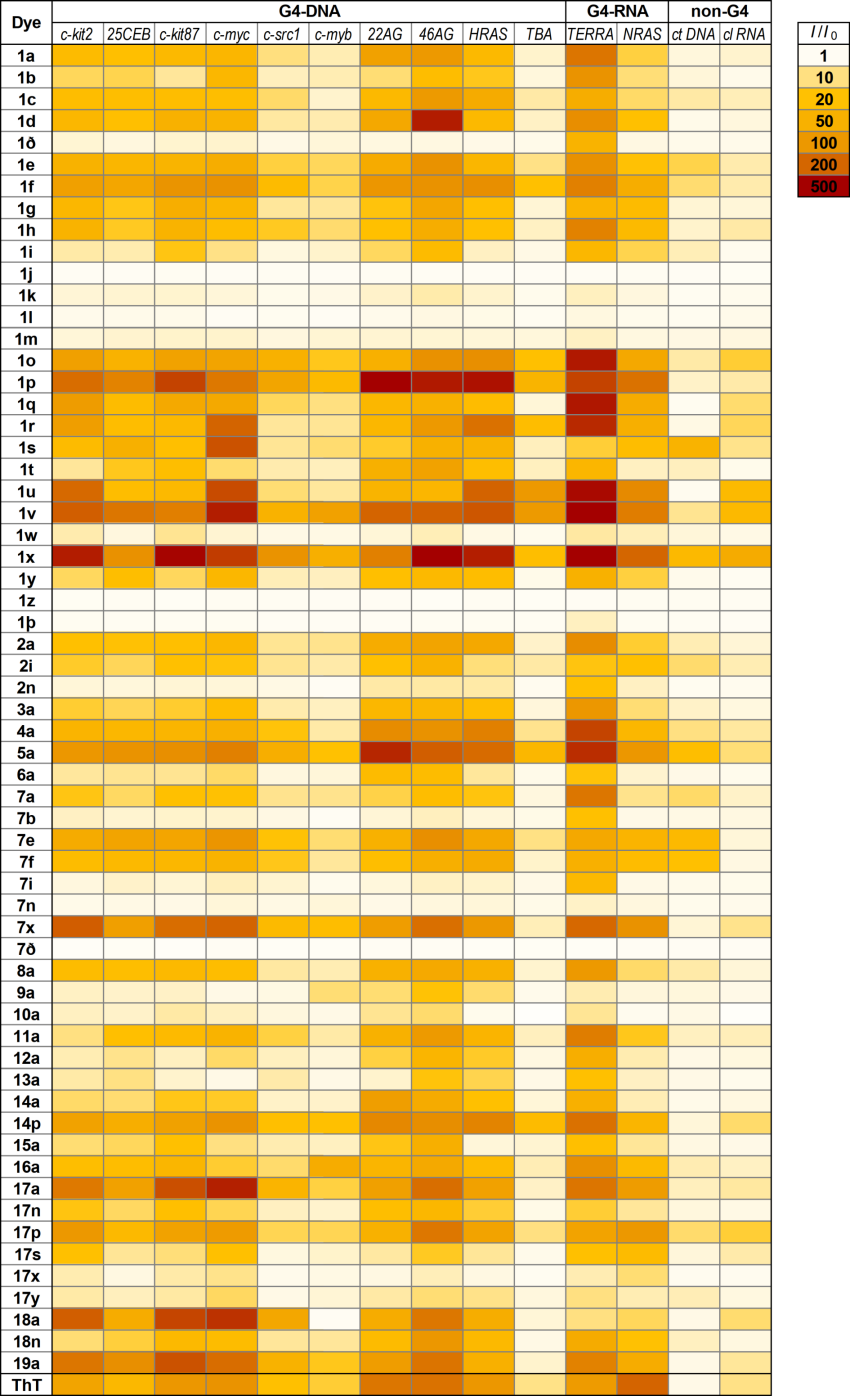
Heat map of the relative emission intensity enhancement (*I*/*I*_0_) of styryl dyes and thioflavin T (ThT) (*c* = 2.5 µM in K-100 buffer) in the presence of 2 molar equiv of G4-DNA (46AG: 1 molar equivalent), G4-RNA, or ct DNA and cl RNA controls used at equivalent nucleotide concentration. Darker cells indicate higher *I*/*I*_0_ values (see legend). For the numeric data, excitation and emission wavelengths see Supporting Information File 1, Table S1.

**Figure 6:**
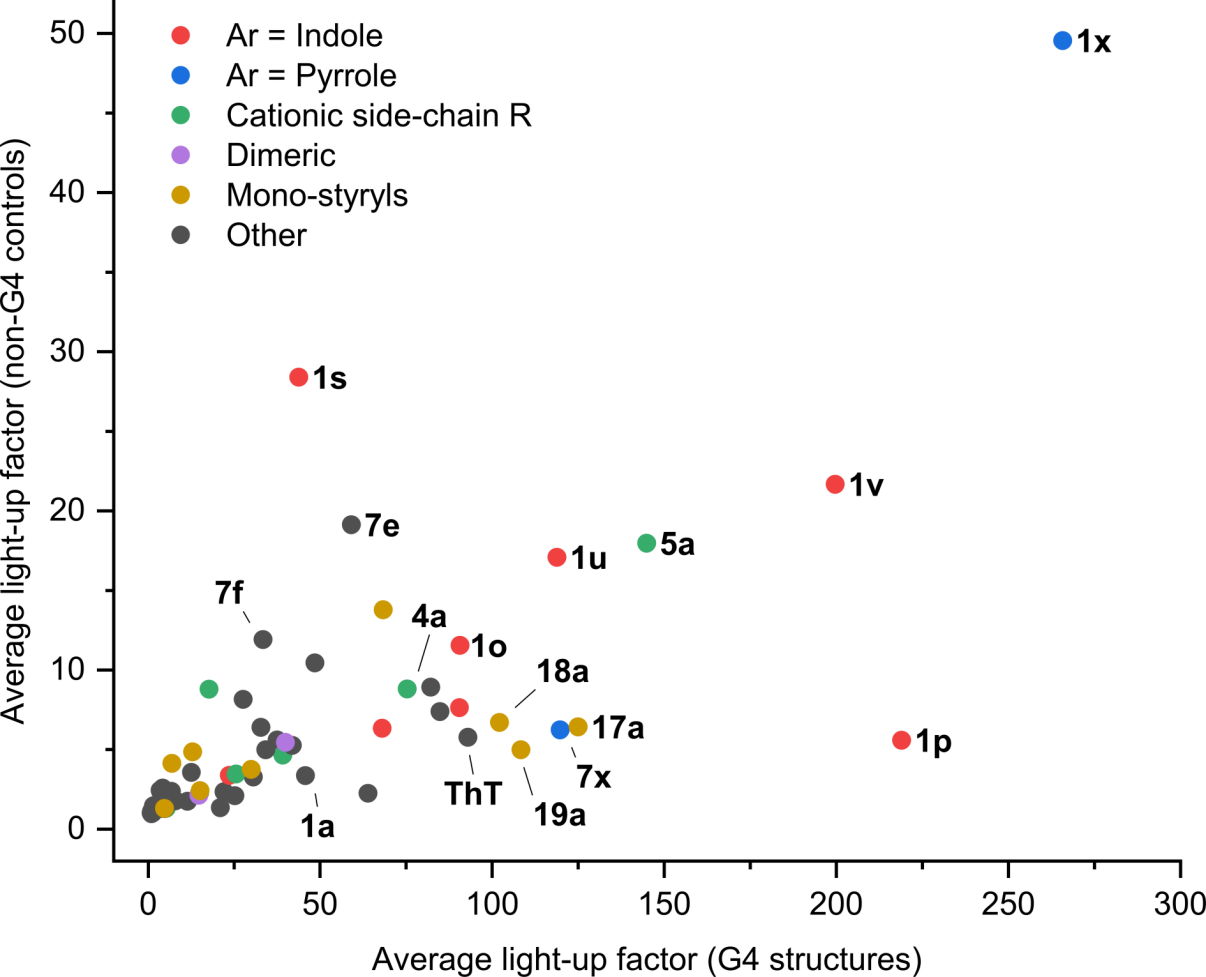
Analysis of the light-up response matrix of the dyes. The average light-up factor of each dye with respect to non-G4 targets (*ct DNA* and *cl RNA*) is plotted against the corresponding average light-up factor obtained with 12 G4 targets. The grouping of dyes is arbitrary and serves to highlight some of the structural motifs. Of note, *x* and *y* axes display different scales.

The results of the screening, presented as relative enhancement of fluorescence intensity in the presence of nucleic acids (*I*/*I*_0_, where *I* is the fluorescence intensity of the dye in the presence of two equivalents of nucleic acid and *I*_0_ is the fluorescence of the dye alone), are shown in the form of a heat-map in Figure 5 (for numeric values cf. Supporting Information, Table S1). In addition, group-average data, i.e., average fluorescence response of each dye towards 12 G4 (DNA and RNA) analytes vs average response to non-G4 (*ct DNA* and *cl RNA*) controls, are presented in Figure 6. This plot facilitates the identification of the most promising probes, disregarding the differences in response of dyes with respect to individual analytes within each group. The inspection of these data leads to a number of interesting observations. 1) Most dyes of the library display significant fluorescence enhancement (*I*/*I*_0_ > 10) in the presence of at least one DNA or RNA target. Only 9 of 61 styryl dyes (**1j**–**m**, **1w**, **1z**, **1þ**, **7n** and **7ð**) displayed weak or no fluorescence enhancement with all nucleic acid analytes. 2) Most remarkably, the fluorescence of all dyes, with the exception of a few most “unresponsive” ones (**1j**, **1l, 1z**, and **7ð**), is preferentially enhanced in the presence of G4-DNA or G4-RNA structures, although to a varying extent. In fact, among the 61 tested dyes, none showed preferential response to double-stranded DNA (ct DNA) or single-stranded RNA (cl RNA) controls. 3) Compared to the prototype dye **1a**, modifications of the core (Het^+^) unit (**7a–14a**) within the distyryl scaffold do not produce significant variations in the fluorimetric response of the dyes. The same holds true for the homo-distyryl compounds **15a** and **16a**, which do not outperform dye **1a**. 4) Likewise, in the 2,4-pyridinium series of dyes, introduction of an aminoalkyl (**3a**) or benzyl substituent (**6a**) does not significantly improve the performance of the probes, as was already described for the dye **2a** [58]. Instead, introduction of a DABCO fragment (bringing two additional positive charges) in **4a** and **5a** leads to higher fluorimetric response of the probes to G4-DNA (e.g., for **5a**, *I*/*I*_0_ = 330 with *22AG*), although accompanied by a concomitant loss of selectivity with respect to ds DNA (*I*/*I*_0_ = 25 for **5a**). 5) In contrast, modification of Ar units strongly influences the fluorimetric response of the dyes. In particular, dyes containing indole residues (**1o**–**s**, **1u** and **1v**; red dots in Figure 6) show particularly large fluorescence enhancement in the presence of most G4-DNA and G4-RNA targets (**1p**: up to 550-fold with *22AG*) and thus represent a significant improvement with respect to dye **1a** (*I*/*I*_0_ < 170, with all analytes) and ThT (*I*/*I*_0_ ≤ 200, with all analytes). Distyryl dyes containing pyrrole residues (**1x**, **7x**: blue dots in Figure 6) also demonstrate outstanding fluorescence enhancement in the presence of G4-DNA analytes (**1x**: up to 690-fold with *TERRA*; **7x**: up to 220-fold with *c-kit2*). However, in the case for **1x**, a marked loss of selectivity with respect to non-G4 analytes can be observed (*I*/*I*_0_ = 40 in the presence of *ct DNA* and 60 in the presence *cl RNA*; cf. Figure 6). Conversely, as mentioned above, the dyes containing benzothiophene (**1w**) or benzofuran (**1z**, **1þ**) residues perform poorly as fluorescent probes. 6) Strongly aggregating dyes (i.e., **1d**, **1ð**, **10a** and **12a**) generally do not show higher light-up effects than weakly aggregating analogues **1a** or **1e**. As a remarkable exception, dye **1d** shows strong and highly selective response towards the dimeric G4-DNA *46AG* (*I*/*I*_0_ = 350), which can be attributed to higher-affinity binding of the dye at the interface between two G4 units, leading to efficient disaggregation. 7) Finally, several mono-styryl dyes, especially **17a**, **17p**, **18a** and **19a** also display significant fluorescence enhancements in the presence of G4 structures (e.g., **17a**: up to 340-fold, **18a**: up to 300-fold, both in the presence of *c-myc*), even higher than those of the distyryl analogues **1a** and **7a**, and good selectivity with respect to double-stranded DNA.

**Figure 7:**
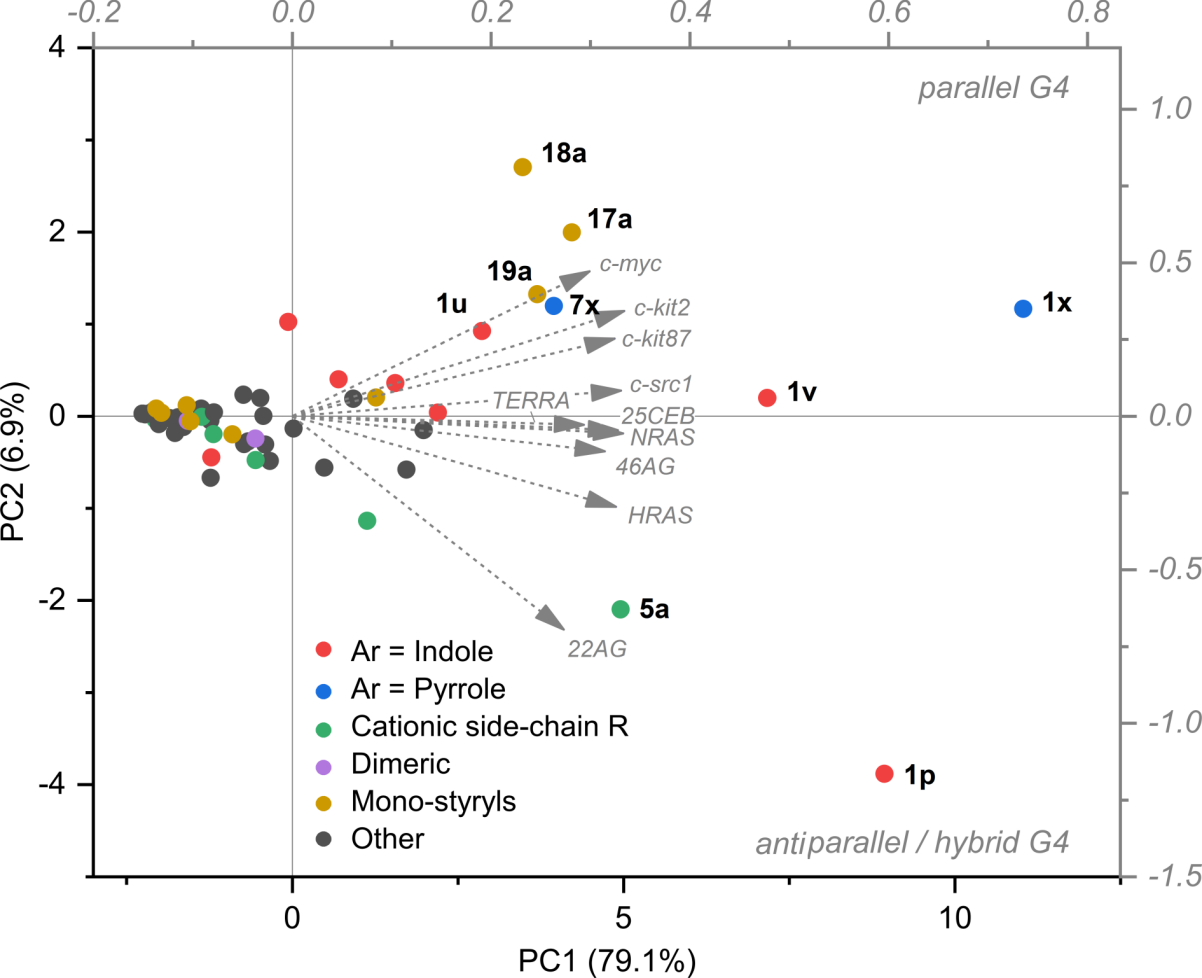
PC1 vs PC2 plot obtained from the principal component analysis of the light-up data matrix for all dyes (*ct DNA* and *cl RNA* were excluded, as well as TBA and *c-myb* due to their low light-up factors). Color coding highlights the distribution of the dyes sharing similar structural motifs.

A number of patterns could also be revealed with respect to the differential response of the probes towards various G4 targets. 1) Remarkably, G4-RNA *TERRA* systematically induces the highest fluorimetric response of most probes from the distyryl series, but not from the mono-styryl one. 2) In contrast, *TBA* and *c-myb* (i.e., both two-quartet quadruplexes) are poorly detected by most dyes (including ThT), in agreement with what was observed with other probes [6,14,57]. Nonetheless, several indole-containing dyes enable sensitive detection of these targets (with *I*/*I*_0_ up to 100, **1u** and **1v**), with an excellent selectivity with respect to double-stranded DNA. 3) Several dyes display preferential response towards one or another topological group of analytes. The first group (dyes **1a**, **1d**, **1p**, **1x**) is selective towards hybrid (*22AG*, *46AG*) and antiparallel (*HRAS*) G4-DNA, whereas the second group (dyes **1s**, **1u**, **1v**, **17a** and **18a**) shows fluorimetric selectivity for parallel G4-DNA forms (*c-kit2*, *c-kit87up*, *c-myc*). To verify the preferences of the dyes with respect to the conformation of the G4 analytes, we analysed the data matrix presented in Figure 5 using principal component analysis (PCA). *TBA* and *c-myb,* which had proven mostly unresponsive, were excluded from the analysis. The response pattern of each dye is represented as a dot in the plot of the two first principal components (PC1 vs PC2, Figure 7). In this plot, PC1 (*x* axis) correlates with the overall light-up intensity observed for each dye with the tested targets. On the contrary, PC2 (*y* axis) correlates with the intra-G4 selectivity of each compound, with compounds selective for hybrid and antiparallel G4s locating in the lower part of the plot and compounds selective for parallel G4s locating in the upper part. Interestingly, the loading vectors for parallel G4-RNA (*NRAS* and *TERRA*) fall in between those of parallel and hybrid/antiparallel DNA G4s, suggesting an impact of the ribose backbone on the interaction. As can be inferred from the dot distribution in the plot, the mono-styryl motif and the pyrrole substituent within the distyryl motif (**1x**, **7x**) clearly promote the selectivity for the parallel G4 structures. On the other side, the effect of the indole motif is less clear, with most of the dyes not displaying any well-defined preference, except for **1p**. This latter compound displays a marked selectivity for hybrid and antiparallel topologies, and in particular for the *22AG* target.

### Topological classification of G4-DNA by dual-dye analysis

On the basis of screening results, two dyes, namely **1p** and **18a**, were selected to build a fluorimetric test for conformational classification of DNA oligonucleotides. As discussed above, they present complementary preferences with respect to the analyte groups, with **1p** preferentially responding to hybrid and **18a** to parallel G4-DNA structures. Moreover, both dyes showed excellent light-up response and selectivity for G4 targets over controls. The concomitant analysis of the response of both dyes should thus allow the sensitive discrimination of different G4 topologies. The response of two dyes was tested against a panel of 33 DNA analytes (Supporting Information File 1, Table S2), comprising some of the previously tested oligonucleotides (*c-myc*, *25CEB*, *22AG*, *46AG*, *TBA*, *ct DNA*). Altogether, the panel of analytes comprehended five conformational groups of roughly equal size, representing the three G4 topologies (parallel, antiparallel and hybrid) as well as single and double strands. RNA targets were excluded from this experiment, even though G4-RNA TERRA triggered the highest fluorescence responses for many dyes. On one side, the interest of G4-RNA topology investigation is limited. In fact, to date, they have never been shown to adopt a structure other than parallel one [84–85]. On the other side, PCA proved that the response of the dyes to RNA targets differs from that to parallel G4-DNA, which might complicate data interpretation.

**Figure 8:**
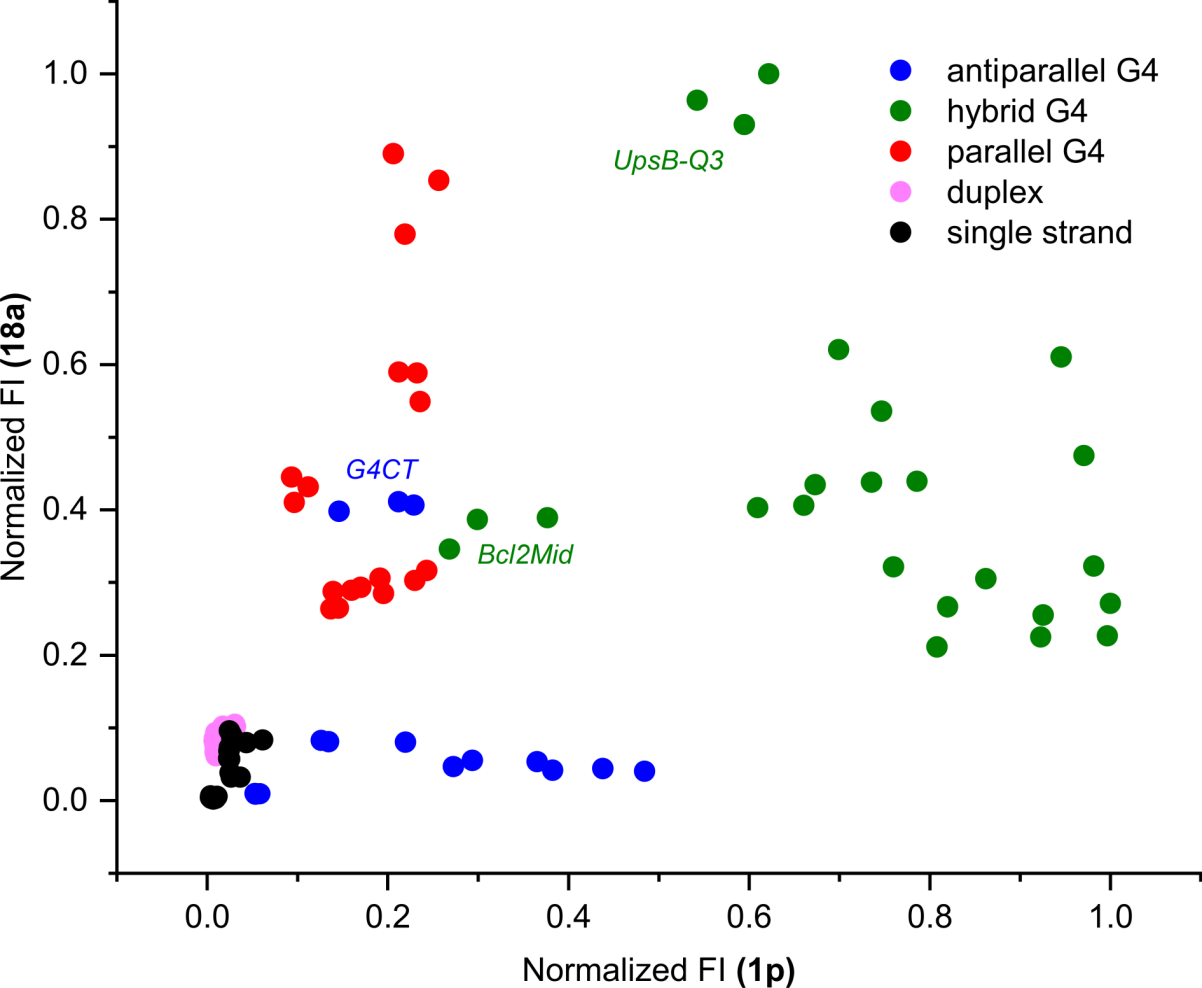
Dual-dye conformational analysis of an extended panel of 33 DNA oligonucleotides. This is performed on normalized datasets (data referring to each of the two dyes are normalized separately), plotting the resulting fluorescence of compound **18a** against that of compound **1p**. Data for each target are presented as independent triplicates.

Emission intensities of both dyes were measured for the new DNA panel in the conditions previously utilized for the screening (2.5 µM dye, 5 µM DNA oligonucleotide, K-100 buffer). The data points corresponding to the oligonucleotides in the set are displayed in a 2D scatter plot (Figure 8), featuring normalized emission intensities of **1p** and **18a** dyes as *x* and *y* axes, respectively. Notably, the oligonucleotides appeared to be grouped in clusters broadly mirroring their conformations. Specifically, parallel G4s cluster in the upper left part of the plot (red dots), as a result of high fluorescence response with **18a** and moderate-to-low response with **1p**. Hybrid G4s (green dots) produce moderate light-up values for **18a** and high ones for **1p**, thus clustering on the right side of the plot. Finally, antiparallel G4s (blue dots) locate in the lower left part of the plot, corresponding to almost null emission enhancement by **18a** and low one by **1a**. Despite the low response to antiparallel G4 structures, these can be still clearly distinguished from double- and single-stranded controls (pink and black dots), to which none of the two dyes proves responsive. A few G4 structures located relatively far from the areas occupied by the respective groups. This is the case of *G4CT*, *Bcl2Mid* and, at least partially, *UpsB-Q3*. In the case of *G4CT*, previous studies report the existence of an equilibrium between a monomolecular antiparallel form and a bimolecular parallel one, affected by K^+^ and oligonucleotide concentration [86]. As already suggested, this oligonucleotide is probably present as a mixture of conformations in our working conditions [14]. It is thus likely that the parallel one strongly influences the position of the data in the plot, being better stained by dye **18a**. For both *Bcl2Mid* and *UpsB-Q3*, CD spectra are partially different from those obtained with typical hybrid G4s, normally related to the telomeric sequence [14]. This might indicate the presence of peculiar structural elements that might as well play a role in determining the probes response. On the overall, the combination of the two probes proved quite efficacious at both (1) distinguishing G4 forming DNA sequences from controls, comprehending randomly generated single strands with varying content of guanine and a wealth of duplex structures, and (2) discriminating G4 structures based on their topology, with the exception of a few notable cases presenting structural peculiarities.

### Quantum yield and brightness of the probes

**Figure 9:**
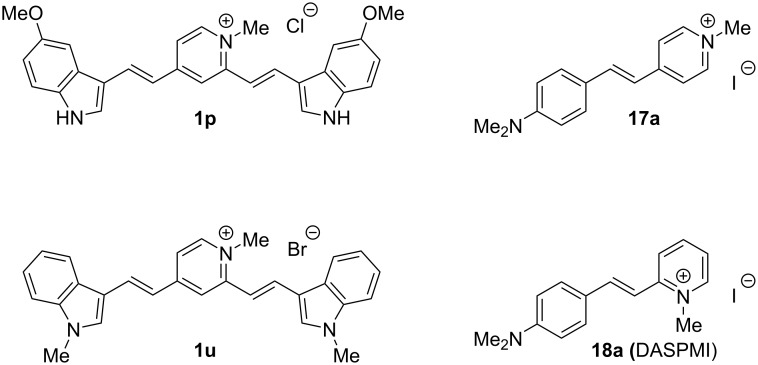
Selected probes featuring high fluorimetric response towards G4 structures.

**Figure 10:**
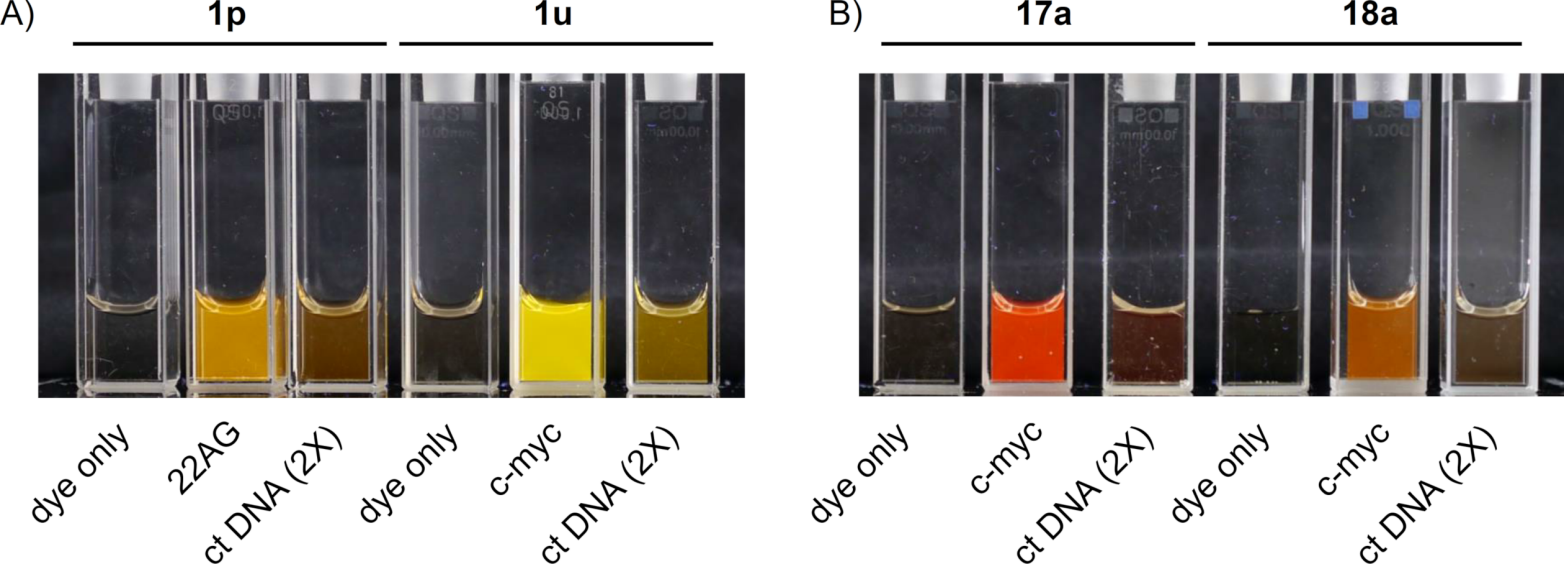
Photographs of solutions of A) distyryl dyes **1p** and **1u**; B) mono-styryl dyes **17a** and **18a**, in the absence or in the presence of G4 (*22AG* or *c-myc*, 10 µM) or ds DNA (ct DNA, 250 µM bp) upon bottom illumination with UV light (*λ* = 312 nm); in all cases, *c*(dye) = 5 µM in K-100 buffer.

Four highly responsive and G4-selective dyes, namely **1p**, **1u**, **17a** and **18a** (Figure 9), were chosen for fluorescence quantum yield and brightness measurements, in order to assess their potential for imaging applications. The quantum yield of dyes was measured in the 1.2–3 µM concentration range, in the absence or in the presence of an excess of two G4-DNA analytes, namely *c-myc* (parallel G4) or *22AG* (hybrid G4), and brightness data were obtained from the multiplication of the corresponding quantum yield by the molar absorptivity coefficient at absorption maxima (ε_max_) values, for dyes alone and dye–G4 complexes. The obtained data are presented in Table 3. The images of dyes in the absence and in the presence of selected DNA samples are shown in Figure 10.

**Table 3:** Optical parameters (absorption and emission maxima, Stokes shift, molar absorptivity coefficient at the absorption maximum, fluorescence quantum yield and brightness) of dyes **1p**, **1u**, **17a** and **18a** in the absence of DNA or in the presence of G4-DNA structures (*c-myc* and *22AG*).

Properties	Conditions^a^	**1p**	**1u**	**17a**	**18a**

λ_max_ (abs) [nm]^b^	*c-myc*	501	504	463	450
	*22AG*	508	497	455	447

λ_max_ (em) [nm]^c^	*c-myc*	571	560	611	584
	*22AG*	570	560	606	582

∆λ [nm]^d^	*c-myc*	70	56	148	134
	*22AG*	62	63	151	135

ε_max_ [10^3^ cm^−1^ M^−1^]^e^	no DNA	29.3	31.0	31.5	28.4
	*c-myc*	32.6	35.7	26.8	24.3
	*22AG*	30.6	29.2	28.6	26.6

Φ^f^	no DNA	0.0041	0.0114	0.0015	0.0056
	*c-myc*	0.124	0.319	0.085	0.079
	*22AG*	0.227	0.293	0.047	0.040

*B* [10^3^, cm^−1^ M^−1^]^g^	no DNA	0.12	0.35	0.047	0.16
	*c-myc*	4.04	11.4	2.27	1.92
	*22AG*	6.95	8.56	1.34	1.06

^a^Whenever indicated: in the presence of 6 µM of the respective G4-DNA. ^b^Absorption maximum in K-100 buffer, in the concentration range of 1.2–3 µM. ^c^Fluorescence emission maximum (λ_ex_ = 500 nm). ^d^Stokes shift. ^e^Molar absorptivity coefficient at the absorbance maximum for the dye alone or the dye-G4 complex. ^f^Fluorescence quantum yield (integration range: 510–800 nm, reference: rhodamine 6G in EtOH). ^g^Brightness (*B* = ε_max_ × Φ).

All dyes display moderate to good quantum yields and brightnesses upon complexation with both G4 analytes. In more detail, distyryl dyes **1p** and **1u** prove more performant in this sense, displaying higher quantum yield and brightness (Φ = 0.12 to 0.32, *B* = (4.0–11.4) × 10^3^ cm^−1^ M^−1^) than mono-styryl dyes (Φ = 0.040–0.085, *B* = (1.1–2.3) × 10^3^ cm^−1^ M^−1^). It must be noted that the increase of fluorescence quantum yields observed in the presence of G4-DNA (Φ/Φ_0_) is significantly lower (up to five-fold) than the corresponding light-up factor measured at a single wavelength (Table S1, Supporting Information File 1). This is typically due to red shifts of absorption and emission spectra of dyes in the presence of nucleic acids, leading to the overestimation of the light-up effect. Nevertheless, single-wavelength light-up factors are of primordial importance for imaging applications, which are performed with a single set of excitation of emission filters. As can be inferred from the data in Table 3, Φ values mirror the selectivity patterns already observed in the screening experiments. In fact, dye **1p** in the presence of hybrid G4 (*22AG*) is roughly twice as fluorescent as its complex with *c-myc* (Φ = 0.227 and 0.124, respectively). On the other side, complexes of **1u**, **17a** and **18a** with *c-myc* are more fluorescent than those with *22AG*. Among these, **1u** certainly forms the brightest and most fluorescent complexes (Φ = 0.32 and 0.29; *B* = 11.4 and 8.6 × 10^3^ cm^−1^ M^−1^ for *c-myc* and *22AG*, respectively). However, the differences observed between the two G4 analytes are narrow, with respect to those observed with the other dyes.

The analysis of absorption spectra also allowed us to quantify the red-shift effect observed for all dyes upon complexation to G4 structures, which is more pronounced for distyryl dyes **1p** and **1u** (36–45 nm, cf. Table 1 and Table 3) than for mono-styryl dyes **17a** and **18a** (7–15 nm, cf. Table 1 and Table 3). Moreover, comparison of the absorption maxima with the corresponding emission ones enabled us to calculate the Stokes shifts for the samples. Of note, all compounds display remarkable shifts, ranging from 56 to 151 nm, although these are more pronounced for distyryl dyes (142 nm on average) than for mono-styryl ones (63 nm on average). The combination of the absorption maxima redshift and these high Stokes shifts contributes to make the selected styryl dyes excellent tools for optical imaging.

## Discussion

Despite the wealth of scaffolds already reported for the fluorimetric detection of G4 structures, the published studies usually lack a systematic investigation of the factors governing their interaction with DNA and sensing capabilities. In fact, although it is known in broad terms that some molecular features (e.g., size and shape of the aromatic scaffold, charge, redox potential) influence the interaction of dyes with G4-DNA, a thorough assessment of such phenomena by comparative studies is lacking in most reports. In this work, we address this gap within the family of styryl dyes, trying to establish how to construct an optimized dye for G4 sensing. In particular, we studied the optical properties and the fluorimetric response of 61 in-house synthesized compounds against a set of G4-DNA and G4-RNA analytes, as well as the respective non-G4 controls. The data were analyzed aiming at the identification of structural motifs or physical properties (such as aggregation in aqueous medium) of dyes which could govern their fluorimetric response towards one or another group of analytes. Most remarkably, our results demonstrated that a large majority of the dyes (57 out of 61) undergo preferential fluorescence enhancement in the presence of G4 structures, compared with double-stranded (DNA) and single-stranded (RNA) controls (the remaining four dyes did not undergo a fluorescence enhancement with any of the analytes). Can it be considered as a general rule? Considering the significant structural diversity of our library and the related works [41,49], this is highly probable, with regard to mono- and distyryl scaffolds. This implies that styryl-based fluorescent probes initially developed for detection or visualization of DNA, RNA, or other analytes, either in vitro or in cellular imaging applications, must be reassessed in view of their potential strong bias for G4 motifs. Indeed, a remarkable “light-up” effect of SYPRO Orange, a widely used protein stain belonging to the mono-styryl dye family, in the presence of G4-DNA has been reported earlier this year [49]. Moreover, mono-styryls **17a** and **18a** (the latter also known as DASPMI) are long-known and widely used as mitochondrial stains [87–88] and groove-binding fluorescent probes for double-stranded DNA [89–90]. Herein, we report that the fluorescence enhancement of these dyes induced by parallel G4 structures is dramatically higher compared to ds DNA.

Although the preferential response to G4 structures seems to be an inherent feature of the styryl scaffold, the magnitude of the “light-up” effect drastically varies within the series. Our results clearly point to several structural motifs that appear advantageous for high fluorimetric response and high quantum yield of the probes. First of all, indole substituents, including core-substituted indoles, emerge as the most efficient in this sense, as demonstrated by several distyryls (**1o**–**v**) with superior properties with respect to the prototype compound **1a**. A similar effect of indole substituents was already observed in the family of mono-styryl dyes developed for detection of double-stranded DNA [68,70,91]. Moreover, pyrrole-substituted distyryls (**1x** and **7x**) also display very high fluorimetric response (up to *I*/*I*_0_ = 690, for **1x**–TERRA complex), albeit at the expense of somewhat lower selectivity with respect to ds DNA and ss RNA. It may be suggested that electron-rich heterocyclic substituents (indole and pyrrole) act by lowering the reduction potential of dyes, rendering the photoinduced electron-transfer reaction with guanine residues in DNA energetically disfavored and resulting in higher fluorescence quantum yields. However, in the absence of redox potential data, this assumption could not be experimentally verified. Finally, we showed that the mono-styryl design can yield probes with interesting properties, such as high light-up factors (up to 340, for **17a**–*c-myc* complex) and a clear-cut selectivity for parallel-stranded G4 motifs. Interestingly, in the mono-styryl family, dyes containing indole (**17p**, **17s**) or pyrrole (**17x**) substituents did not perform better than the simplest styryl derivatives, i.e., **17a** and **18a**. This fact demonstrates that our data are still insufficient to formulate generalized structure–properties relationships.

## Conclusion

To summarize, a systematic analysis enabled us to select the optimal probes within the styryl dye family (i.e., those displaying high quantum yield and brightness, excellent light-up factor, and remarkable selectivity for a certain G4 class). A comparison with literature data demonstrates that dyes **1p** and **1u** largely outperform, in terms of brightness and quadruplex-vs-duplex selectivity, the widely used fluorescent probes, such as thioflavin T (ThT, Φ = 0.25, in the presence of *22AG*/K^+^ conditions) and thiazole orange (TO, Φ = 0.19 in the presence of *22AG*/K^+^ conditions) [92], and approach the brightest G4-DNA probes developed so far, such as trialryimidazole IZCM-7 (Φ = 0.52, in the presence of *c-myc*) [52] and the NIR-emitting squaraine dye CAS-C1 (Φ of up to 0.74 with parallel G4-DNA) [93]. Applications of these dyes can be multiple. As an example, we proposed herein the implementation of a simple two-dye array to classify G4-DNA structures based on their topology. Applications in the design of G4-based logic gates could also be envisaged. Taking into account the favorable optical properties, in particular high brightness and large Stokes shift, the same probes could be utilized to proceed to cellular imagining of G4 structures, certainly with caution regarding the inherent propensity of cationic dyes to accumulate in mitochondria. At the same time, our work establishes an approach to optimize the structure of renowned scaffolds and achieve maximal performances in G4 sensing.

## Supporting Information

File 1 Experimental details and supplementary Tables S1 and S2.
