# Supplementary material for: Identification of optimal fluorescent probes for G-quadruplex nucleic acids through systematic exploration of mono- and distyryl dye libraries
Source: Beilstein J Org Chem. 2019 Aug 6;15:1872–89. doi: 10.3762/bjoc.15.183 (PMC6693400; doi:10.3762/bjoc.15.183)
Supplement: File 1 — Experimental details and supplementary Tables S1 and S2. [file Beilstein_J_Org_Chem-15-1872-s001.pdf]

BEILSTEIN JOURNAL OF ORGANIC CHEMISTRY

Supporting Information

for

Identification of optimal fluorescent probes for G-quadruplex nucleic acids through systematic exploration of mono- and distyryl dye libraries

Xiao Xie, Michela Zuffo, Marie-Paule Teulade-Fichou and Anton Granzhan

Beilstein J. Org. Chem. **2019,** *15,* 1872–1889. doi:10.3762/bjoc.15.183

Experimental details and supplementary Tables S1 and S2

License and Terms: This is a supporting information file under the terms of the Creative Commons Attribution License (http://creativecommons.org/licenses/by/4.0). Please note that the reuse, redistribution and reproduction in particular requires that the authors and source are credited.
The license is subject to the *Beilstein Journal of Organic Chemistry* terms and conditions: (https://www.beilstein-journals.org/bjoc)

EXPERIMENTAL SECTION

Synthesis of dyes

General remarks. All commercially available chemicals were reagent grade and used without further purification. NMR spectra were recorded with a Bruker Avance 300 spectrometer (^{1}H: 300 MHz, ^{13}C: 75 MHz) at 25 °C; chemical shifts are given in ppm (δ) values and calibrated with respect to the signal of the solvent (DMSO: δ_H = 2.50, δ_C = 39.52 ppm) or MeOH (in D_2O, δ_H = 3.34, δ_C = 49.50 ppm). Multiplicities of ^{13}C NMR signals were determined from DEPT135 or APT experiments. The melting points were determined on a Kofler bench (Wagner & Munz). Elemental microanalysis of all novel compounds was performed by *Service de Microanalyse*, CNRS–ICSN, Gif-sur-Yvette, France. The purity of final compounds was assessed by LC–MS analysis (Waters Alliance 2695 equipped with a Waters XBridge C_{18}-3.5 µm column and a photodiode array detector; eluent A: water with 0.05% TFA, eluent B: MeCN with 0.05% TFA, gradient elution with 2 to 100% of eluent B, flow rate: 0.8 mL min^{-1}). Mass spectra (MS, ESI in the positive-ion mode) were recorded with a Waters ZQ instrument. In the assignment of mass spectra of salts, *M* refers to the organic cation or dication.

Intermediates. Precursor **I17** was purchased from Sigma-Aldrich. Intermediates **I1** [1], **I2** [2], **I7** [1], **I10** [3] and **I18** [1] were prepared as described in the literature. The synthesis of other precursors is described below.

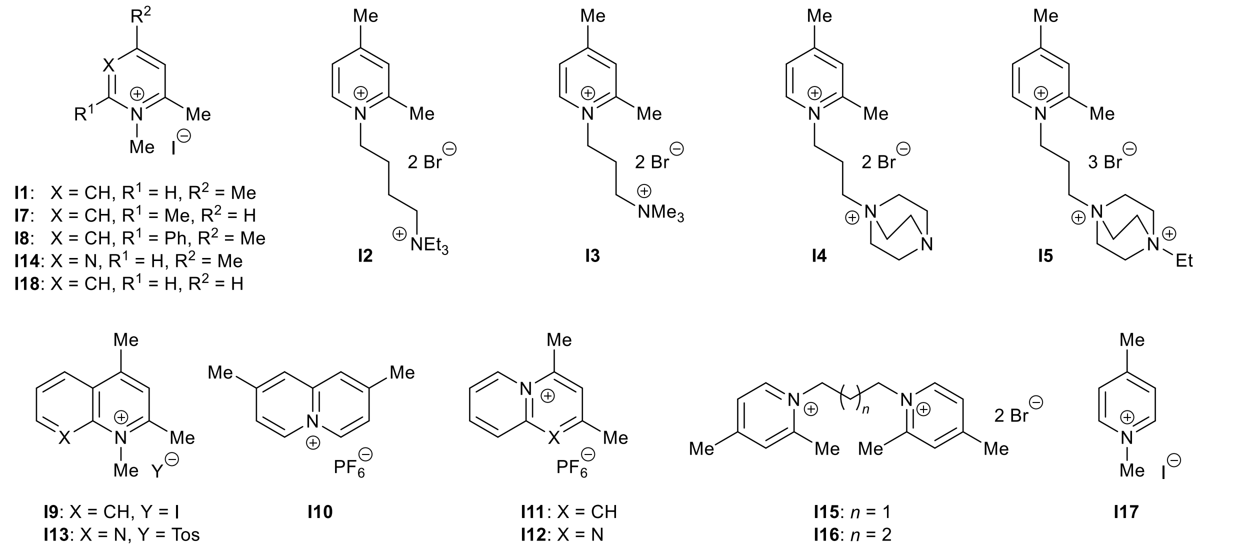

2,4-Dimethyl-1-(3-trimethylammoniopropyl)pyridinium dibromide (I3): A solution of (3-bromopropyl)trimethylammonium bromide (2.26 g, 8.65 mmol) in MeCN (25 mL) was brought to reflux, and 2,4-lutidine (1.00 mL, 0.93 g, 8.65 mmol) was added. After heating at reflux for 24 h, the reaction mixture was cooled and the solvent was evaporated to a half of the initial volume under vacuum. The precipitated solid was collected and washed twice with MeCN and

Et_2O, to yield **I3** (1.50 g, 47%) as a pale-rose solid, m.p. 221–222 °C; ^1H NMR (300 MHz, DMSO): δ 9.04 (d, J = 6.5 Hz, 1H), 7.97 (s, 1H), 7.88 (d, J = 6.4 Hz, 1H), 4.59 (t, J = 7.7 Hz, 2H), 3.52 (dd, J = 9.8, 6.6 Hz, 2H), 3.13 (s, 9H), 2.85 (s, 3H), 2.56 (s, 3H), 2.42–2.21 (m, 2H); ^{13}C NMR (75 MHz, DMSO): δ 158.5 (C_q), 154.2 (C_q), 144.6 (CH), 130.1 (CH), 126.2 (CH), 61.7 (CH_2), 53.1 (CH_2), 52.5 (CH_3), 23.3 (CH_2), 21.2 (CH_3), 19.7 (CH_3); MS (ESI$^+$): 321.2 [M + CF_3COO^-]$^+$, 207.2 [M − H]$^+$, 148.2 [M – NMe_3 − H]$^+$.

1-(3-Bromopropyl)-2,4-dimethylpyridinium bromide: A mixture of 2,4-lutidine (5.4 mL, 5.0 g, 47 mmol), 1,3-dibromopropane (9.5 mL, 18.8 g, 93 mmol) and tetra-*n*-butylammonium iodide (0.43 g, 1.17 mmol) in acetone (50 mL) was heated at reflux for 24 h. After cooling, the suspension was filtered and the precipitate was washed with acetone and dried, to give **I15** (2.0 g, 10%) as a white solid (characterization data below). The filtrate was concentrated under vacuum. The resulting oily residue was triturated several times with a large volume of Et_2O and then dried under vacuum, to give 1-(3-bromopropyl)-2,4-dimethylpyridinium bromide (7.0 g, 49%) as a yellow viscous oil, which was used for the synthesis of **I4** without further purification. ^1H NMR (300 MHz, DMSO): δ 8.85 (d, J = 6.5 Hz, 1H), 7.90 (s, 1H), 7.80 (d, J = 6.3 Hz, 1H), 4.56 (t, J = 7.3 Hz, 2H), 3.46 (t, J = 5.8 Hz, 2H), 2.78 (s, 4H), 2.54 (s, 3H), 1.98 (td, J = 12.6, 6.1 Hz, 2H); ^{13}C NMR (75 MHz, DMSO): δ 158.1 (C_q), 154.0 (C_q), 144.7 (CH), 130.0 (CH), 126.1 (CH), 57.2 (CH_2), 54.2 (CH_2), 32.3 (CH_2), 21.1 (CH_3), 19.4 (CH_3); MS (ESI$^+$): m/z = 228.1 [M]$^+$.

1,1′-(1,3-Propanediyl)-bis(2,4-dimethylpyridinium) dibromide (I15): White solid, m.p. 225–226 °C; ^1H NMR (300 MHz, DMSO): δ 9.03 (d, J = 6.5 Hz, 2H), 7.94 (s, 2H), 7.86 (d, J = 6.4 Hz, 2H), 4.69 (t, J = 7.8 Hz, 4H), 2.85 (s, 6H) 2.55 (s, 6H), 2.50–2.35 (m, 2H); ^{13}C NMR (75 MHz, DMSO): δ 158.5 (C_q), 154.3 (C_q), 144.5 (CH), 130.1 (CH), 126.1 (CH), 53.1 (CH_2), 29.6 (CH_2), 21.2 (CH_3), 19.7 (CH_3); MS (ESI$^+$): m/z = 255.2 [M − H]$^+$, 128.2 [M]$^{2+}$.

1-(3-(4-aza-1-azoniabicyclo[2.2.2]octyl)propyl)-2,4-dimethylpyridinium dibromide (I4): A mixture of 1-(3-bromopropyl)-2,4-dimethylpyridinium dibromide (0.535 g, 1.73 mmol) and 1,4-diazabicyclo[2.2.2]octane (0.388 g, 3.46 mmol) in MeCN (20 mL) was stirred at reflux for 16 h, then cooled to room temperature and poured into Et_2O (200 mL). The precipitate was collected by filtration and washed with Et_2O, to give **I4** (0.30 g, 41%) as a white, very hygroscopic solid; ^1H NMR (300 MHz, D_2O): δ 8.57 (d, J = 6.5 Hz, 1H), 7.76 (s, 1H), 7.72 (d, J = 6.4 Hz, 1H), 4.58 (t, J = 7.9 Hz, 2H), 3.55–3.44 (m, 8H), 3.23 (dt, J = 11.8, 6.1 Hz, 6H), 2.81 (s, 3H), 2.58 (s, 3H), 2.53–2.38 (m, 2H); ^{13}C NMR (75 MHz, D_2O): δ 160.9 (C_q), 154.8 (C_q), 144.2 (CH), 131.3 (CH), 127.4 (CH), 61.0 (CH_2), 53.9 (CH_2), 53.0 (CH_2), 44.8 (CH_2), 23.0 (CH_2), 21.7 (CH_3), 20.0 (CH_3); MS (ESI$^+$): m/z = 260.3 [M – H]$^+$, 148.2 [M – $C_6H_{12}N_2$ – H]$^+$, 130.7 [M]$^{2+}$.

1-(3-(4-ethyl-1,4-diazoniabicyclo[2.2.2]octyl)propyl)-2,4-dimethylpyridinium tribromide (I5): A mixture of **I4** (2.00 g, 4.75 mmol) and ethyl bromide (0.35 mL, 0.52 g, 4.75 mmol) in MeCN (50 mL) was heated at 70 °C for 18 h. After cooling, the precipitated solid was collected, washed with MeCN and Et_2O and dried under vacuum, to give **I5** (0.80 g, 32%) as a white solid, m.p. 216–217 °C; ^1H NMR (300 MHz, D_2O): δ 8.58 (d, J = 6.5 Hz, 1H), 7.77 (s, 1H), 7.72 (d, J = 6.4 Hz, 1H), 4.62 (t, J = 7.9 Hz, 2H), 4.05 (td, J = 13.7, 7.9 Hz, 12H), 3.91 – 3.83 (m, 2H), 3.68 (q, J = 7.3 Hz, 2H), 2.81 (s, 3H), 2.58 (s, 3H), 2.57 – 2.47 (m, 2H), 1.43 (t, J = 7.3 Hz, 3H); ^{13}C NMR (75 MHz, D_2O): δ 161.1 (C_q), 154.8 (C_q), 144.3 (CH), 131.4 (CH), 127.4 (CH), 61.8 (CH_2), 53.4 (CH_2), 52.2 (CH_2), 51.2 (CH_2), 44.8 (CH_2), 23.3 (CH_2), 21.7 (CH_3), 20.0 (CH_3), 7.7 (CH_3); MS (ESI^+): m/z = 288.4 $[M - 2H]^+$, 260.3 $[M - Et - H]^+$, 148.1 $[M - (C_6H_{12}N_2)Et - H]^+$, 130.7 $[M - Et]^{2+}$.

1,2,4-Trimethyl-6-phenylpyridinium iodide (I8): A solution of 2,4-dimethyl-6-phenylpyridine [4] (0.30 g, 1.64 mmol) and iodomethane (1.02 mL, 2.32 g, 16.4 mmol) in acetone (5 mL) was stirred under reflux for 60 h and cooled to room temperature. The precipitated solid was collected, washed twice with acetone and dried under vacuum, to give **I8** (0.15 g, 28%) as a white solid; ^1H NMR (300 MHz, DMSO): δ 7.95 (s, 1H), 7.78 (s, 1H), 7.70–7.57 (m, 5H), 3.88 (s, 3H), 2.81 (s, 3H), 2.57 (s, 3H); ^{13}C NMR (75 MHz, DMSO): δ 157.0 (C_q), 155.4 (C_q), 154.6 (C_q), 132.9 (C_q), 130.8 (CH), 129.1 (CH), 129.0 (CH), 128.5 (CH), 128.0 (CH), 42.1 (CH_3), 21.2 (CH_3), 20.9 (CH_3); MS (ESI^+): m/z = 198.2 $[M]^+$.

1,2,4-Trimethylquinolinium iodide (I9): A solution of 2,4-dimethylquinoline (4.76 mL, 5.00 g, 31.8 mmol) and iodomethane (1.99 mL, 4.51 g, 31.8 mmol) in acetone (50 mL) was stirred at reflux for 18 h and then cooled to room temperature. The precipitated solid was collected, washed twice with acetone and dried under vacuum, to give **I9** (7.30 g, 77%) as a white solid, m.p. 265–266 °C (lit. 254–256 °C [5]); ^1H NMR (300 MHz, DMSO): δ 8.56 (d, J = 9.0 Hz, 1H), 8.45 (dd, J = 8.4, 1.2 Hz, 1H), 8.20 (ddd, J = 8.8, 7.0, 1.4 Hz, 1H), 8.06 (s, 1H), 7.98 (t, J = 7.4 Hz, 1H), 4.40 (s, 3H), 3.03 (s, 3H), 2.93 (s, 3H); ^{13}C NMR (75 MHz, DMSO): δ 159.6 (C_q), 155.9 (C_q), 138.7 (C_q), 134.6 (CH), 128.7 (CH), 127.2 (C_q), 126.7 (CH), 125.6 (CH), 119.3 (CH), 39.4 (CH_3), 22.9 (CH_3), 19.3 (CH_3); MS (ESI^+): m/z = 172.2 $[M]^+$.

2,4-Dimethylquinolizinium hexafluorophosphate (I11): A solution of 2-picoline (4.28 mL, 4.02 g, 43.2 mmol) in dry Et_2O (36 mL) was cooled in an ice bath under argon. PhLi (1.9 M solution in Bu_2O, 23.9 mL, 45.4 mmol) was subsequently added in small portions via a syringe. The dark-yellow solution was stirred in the ice bath for 20 min, then at room temperature for 1 h and finally cooled again in the ice bath. A solution of 1-(2-methyl-1,3-dioxolan-2-yl)propan-2-one [6] (9.34 g, 64.8 mmol) in dry Et_2O (12 mL) was added. The reaction mixture was stirred in the ice bath for 15 min and then at room temperature for 30 min and finally poured into an

ice-water mixture (100 mL). The organic phase was separated and the aqueous layer was extracted with MTBE (3 × 50 mL). The combined organic phases were washed with water and brine, dried over Na_2SO_4. The volatiles were removed in vacuo, yielding a brown residue containing the crude 2-methyl-1-(2-methyl-1,3-dioxolan-2-yl)-3-(pyridin-2-yl)propan-2-ol (4.44 g). The residue was dissolved in Ac_2O (10 mL) and H_2SO_4 (96%, 0.50 mL) was carefully added. The mixture was heated at reflux (bath temp. 150 °C) for 3 h, then cooled to room temperature. Ice-water (20 mL) was subsequently added and the resulting mixture was left to stir overnight. Charcoal (1 g) was then added and the mixture was filtered. The filter cake was then rinsed with water. NH_4PF_6 (6.30 g, 38.6 mmol) dissolved in a small amount of water was added to the filtrate. The precipitated solid was collected by filtration, washed with water (3x), dried under vacuum and recrystallized from EtOH, to give **I11** (1.82 g, 14% yield) as a white crystalline solid. ^{1}H NMR (300 MHz, DMSO): δ 9.16 (d, J = 7.1 Hz, 1H), 8.46 (dd, J = 8.3, 1.3 Hz, 1H), 8.38–8.27 (m, 2H), 8.03 (dd, J = 7.0 Hz, 1H), 7.99 (s, 1H), 2.97 (s, 3H), 2.64 (s, 3H); ^{13}C NMR (75 MHz, DMSO): δ 148.7 (C_q), 144.0 (C_q), 142.8 (C_q), 135.7 (CH), 132.6 (CH), 127.0 (CH), 126.8 (CH), 124.5 (CH), 122.7 (CH), 21.0 (CH_3), 19.8 (CH_3); MS (ESI$^+$): m/z = 158.1 [M^+].

2,4-Dimethyl-1-azaquinolizinium hexafluorophosphate (I12) [7]**:** A mixture of 2-aminopyridine (1.00 g, 10.6 mmol) and 2,4-pentanedione (1.31 mL, 1.28 g, 12.8 mmol) with polyphosphoric acid (10 mL) was stirred at 90 °C for 3 h and then poured into ice (60 g). A solution of NH_4PF_6 (6.93 g, 42.5 mmol) in a minimal volume of water was added to the melt. The resulting precipitate was collected by suction filtration, washed with water, and dried to give **I12** (2.90 g, 90%) as a white solid, m.p. 207–208 °C; ^{1}H NMR (300 MHz, DMSO): δ 9.25 (d, J = 6.9 Hz, 1H), 8.61 (ddd, J = 8.5, 7.1, 1.2 Hz, 1H), 8.44 (dd, J = 8.7, 1.0 Hz, 1H), 8.15–8.07 (m, 2H), 2.99 (s, 3H), 2.83 (s, 3H); ^{13}C NMR (75 MHz, DMSO): δ 170.3 (C_q), 152.3 (C_q), 148.5 (C_q), 141.4 (CH), 133.0 (CH), 127.3 (CH), 123.0 (CH), 120.6 (CH), 24.9 (CH_3), 19.4 (CH_3); MS (ESI$^+$): m/z = 159.2 [M]$^+$.

1,2,4-Trimethyl-1,8-naphthyridinium tosylate (I13): A mixture of 2,4-dimethyl-1,8-naphthyridine [8,9] (0.791 mg, 5 mmol) and methyl *p*-toluenesulfonate (1.39 g, 7.5 mmol) was put under argon atmosphere, immersed into a preheated (120 °C) oil bath, and heated upon stirring for 45 min. After cooling, acetone (20 mL) was added, and the solid was collected by suction filtration, washed with acetone and dried, to give **I13** (0.305 g, 35%) as a pale-rose solid, m.p. (from 2-PrOH) 210–212 °C; ^{1}H NMR (300 MHz, DMSO): δ 9.33 (d, J = 4.2, 1.6 Hz, 1H), 8.98 (dd, J = 8.4, 1.6 Hz, 1H), 8.11 (s, 1H), 8.08 (dd, J = 8.4, 4.3 Hz, 1H), 7.45 (d, J = 8.0 Hz, 2H), 7.09 (d, J = 7.9 Hz, 2H), 4.48 (s, 3H), 3.05 (s, 3H), 2.94 (s, 3H), 2.28 (s, 3H); ^{13}C NMR (75 MHz, DMSO): δ 162.6 (C_q), 157.1 (C_q), 155.5 (CH), 146.5 (C_q), 145.9 (C_q), 137.5 (C_q), 136.9 (CH), 128.0 (CH), 126.0 (CH), 125.4 (CH), 124.7 (CH), 122.4 (C_q), 36.2 (CH_3), 22.7

(CH_3), 20.8 (CH_3), 18.7 (CH_3); MS (ESI$^+$): *m*/*z* = 173.2 [*M*]$^+$. Structure assignment was confirmed by a characteristic NOE signal between the methyl substituents at N(1) (δ_H 4.48 ppm) and C(2) (δ_H 3.05 ppm).

1,4,6-Trimethylpyrimidinium iodide (I14): A mixture of 4,6-dimethylpyrimidine (2.16 g, 20 mmol) and iodomethane (1.88 mL, 4.26 g, 30 mmol) in acetone (20 mL) was stirred under reflux for 18 h and then cooled to room temperature. The precipitated solid was collected, washed twice with acetone and dried under vacuum, to give **I14** (3.82 g, 76%) as a white solid. ^{1}H NMR (300 MHz, DMSO): δ 9.57 (s, 1H), 8.09 (s, 1H), 4.11 (s, 3H), 2.75 (s, 3H), 2.69 (s, 3H); ^{13}C NMR (75 MHz, DMSO): δ 174.4 (C_q), 162.6 (C_q), 153.7 (CH), 123.4 (CH), 41.3 (CH_3), 24.3 (CH_3), 19.6 (CH_3); MS (ESI$^+$): *m*/*z* = 123.2 [*M*]$^+$.

1,1′-(1,4-Butanediyl)-bis(2,4-dimethylpyridinium) dibromide (I16): A mixture of 2,4-lutidine (5.4 mL, 5.0 g, 47 mmol), 1,4-dibromobutane (2.8 mL, 5.0 g, 23.5 mmol) and tetra-*n*-butylammonium iodide (0.43 g, 1.17 mmol) in acetone (50 mL) was heated under reflux for 24 h. After cooling, the suspension was filtered and the resulting precipitate was washed with acetone and dried, to give **I16** (2.80 g, 28%) as a white solid; ^{1}H NMR (300 MHz, DMSO): δ 8.94 (d, *J* = 6.4 Hz, 2H), 7.93 (s, 2H), 7.82 (dd, *J* = 6.4, 1.4 Hz, 2H), 4.59 (br s, 4H), 2.82 (s, 6H) 2.54 (s, 6H), 1.95 (br s, 4H); ^{13}C NMR (75 MHz, DMSO): δ 158.2 (C_q), 153.9 (C_q), 144.4 (CH), 130.1 (CH), 126.1 (CH), 55.7 (CH_2), 26.0 (CH_2), 21.1 (CH_3), 19.5 (CH_3); MS (ESI$^+$): *m*/*z* = 269.2 [*M* − H]$^+$, 162.2 [*M* – C_7H_9N – H]$^+$, 135.2 [*M*]$^{2+}$.

Synthesis of dyes. Dyes **1a**, **7a**, **17a** and **18a** were prepared according to the published procedures and gave satisfactory ^{1}H and ^{13}C NMR, MS, and elemental analysis data [1]. The synthesis of the dyes **1y** (**BCVP**) [10] and **2a** [2] was described elsewhere. The purity of all dyes was confirmed by the HPLC analysis.

General procedure for the synthesis of distyryl dyes by Knoevenagel condensation[1]: A mixture of the heterocyclic salt **I1–I16** (2.5 mmol), aldehyde (7.5 mmol, 3 molar equiv, unless otherwise stated) and piperidine (0.50 mL, 5 mmol, 2 molar equiv) in EtOH (25 mL) was heated under reflux for 2.5 h. After cooling to room temperature, the precipitated solid was collected by filtration, washed with EtOH (2 × 5 mL) and Et_2O (2 × 5 mL) and dried. The crude iodide salt was either purified through a recrystallization from a suitable solvent (as indicated below) to give an analytically pure sample, or subjected to anion exchange to bromide or chloride, as described below.

[1] Except for the dye **6a** whose preparation is detailed below.

Procedure for anion exchange: Ion-exchange resin (Amberlite IRA-402, Cl^- form, or Amberlite IRA-400, Br^- form, about 20 mmol equiv) was thoroughly rinsed with a mixture of MeCN and MeOH (1:1, v/v) and charged into a short glass column. The dye (iodide salt) was dissolved in a minimal amount of a mixture of MeCN and MeOH (1:1, v/v) and loaded in the column. The product was then eluted with the same solvent mixture (about 50 mL). The solvents were removed in vacuo and the residue was recrystallized from a suitable solvent (as indicated below), to give an analytically pure dye.

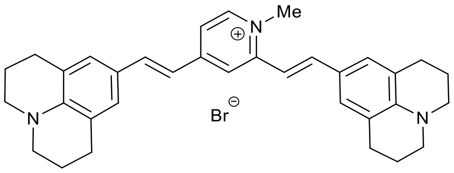

2,4-Bis((*E*)-2-(julolidin-9-yl)vinyl]-1-methylpyridinium bromide (1b): Prepared in 62% yield from **I1** and julolidine-9-carbaldehyde, followed by ion exchange to prepare the bromide salt and recrystallization from MeOH. Black solid, m.p. 247–248 °C; ^{1}H NMR (300 MHz, DMSO): *δ* 8.43 (d, *J* = 6.8 Hz, 1H), 8.25 (s, 1H), 7.75 (d, *J* = 15.7 Hz, 2H), 7.62 (d, *J* = 6.1 Hz, 1H), 7.24 (s, 2H), 7.12 (s, 2H), 7.02 (d, *J* = 15.3 Hz, 1H), 6.98 (d, *J* = 15.7 Hz, 1H), 4.10 (s, 3H), 3.29–3.07 (m, 8H), 2.87–2.60 (m, 8H), 2.22–1.68 (m, 8H); ^{13}C NMR (75 MHz, DMSO): *δ* 152.1 (C_q), 151.6 (C_q), 145.1 (C_q), 144.8 (C_q), 144.1 (CH), 143.0 (CH), 140.5 (CH), 128.0 (CH), 127.3 (CH), 121.7 (C_q), 121.4 (C_q), 120.7 (C_q), 120.6 (C_q), 117.8 (CH), 117.5 (CH), 116.6 (CH), 109.6 (CH), 49.3 (CH_2), 44.1 (CH_3), 27.1 (CH_2), 21.0 (CH_2); MS (ESI$^+$): *m*/*z* = 488.6 [*M*]$^+$; anal. calcd. for $C_{34}H_{38}BrN_3$ × 0.5 H_2O (577.6): C 70.7, H 6.81, N 7.27; found: C 70.79, H 6.42, N 7.01.

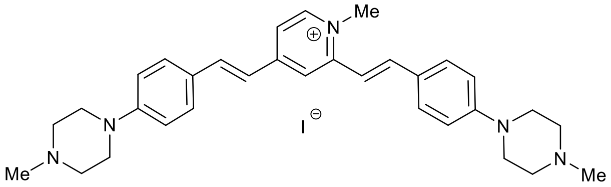

1-Methyl-2,4-bis((*E*)-4-(4-methylpiperazin-1-yl)styryl)pyridinium iodide (1c): Prepared in 47% yield from **I1** and 4-(4-methylpiperazin-1-yl)benzaldehyde, followed by recrystallization from EtOH. Cherry-red solid, m.p. 228–229 °C; ^{1}H NMR (300 MHz, DMSO): *δ* 8.59 (d, *J* = 6.7 Hz, 1H), 8.42 (s, 1H), 7.94–7.81 (m, 3H), 7.72 (d, *J* = 8.5 Hz, 2H), 7.59 (d, *J* = 8.4 Hz, 2H), 7.27 (d, *J* = 16.2 Hz, 1H), 7.20 (d, *J* = 16.6 Hz, 1H) 7.03 (d, *J* = 7.2, 4H), 4.19 (s, 3H), 3.38–3.25 (m, 8H), 2.48–2.39 (m, 8H), 2.23 (s, 6H); ^{13}C NMR (75 MHz, DMSO): *δ* 152.4 (C_q), 152.2 (C_q), 152.1 (C_q), 151.9 (C_q), 144.7 (CH), 142.4 (CH), 140.0 (CH), 130.2 (CH), 129.5 (CH), 124.9 (C_q), 124.6 (C_q), 119.3 (CH), 119.0 (CH), 114.5 (CH), 114.3 (CH), 112.7 (CH), 54.4 (2 CH_2), 46.8 (CH_2), 46.7 (CH_2), 45.7 (2 CH_3), 44.5 (CH_3); MS (ESI$^+$): *m*/*z* = 494.6 [*M*]$^+$, 247.9 [*M*

+ H]$^+$, 165.7 [M + 2H]$^{3+}$; anal. calcd. for $C_{32}H_{40}IN_5$ × 0.5 H_2O (630.6): C 60.95, H 6.55, N 11.11; found: C 60.91, H 6.42, N 11.40.

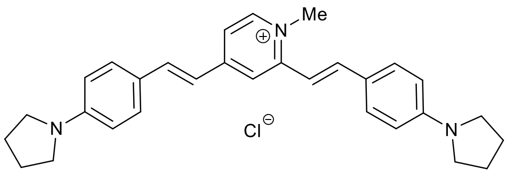

1-Methyl-2,4-bis((*E*)-4-(pyrrolidin-1-yl)styryl)pyridinium chloride (1d): Prepared in 65% yield from **I1** and 4-(pyrrolidin-1-yl)benzaldehyde, followed by ion exchange to prepare the chloride salt. Shiny black crystals, m.p. 248–249 °C; ^{1}H NMR (300 MHz, DMSO): δ 8.49 (d, J = 6.8 Hz, 1H), 8.32 (s, 1H), 7.87 (d, J = 16.1 Hz, 1H), 7.86 (d, J = 15.7 Hz, 1H), 7.75–7.66 (m, 3H), 7.57 (d, J = 8.7 Hz, 2H), 7.14 (d, J = 15.7 Hz, 1H), 7.08 (d, J = 16.1 Hz, 1H), 6.68–6.61 (m, 4H), 4.14 (s, 3H), 3.49–3.45 (m, 8H), 2.24–1.83 (m, 8H); ^{13}C NMR (75 MHz, DMSO): δ 152.2 (C_q), 151.8 (C_q), 149.3 (C_q), 149.1 (C_q), 144.2 (CH), 142.9 (CH), 140.5 (CH), 130.5 (CH), 129.9 (CH), 122.2 (C_q), 121.9 (C_q), 118.4 (CH), 117.9 (CH), 116.9 (CH), 112.0 (CH), 111.8 (CH), 110.5 (CH), 47.3 (CH_2), 44.2 (CH_3), 24.9 (CH_2); MS (ESI$^+$): m/z = 436.3 [M]$^+$; anal. calcd. for $C_{30}H_{34}ClN_3$ × 1.5 H_2O (499.1): C 72.20, H 7.47, N 8.42; found: C 72.53, H 7.43, N 8.24.

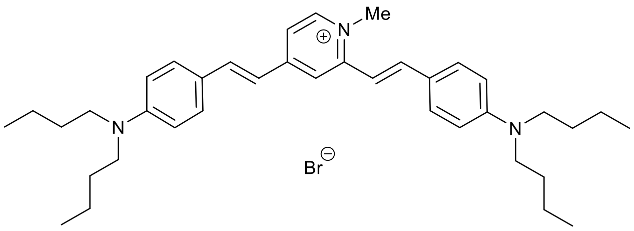

2,4-Bis((*E*)-4-(dibutylamino)styryl)-1-methylpyridinium bromide (1ð): Prepared in 13% yield from **I1** and 4-(dibutylamino)benzaldehyde, followed by ion exchange to prepare the bromide salt and recrystallization from MeCN. Brick-red solid, m.p. 246–247 °C; ^{1}H NMR (300 MHz, DMSO): δ 8.49 (d, J = 6.9 Hz, 1H), 8.32 (s, 1H), 7.86 (d, J = 15.8 Hz, 2H), 7.74 (d, J = 6.0 Hz, 1H), 7.66 (d, J = 8.7 Hz, 2H), 7.54 (d, J = 8.7 Hz, 2H), 7.11 (d, J = 15.4 Hz, 1H), 7.07 (d, J = 15.8 Hz, 1H), 6.72 (d, J = 8.2 Hz, 4H), 4.13 (s, 3H), 3.44–3.30 (m, 8H), 1.62–1.44 (m, 8H), 1.44–1.23 (m, 8H), 0.93 (t, J = 7.2 Hz, 12H); ^{13}C NMR (75 MHz, DMSO): δ 152.2 (C_q), 151.8 (C_q), 149.8 (C_q), 149.5 (C_q), 144.2 (CH), 142.7 (CH), 140.4 (CH), 130.7 (CH), 130.0 (CH), 121.9 (C_q), 121.7 (C_q), 118.5 (CH), 117.9 (CH), 117.1 (CH), 111.5 (CH), 111.3 (CH), 110.5 (CH), 49.8 (CH_2), 44.2 (CH_3), 29.0 (CH_2), 19.6 (CH_2), 13.9 (CH_3); MS (ESI$^+$): m/z = 552.0 [M]$^+$; anal. calcd. for $C_{38}H_{54}BrN_3$ × 1.2 H_2O (654.4): C 69.75, H 8.69, N 6.42; found: C 69.62, H 8.56, N 6.36.

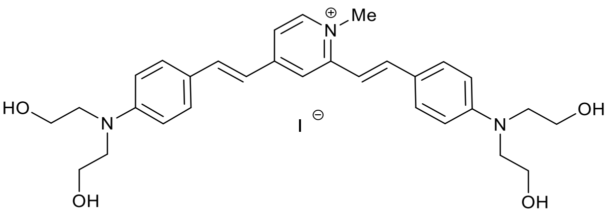

2,4-Bis((*E*)-4-(bis(2-hydroxyethyl)amino)styryl)-1-methylpyridinium iodide (1e): Prepared in 53% yield from **I1** and 4-[bis(2-hydroxyethyl)amino]benzaldehyde, followed by recrystallization from $CHCl_3$–EtOH. Shiny crimson crystals, m.p. 150–151 °C; ^1H NMR (300 MHz, DMSO): δ 8.50 (d, J = 6.8 Hz, 1H), 8.33 (s, 1H), 7.91–7.82 (m, 2H), 7.74 (d, J = 6.6 Hz, 1H), 7.66 (d, J = 8.7 Hz, 2H), 7.54 (d, J = 8.7 Hz, 2H), 7.14 (d, J = 15.7 Hz, 1H), 7.09 (d, J = 16.1 Hz, 1H), 6.80 (dd, J = 8.8 Hz, 4H), 4.84–4.79 (m, 4H, *OH*), 4.14 (s, 3H), 3.64–3.47 (m, 16H); ^{13}C NMR (75 MHz, DMSO): δ 152.2 (C_q), 151.9 (C_q), 150.3 (C_q), 150.0 (C_q), 144.3 (CH), 142.7 (CH), 130.5 (CH), 129.9 (CH), 122.2 (C_q), 122.1 (C_q), 118.5 (CH), 118.1 (CH), 117.3 (CH), 111.7 (CH), 111.5 (CH), 110.8 (CH), 58.1 (CH_2), 53.1 (CH_2), 44.3 (CH_3); MS (ESI$^+$): m/z = 504.6 $[M]^+$, 252.8 $[M + H]^{2+}$, 243.9 $[M - H_2O + H]^{2+}$; anal. calcd. for $C_{30}H_{38}IN_3O_4 \times H_2O$ (649.6): C 55.47, H 6.21, N 6.47; found: C 55.70, H 6.51, N 6.19.

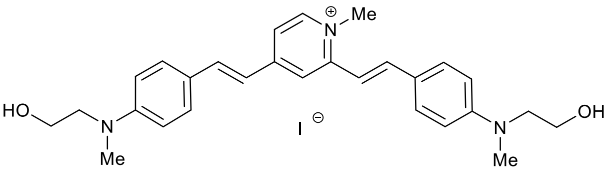

2,4-Bis((*E*)-4-((2-hydroxyethyl)(methyl)amino)styryl)-1-methylpyridinium iodide (1f): Prepared in 14% yield from **I1** and *N*-methyl-*N*-(2-hydroxyethyl)-4-aminobenzaldehyde, followed by recrystallization from MeCN–MeOH. Shiny black solid, m.p. 239–240 °C; ^1H NMR (300 MHz, DMSO): δ 8.50 (d, J = 6.7 Hz, 1H), 8.35 (s, 1H), 7.88 (d, J = 15.9 Hz, 2H), 7.74 (d, J = 6.1 Hz, 1H), 7.68 (d, J = 8.5 Hz, 2H), 7.56 (d, J = 8.4 Hz, 2H), 7.14 (d, J = 15.4 Hz, 1H), 7.10 (d, J = 15.8 Hz, 1H), 6.79 (d, J = 7.8 Hz, 4H), 4.83–4.66 (m, 2H, *OH*), 4.15 (s, 3H), 3.66–3.54 (m, 4H), 3.54–3.44 (m, 4H), 3.04 (s, 3H), 3.03 (s, 3H); ^{13}C NMR (75 MHz, DMSO): δ 152.2 (C_q), 151.9 (C_q), 151.1 (C_q), 150.8 (C_q), 144.3 (CH), 142.8 (CH), 140.4 (CH), 130.5 (CH), 129.8 (CH), 122.4 (C_q), 122.2 (C_q), 118.6 (CH), 118.1 (CH), 117.4 (CH), 111.7 (CH), 111.6 (CH), 110.8 (CH), 58.2 (CH_2), 53.9 (CH_2), 44.3 (CH_3), 38.8 (2 CH_3); MS (ESI$^+$): m/z = 444.4 $[M]^+$, 222.8 $[M + H]^{2+}$, 213.8 $[M - H_2O + H]^{2+}$; anal. calcd. for $C_{28}H_{34}IN_3O_2$ (571.5): C 58.85, H 6.00, N 7.35; found: C 58.53, H 5.98, N 7.55.

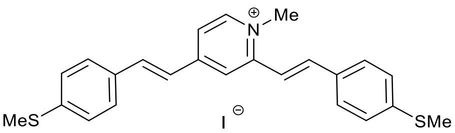

1-Methyl-2,4-bis((*E*)-4-(methylthio)styryl)pyridinium iodide (1g): Prepared in 20% yield from **I1** and 4-(methylthio)benzaldehyde, followed by recrystallization from MeCN–H_2O. Dark-

yellow crystals, m.p. 292–293 °C; ^{1}H NMR (300 MHz, DMSO): δ 8.76 (d, J = 6.7 Hz, 1H), 8.57 (s, 1H), 8.06–7.96 (m, 2H), 7.92 (d, J = 15.8 Hz, 1H), 7.82 (d, J = 8.4 Hz, 2H), 7.69 (d, J = 8.4 Hz, 2H), 7.54 (d, J = 16.0 Hz, 1H), 7.47–7.34 (m, 5H), 4.27 (s, 3H), 2.55 (s, 3H), 2.54 (s, 3H); ^{13}C NMR (75 MHz, DMSO): δ 152.0 (C_q), 151.9 (C_q), 145.5 (CH), 142.2 (C_q), 141.8 (CH), 141.6 (C_q), 139.5 (CH), 131.6 (C_q), 131.3 (C_q), 128.9 (CH), 128.4 (CH), 125.8 (CH), 125.6 (CH), 122.4 (CH), 120.7 (CH), 116.6 (CH), 45.0 (CH_3), 14.2 (2 CH_3); MS (ESI$^+$): m/z = 390.3 [M]$^+$; anal. calcd. for $C_{24}H_{24}INS_2$ (517.5): C 55.70, H 4.67, N 2.71; found: C 55.74, H 4.70, N 2.76.

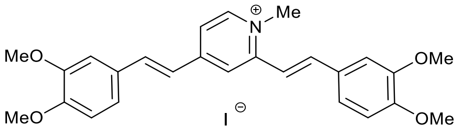

2,4-Bis((*E*)-3,4-dimethoxystyryl)-1-methylpyridin-1-ium iodide (1h): Prepared in 63% yield from **I1** and 3,4-dimethoxybenzaldehyde, followed by recrystallization from MeCN–H_2O. Yellow solid, m.p. > 280 °C; ^{1}H NMR (300 MHz, DMSO): δ 8.72 (d, J = 6.7 Hz, 1H), 8.52 (s, 1H), 8.00 (d, J = 16.3 Hz, 1H), 7.97–7.90 (m, 2H), 7.51–7.35 (m, 5H), 7.30 (dd, J = 8.3, 1.6 Hz, 1H), 7.10 (d, J = 8.4 Hz, 1H), 7.08 (d, J = 8.4 Hz, 1H), 4.26 (s, 3H), 3.88 (s, 3H), 3.86 (s, 3H), 3.84 (s, 3H), 3.83 (s, 3H); ^{13}C NMR (75 MHz, DMSO): δ 152.2 (C_q), 152.0 (C_q), 151.3 (C_q), 151.0 (C_q), 149.1 (C_q), 149.1 (C_q), 145.3 (CH), 142.5 (CH), 140.1 (CH), 128.2 (C_q), 127.9 (C_q), 123.4 (CH), 122.8 (CH), 121.1 (CH), 120.2 (CH), 120.1 (CH), 115.2 (CH), 111.8 (CH), 111.7 (CH), 110.6 (CH), 109.7 (CH), 55.9 (CH_3), 55.7 (CH_3), 55.65 (CH_3), 55.6 (CH_3), 44.9 (CH_3); MS (ESI$^+$): m/z = 418.4 [M]$^+$; anal. calcd. for $C_{26}H_{28}NIO_4 \times 0.5\ H_2O$ (554.4): C 56.33, H 5.27, N 2.53; found: C 56.45, H 5.21, N 2.43.

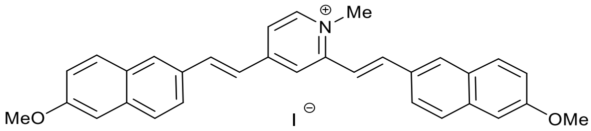

2,4-Bis((*E*)-2-(6-methoxynaphthalen-2-yl)vinyl)-1-methylpyridinium iodide (1i): Prepared in 39% yield from **I1** and 6-methoxy-2-naphthaldehyde, followed by recrystallization from MeOH–$MeNO_2$. Yellow microcrystalline solid, m.p. > 280 °C; ^{1}H NMR (300 MHz, DMSO): δ 8.78 (d, J = 6.8 Hz, 1H), 8.65 (s, 1H), 8.28–8.04 (m, 5H), 8.01 (d, J = 5.5 Hz, 1H), 7.98–7.85 (m, 5H), 7.63 (d, J = 15.9 Hz, 1H), 7.55 (d, J = 16.3 Hz, 1H), 7.40 (dd, J = 8.0, 2.2 Hz, 2H), 7.29–7.18 (m, 2H), 4.31 (s, 3H), 3.92 (s, 3H), 3.91 (s, 3H); ^{13}C NMR (75 MHz, DMSO): δ 158.7 (C_q), 158.6 (C_q), 152.0 (C_q), 151.9 (C_q), 145.5 (CH), 142.5 (CH), 140.2 (CH), 135.6 (C_q), 135.3 (C_q), 130.7 (C_q), 130.5 (C_q), 130.3 (CH), 130.2 (CH), 130.1 (CH), 129.5 (CH), 128.3 (C_q), 128.2 (C_q), 127.7 (CH), 127.6 (CH), 124.7 (CH), 124.1 (CH), 122.7 (CH), 120.8 (CH), 120.6 (CH), 119.5 (CH), 119.4 (CH), 116.8 (CH), 106.4 (CH), 55.4 (2 CH_3), 45.1 (CH_3); MS (ESI$^+$): m/z = 458.3 [M]$^+$; anal. calcd. for $C_{32}H_{28}INO_2$ (585.5): C 65.65, H 4.82, N 2.39; found: C 65.37, H 4.98, N 2.38.

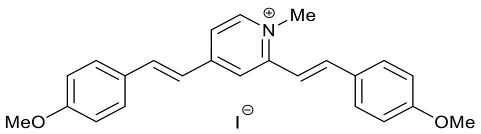

2,4-Bis((*E*)-4-methoxystyryl)-1-methylpyridinium iodide (1j): Prepared in 77% yield from **I1** and 4-methoxybenzaldehyde, followed by recrystallization from MeCN–H_2O. Lemon-yellow needles, m.p. 278–279 °C; ^{1}H NMR (300 MHz, DMSO): δ 8.72 (d, J = 6.7 Hz, 1H), 8.52 (s, 1H), 8.09–7.88 (m, 3H), 7.84 (d, J = 8.8 Hz, 2H), 7.71 (d, J = 8.7 Hz, 2H), 7.42 (d, J = 15.9 Hz, 1H), 7.33 (d, J = 16.3 Hz, 1H), 7.17–6.98 (m, 4H), 4.25 (s, 3H), 3.85 (s, 3H), 3.83 (s, 3H); ^{13}C NMR (75 MHz, DMSO): δ 161.4 (C_q), 161.1 (C_q), 152.2 (C_q), 152.1 (C_q), 145.3 (CH), 142.1 (CH), 139.8 (CH), 130.4 (CH), 129.7 (CH), 127.9 (C_q), 127.7 (C_q), 121.0 (CH), 120.2 (CH), 115.2 (CH), 114.7 (CH), 114.6 (CH), 55.5 (CH_3), 55.4 (CH_3), 44.9 (CH_3); MS (ESI$^+$): m/z = 358.3 [M]$^+$; anal. calcd. for $C_{24}H_{24}INO_2$ (485.4): C 59.39, H 4.98, N 2.89; found: C 59.41, H 4.97, N 2.93.

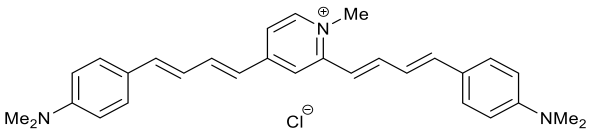

2,4-Bis((1*E*,3*E*)-4-(4-(dimethylamino)phenyl)buta-1,3-dien-1-yl)-1-methylpyridinium chloride (1k): Prepared in 42% yield through the reaction of **I1** with 4-(dimethylamino) cinnamaldehyde in a mixture EtOH (30 mL) and $CHCl_3$ (5 mL) as described above, followed by ion exchange to prepare the chloride salt and recrystallization from MeCN–EtOH. Black metal-shiny solid, m.p. 233–234 °C; ^{1}H NMR (300 MHz, DMSO): δ 8.56 (d, J = 6.8 Hz, 1H), 8.31 (s, 1H), 7.90–7.76 (m, 2H), 7.73 (d, J = 6.8 Hz, 1H), 7.47 (d, J = 8.8 Hz, 4H), 7.18–6.96 (m, 4H), 6.88 (d, J = 15.0 Hz, 1H), 6.75 (d, J = 8.8 Hz, 2H), 6.73 (d, J = 8.8 Hz, 2H), 6.67 (d, J = 15.4 Hz, 1H), 4.09 (s, 3H), 2.99 (s, 6H), 2.98 (s, 6H); ^{13}C NMR (75 MHz, DMSO): δ 151.6 (C_q), 151.4 (C_q), 151.1 (C_q), 151.0 (C_q), 144.6 (CH), 144.1 (CH), 142.2 (CH), 142.0 (CH), 141.2 (CH), 129.0 (CH), 129.0 (CH), 123.7 (C_q), 123.7 (CH), 123.5 (C_q), 123.3 (CH), 123.2 (CH), 119.0 (CH), 118.8 (CH), 117.2 (CH), 112.1 (CH), 112.0 (CH), 44.2 (CH_3), 39.8 (CH_3); MS (ESI$^+$): m/z = 436.5 [M]$^+$; anal. calcd. for $C_{30}H_{34}ClN_3 \times 0.5\ H_2O$ (481.1): C 74.90, H 7.33, N 8.73; found: C 75.20, H 7.31, N 8.65.

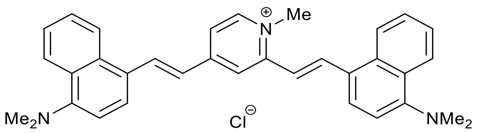

2,4-Bis((*E*)-2-(4-(dimethylamino)naphtalen-1-yl)vinyl)-1-methylpyridinium chloride (1l): Prepared in 50% yield from **I1** and 4-dimethylamino-1-naphthaldehyde, followed by ion exchange to prepare the chloride salt and recrystallization from EtOAc–MeOH. Black microcrystalline solid, m.p. 224–225 °C; ^{1}H NMR (300 MHz, DMSO): δ 8.88 (s, 1H), 8.85–8.70 (m, 3H), 8.61 (t, J = 8.3 Hz, 2H), 8.30 (dd, J = 6.7, 1.3 Hz, 1H), 8.25–8.14 (m, 3H), 8.05 (d, J

= 8.1 Hz, 1H), 7.71–7.59 (m, 4H), 7.54 (d, *J* = 15.6 Hz, 1H), 7.52 (d, *J* = 16.0 Hz, 1H), 7.21 (d, *J* = 8.1Hz, 1H), 7.20 (d, *J* = 8.1Hz, 1H), 4.32 (s, 3H), 2.94 (s, 6H), 2.92 (s, 6H); ^{13}C NMR (75 MHz, DMSO): δ 153.2 (C_q), 152.8 (C_q), 151.9 (C_q), 151.8 (C_q), 145.0 (CH), 138.7 (CH), 136.3 (CH), 132.5 (C_q), 127.5 (C_q), 127.4 (C_q), 126.9 (CH), 126.7 (CH), 126.4 (CH), 126.1 (C_q), 125.7 (C_q), 125.4 (2 CH), 124.9 (CH), 124.8 (CH), 124.3 (CH), 124.2 (CH), 123.7 (CH), 121.8 (CH), 120.2 (CH), 117.6 (CH), 113.7 (CH), 113.5 (CH), 44.8 (CH_3), 44.5 (2 CH_3); MS (ESI$^+$): *m*/*z* = 484.6 [*M*]$^+$, 242.8 [*M* + H]$^{2+}$; anal. calcd. for $C_{34}H_{34}ClN_3$ × 2 H_2O (556.1): C 73.43, H 6.89, N 7.56; found: C 73.30, H 6.49, N 7.29.

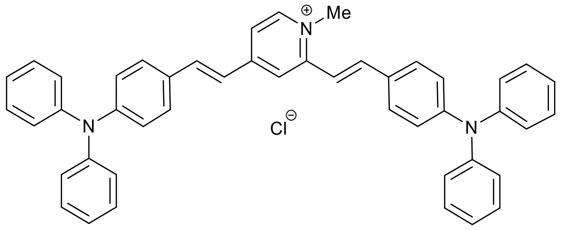

2,4-Bis((*E*)-4-(diphenylamino)styryl)-1-methylpyridinium chloride (1m): Prepared in 10% yield through the reaction of **I1** and 4-(*N*,*N*-diphenylamino)benzaldehyde in a mixture EtOH (25 mL) and $CHCl_3$ (10 mL) as described above, followed by ion exchange to prepare the chloride salt and recrystallization from MeCN–EtOH. Orange-red solid, m.p. 293–294 °C; ^{1}H NMR (300 MHz, DMSO): δ 8.67 (d, *J* = 6.4 Hz, 1H), 8.48 (s, 1H), 8.07–7.82 (m, 3H), 7.73 (d, *J* = 8.2 Hz, 2H), 7.61 (d, *J* = 8.6 Hz, 2H), 7.45–7.28 (m, 10H), 7.27–7.04 (m, 12H), 7.02–6.92 (m, 4H), 4.21 (s, 3H). A ^{13}C NMR spectrum could not be obtained due to insufficient solubility; MS (ESI$^+$): *m*/*z* = 632.4 [*M*]$^+$; anal. calcd. for $C_{46}H_{38}ClN_3$ × 0.2 H_2O (671.9): C 82.23, H 5.76, N 6.25; found: C 82.22, H 5.73, N 6.26.

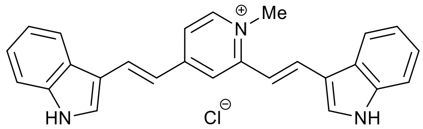

2,4-Bis((*E*)-2-(1*H*-indol-3-yl)vinyl)-1-methylpyridinium chloride (1o): Prepared in 11% yield from **I1** and indole-3-carbaldehyde, followed by ion exchange to prepare the chloride salt and recrystallization from MeCN–EtOH. Brick-red solid, m.p. 310–311 °C; ^{1}H NMR (300 MHz, DMSO): δ 12.17 (br s, 1H), 12.03 (br s, 1H), 8.56 (d, *J* = 6.9 Hz, 1H), 8.51 (s, 1H), 8.29–8.08 (m, 5H), 7.93 (s, 1H), 7.84 (dd, *J* = 6.8, 1.3 Hz, 1H), 7.58–7.48 (m, 2H), 7.36–7.19 (m, 6H), 4.21 (s, 3H); ^{13}C NMR (75 MHz, DMSO): δ 152.7 (C_q), 152.6 (C_q), 144.1 (CH), 137.6 (C_q), 137.4 (C_q), 136.6 (CH), 134.9 (CH), 131.8 (CH), 131.7 (CH), 125.0 (C_q), 124.9 (C_q), 122.9 (CH), 122.8 (CH), 121.1 (CH), 120.9 (CH), 120.5 (CH), 120.2 (CH), 118.1 (CH), 117.5 (CH), 117.3 (CH), 113.5 (C_q), 113.4 (C_q), 112.6 (2 CH), 110.8 (CH), 44.1 (CH_3); MS (ESI$^+$): *m*/*z* = 376.4 [*M*]$^+$; anal. calcd. for $C_{26}H_{22}ClN_3$ × 1.2 H_2O (433.6): C 72.03, H 5.67, N 9.69; found: C 71.99, H 5.70, N 9.60.

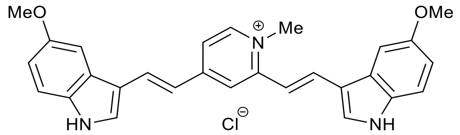

2,4-Bis((*E*)-2-(5-methoxy-1*H*-indol-3-yl)vinyl)-1-methylpyridinium chloride (1p): Prepared in 28% yield from **I1** and 5-methoxyindole-3-carbaldehyde, followed by ion exchange to prepare the chloride salt and recrystallization from MeCN–MeOH. Bright-orange solid, m.p. 230–231 °C; ^{1}H NMR (300 MHz, DMSO): *δ* 11.95 (br s, 1H), 11.83 (br s, 1H), 8.54 (d, *J* = 6.9 Hz, 1H), 8.48 (d, *J* = 1.4 Hz, 1H), 8.25 (d, *J* = 15.8 Hz, 1H), 8.23 (d, *J* = 16.3 Hz, 1H), 8.11 (s, 1H), 7.90 (s, 1H), 7.84 (dd, *J* = 8.4, 1.5 Hz, 1H), 7.63 (d, *J* = 2.2 Hz, 1H), 7.58 (d, *J* = 2.3 Hz, 1H), 7.43 (d, *J* = 8.8 Hz, 1H), 7.42 (d, *J* = 8.8 Hz, 1H), 7.21 (d, J = 16.3 Hz, 1H), 7.18 (d, J = 15.8 Hz, 1H), 6.96–6.85 (m, 2H), 4.21 (s, 3H), 3.89 (s, 3H), 3.88 (s, 3H); ^{13}C NMR (75 MHz, DMSO): *δ* 153.8 (C_q), 153.7 (C_q), 151.5 (C_q), 151.4 (C_q), 142.8 (CH), 135.3 (CH), 133.6 (CH), 131.2 (C_q), 131.0 (C_q), 130.3 (2 CH), 124.8 (C_q), 124.6 (C_q), 116.7 (CH), 116.0 (CH), 115.7 (CH), 112.2 (C_q), 112.1 (CH), 112.0 (CH), 111.1 (CH), 110.9 (CH), 109.1 (CH), 101.9 (CH), 101.3 (CH), 54.6 (CH_3), 54.4 (CH_3), 42.8 (CH_3); MS (ESI$^+$): *m*/*z* = 436.0 [*M*]$^+$; anal. calcd. for $C_{28}H_{26}ClN_3O_2 \times H_2O$ (490.0): C 68.63, H 5.76, N 8.58; found: C 68.89, H 6.02, N 8.76.

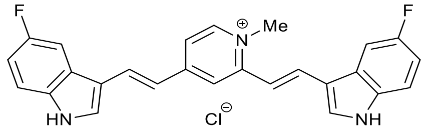

2,4-Bis((*E*)-2-(5-fluoro-1*H*-indol-3-yl)vinyl)-1-methylpyridinium chloride (1q): Prepared in 42% yield from **I1** and 5-fluoroindole-3-carbaldehyde, followed by ion exchange to prepare the chloride salt and recrystallization from MeCN–MeOH. Tangerine solid, m.p. > 310 °C; ^{1}H NMR (300 MHz, DMSO): *δ* 12.30 (br s, 1H), 12.17 (br s, 1H), 8.58 (d, *J* = 6.9 Hz, 1H), 8.51 (d, *J* = 1.2 Hz, 1H), 8.27–8.15 (m, 3H), 8.05–7.91 (m, 3H), 7.86 (dd, *J* = 6.8, 1.4 Hz, 1H), 7.54 (dt, *J* = 8.8, 4.4 Hz, 2H), 7.26 (d, *J* = 16.7 Hz, 1H), 7.20 (d, *J* = 16.1 Hz, 1H), 7.16–7.06 (m, 2 H), 4.22 (s, 3H); ^{13}C NMR (75 MHz, DMSO): *δ* 158.2 (d, J_{CF} = 234 Hz, C_q), 158.1 (d, J_{CF} = 233 Hz, C_q), 152.6 (C_q), 144.2 (CH), 136.1 (CH), 134.3 (CH), 134.1 (C_q), 133.9 (C_q), 132.9 (CH), 132.8 (CH), 125.5 (d, J_{CF} = 10.3 Hz, C_q), 125.2 (d, J_{CF} = 10.3 Hz, C_q), 118.4 (CH), 117.7 (CH), 117.6 (CH), 113.7 (d, J_{CF} = 9.8 Hz, CH), 113.6 (d, J_{CF} = 9.9 Hz, CH), 113.5 (C_q), 113.4 (C_q), 111.1 (CH), 110.8 (d, J_{CF} = 25.8 Hz, CH), 110.7 (d, J_{CF} = 26.0 Hz, CH), 105.5 (d, J_{CF} = 21.3 Hz, CH), 105.2 (d, J_{CF} = 21 Hz, CH), 44.2 (CH_3); MS (ESI$^+$): *m*/*z* = 412.4 [*M*]$^+$; anal. calcd. for $C_{26}H_{20}ClF_2N_3$ (447.9): C 69.72, H 4.50, N 9.38; found: C 69.46, H 4.65, N 9.34.

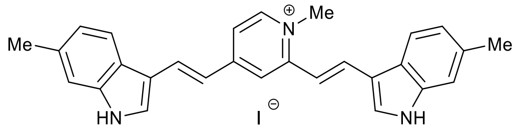

1-Methyl-2,4-bis((*E*)-2-(6-methyl-1*H*-indol-3-yl)vinyl)pyridinium iodide (1r): Prepared in 54% yield from **I1** and 6-methylindole-3-carbaldehyde, followed by recrystallization from

MeCN–MeOH. Orange crystals, m.p. > 310 °C; ^{1}H NMR (300 MHz, DMSO): δ 11.73 (br s, 2H), 8.45 (d, J = 6.9 Hz, 1H), 8.41 (s, 1H), 8.27–8.12 (m, 2H), 8.12–7.94 (m, 3H), 7.85 (s, 1H), 7.76 (d, J = 6.0 Hz, 1H), 7.30 (d, J = 3.3 Hz, 2H), 7.23 (d, J = 16.3 Hz, 1H), 7.16–7.01 (m, 3H), 4.14 (s, 3H), 2.43 (s, 6H); ^{13}C NMR (75 MHz, DMSO): δ 152.5 (C_q), 143.8 (CH), 138.0 (C_q), 137.9 (C_q), 136.7 (CH), 134.8 (CH), 132.2 (C_q), 132.1 (C_q), 131.6 (CH), 131.4 (CH), 122.8 (CH), 122.7 (C_q), 122.6 (CH), 120.3 (CH), 120.0 (CH), 117.9 (CH), 117.3 (CH), 117.0 (CH), 113.6 (C_q), 113.5 (C_q), 112.4 (2 CH), 110.3 (CH), 44.1 (CH_3), 21.3 (2 CH_3); MS (ESI$^+$): m/z = 404.3 [M]$^+$; anal. calcd. for $C_{28}H_{26}IN_3$ × 0.3 CH_3CN × 0.3 H_2O (549.2): C 62.55, H 5.05, N 8.42; found: C 62.50, H 4.97, N 8.63.

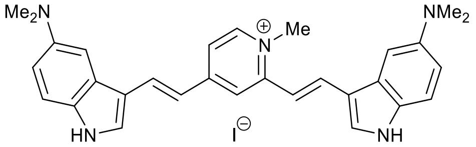

1-Methyl-2,4-bis((*E*)-2-(5-(dimethylamino)-1*H*-indol-3-yl)vinyl)pyridinium iodide (1s): Prepared in 10% yield from **I1** and 6-(dimethyl)indole-3-carbaldehyde [11], followed by recrystallization from MeCN–MeOH. Shiny black crystals, m.p. (decomp.) 146–148 °C; ^{1}H NMR (300 MHz, DMSO): δ 11.73 (s, 1H), 11.61 (s, 1H), 8.48 (d, J = 6.6 Hz, 1H), 8.43 (s, 1H), 8.25 (d, J = 15.3 Hz, 1H), 8.21 (d, J = 15.7 Hz, 1H), 8.03 (s, 1H), 7.84 (s, 1H), 7.79 (d, J = 6.6 Hz, 1H), 7.40–7.28 (m, 4H), 7.13 (d, J = 16.1 Hz, 1H), 7.12 (d, J = 15.3 Hz, 1H), 6.88 (d, J = 8.8 Hz, 2H), 4.17 (s, 3H), 2.97 (s, 12H); ^{13}C NMR (75 MHz, DMSO): δ 152.5 (C_q), 152.4 (C_q), 146.9 (C_q), 143.7 (CH), 136.6 (CH), 135.0 (CH), 130.9 (C_q), 130.7 (CH), 130.6 (C_q), 126.3 (C_q), 126.2 (C_q), 117.3 (CH), 116.8 (CH), 116.3 (CH), 113.1 (C_q), 113.0 (C_q), 112.8 (CH), 112.7 (CH), 111.9 (CH), 109.5 (CH), 103.2 (CH), 102.6 (CH), 43.8 (CH_3), 41.9 (CH_3), 41.6 (CH_3); MS (ESI$^+$): m/z = 462.5 [M]$^+$, 231.8 [M + H]$^+$; anal. calcd. for $C_{30}H_{32}IN_5$ (589.5): C 61.12, H 5.47, N 11.88; found: C 61.73, H 5.87, N 11.63.

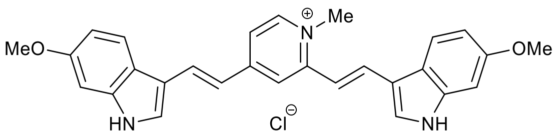

2,4-Bis((*E*)-2-(6-methoxy-1*H*-indol-3-yl)vinyl)-1-methylpyridinium chloride (1t): Prepared in 51% yield from **I1** and 6-methoxyindole-3-carbaldehyde, followed by ion exchange to prepare the chloride salt and recrystallization from MeCN–MeOH. Crimson-red solid, m.p. 305–306 °C; ^{1}H NMR (300 MHz, DMSO): δ 11.87 (s, 1H), 11.75 (s, 1H), 8.53 (d, J = 6.9 Hz, 1H), 8.45 (s, 1H), 8.16 (d, J = 15.7 Hz, 1H), 8.15 (d, J = 16.2 Hz, 1H), 8.05 (d, J = 8.8 Hz, 1H), 8.00 (d, J = 8.8 Hz, 1H), 7.95 (d, J = 2.6 Hz, 1H), 7.82 (d, J = 6.7 Hz, 1H), 7.78 (d, J = 2.6 Hz, 1H), 7.24 (d, J = 16.3 Hz, 1H), 7.15 (d, J = 15.8 Hz, 1H), 7.02 (dd, J = 3.7, 2.3 Hz, 2H), 6.93–6.83 (m, 2H), 4.20 (s, 3H), 3.82 (s, 6H); ^{13}C NMR (75 MHz, DMSO): δ 156.5 (C_q), 156.4 (C_q), 152.7 (C_q), 152.6 (C_q), 144.0 (CH), 138.6 (C_q), 138.5 (C_q), 136.7 (CH), 134.9 (CH), 131.1 (CH),

130.9 (CH), 121.1 (CH), 120.9 (CH), 118.9 (C_q), 118.8 (C_q), 118.0 (CH), 117.2 (CH), 117.1 (CH), 113.7 (C_q), 113.6 (C_q), 111.0 (CH), 110.8 (CH), 110.4 (CH), 95.6 (2 CH), 55.3 (2 CH_3), 44.0 (CH3). MS (ESI$^+$): *m*/*z* = 436.4 [*M*]$^+$; anal. calcd. for $C_{28}H_{26}ClN_3O_2$ × 0.3 H_2O (477.4): C 70.45, H 5.62, N 8.80; found: C 70.70, H 5.48, N 8.92.

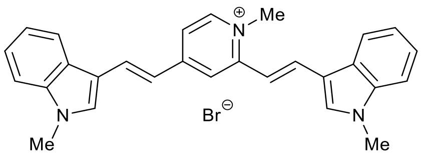

1-Methyl-2,4-bis((*E*)-2-(1-methyl-1*H*-indol-3-yl)vinyl)pyridinium bromide (1u): Prepared in 56% yield from **I1** and 1-methylindole-3-carbaldehyde, followed by ion exchange to prepare the bromide salt and recrystallization from MeCN–EtOH. Shiny cherry red needles, m.p. 290–291 °C; ^{1}H NMR (300 MHz, DMSO): δ 8.52 (d, *J* = 6.9 Hz, 1H), 8.49 (d, *J* = 1.4 Hz, 1H), 8.28–8.13 (m, 4H), 8.12 (s, 1H), 7.91 (s, 1H), 7.83 (dd, *J* = 6.8, 1.6 Hz, 1H), 7.62–7.54 (m, 2H), 7.39–7.23 (m, 5H), 7.18 (d, *J* = 15.8 Hz, 1H), 4.19 (s, 3H), 3.91 (s, 3H), 3.88 (s, 3H); ^{13}C NMR (75 MHz, DMSO): δ 152.4 (C_q), 144.0 (CH), 138.0 (C_q), 137.8 (C_q), 135.9 (CH), 135.0 (2 CH), 134.1 (CH), 125.5 (C_q), 125.3 (C_q), 122.9 (CH), 122.8 (CH), 121.4 (CH), 121.2 (CH), 120.6 (CH), 120.3 (CH), 118.1 (CH), 117.6 (CH), 117.3 (CH), 112.6 (C_q), 112.5 (C_q), 111.0 (CH), 110.9 (CH), 110.7 (CH), 44.1 (CH_3), 33.2 (CH_3), 33.0 (CH_3); MS (ESI$^+$): *m*/*z* = 404.4 [*M*]$^+$; anal. calcd. for $C_{28}H_{26}BrN_3$ × H_2O (502.5): C 66.93, H 5.62, N 8.36; found: C 67.28, H 6.00, N 8.12.

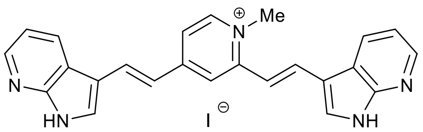

1-Methyl-2,4-bis((*E*)-2-(7-aza-1*H*-indol-3-yl)vinyl)pyridinium iodide (1v): Prepared in 40% yield from **I1** and 7-azaindole-3-carboxaldehyde, followed by recrystallization from MeCN. Dark orange solid, m.p. 294–295 °C; ^{1}H NMR (300 MHz, DMSO): δ 12.42 (s, 2H), 8.68–8.54 (m, 3H), 8.51 (s, 1H), 8.42–8.32 (m, 2H), 8.24 (s, 1H), 8.19 (d, *J* = 16.2 Hz, 2H), 8.05 (s, 1H), 7.87 (d, *J* = 6.1 Hz, 1H), 7.43–7.18 (m, 4H), 4.24 (s, 3H); ^{13}C NMR (75 MHz, DMSO): δ 152.5 (C_q), 149.7 (C_q), 144.4 (CH), 144.2 (CH), 144.1 (CH), 136.1 (CH), 134.3 (CH), 132.1 (CH), 131.7 (C_q), 128.9 (CH), 128.7 (CH), 118.6 (2 CH), 118.0 (CH), 117.3 (C_q), 117.2 (CH), 117.1 (CH), 112.2 (C_q), 112.1 (C_q), 112.0 (CH), 44.4 (CH_3); MS (ESI$^+$): *m*/*z* = 378.4 [*M*]$^+$, 189.8 [*M* + H]$^{2+}$; anal. calcd. for $C_{24}H_{20}IN_5$ × 0.5 H_2O (514.4): C 56.04, H 4.12, N 13.62; found: C 56.28, H 4.12, N 13.98.

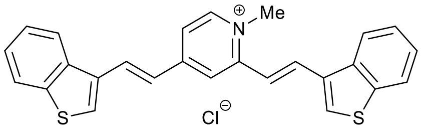

1-Methyl-2,4-bis((*E*)-2-(1-benzothiophen-3-yl)vinyl)pyridinium chloride (1w): Prepared in 20% yield from **I1** and 1-benzothiophene-3-carbaldehyde, followed by ion exchange to

prepare the chloride salt and recrystallization from MeCN–MeOH. Wine-red solid, m.p. 243–244 °C; ^{1}H NMR (300 MHz, DMSO): δ 8.87 (s, 1H), 8.84 (d, J = 6.7 Hz, 1H), 8.67 (s, 1H), 8.51–8.39 (m, 4H), 8.35 (d, J = 16.0 Hz, 1H), 8.21 (d, J = 6.8 Hz, 1H), 8.17–8.09 (m, 2H), 7.71 (d, J = 16.0 Hz, 1H), 7.66–7.46 (m, 5H), 4.34 (s, 3H); ^{13}C NMR (75 MHz, DMSO): δ 152.1 (C_q), 151.9 (C_q), 145.5 (CH), 134.0 (C_q), 139.8 (C_q), 137.2 (C_q), 137.1 (C_q), 133.6 (CH), 132.4 (C_q), 132.1 (C_q), 132.0 (CH), 129.6 (CH), 129.1 (CH), 125.4 (CH), 125.3 (CH), 125.2 (CH), 125.1 (CH), 124.2 (CH), 123.4 (2 CH), 122.7 (CH), 122.5 (CH), 121.4 (CH), 120.9 (CH), 118.6 (CH), 45.1 (CH_3); MS (ESI$^+$): m/z = 410.3 [M]$^+$; anal. calcd. for $C_{26}H_{20}ClNS_2 \times 0.5\ H_2O$ (455.0): C 68.63, H 4.65, N 3.08; found: C 68.69, H 4.58, N 3.27.

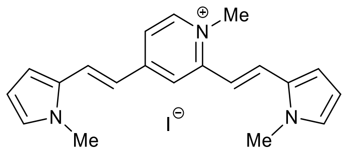

1-Methyl-2,4-bis((*E*)-2-(1-methylpyrrol-2-yl)vinyl)pyridinium iodide (1x): Prepared in 65% yield from **I1** and 1-methylpyrrole-2-carboxaldehyde, followed by recrystallization from MeOH. Shiny green crystals, m.p. 264–266 °C; ^{1}H NMR (300 MHz, DMSO): δ 8.54 (d, J = 6.9 Hz, 1H), 8.40 (d, J = 1.7 Hz, 1H), 7.92 (dd, J = 6.8, 1.8 Hz, 1H), 7.86 (d, J = 16.0 Hz, 1H), 7.83 (d, J = 15.5 Hz, 1H), 7.14–6.99 (m, 5H), 6.82 (dd, J = 3.9, 1.5 Hz, 1H), 6.23 (dd, J = 3.9, 2.6 Hz, 1H), 6.20 (dd, J = 3.9, 2.6 Hz, 1H), 4.14 (s, 3H), 3.84 (s, 3H), 3.81 (s, 3H); ^{13}C NMR (75 MHz, DMSO): δ 152.0 (C_q), 151.8 (C_q), 144.2 (CH), 130.5 (C_q), 130.3 (CH), 128.7 (CH), 128.2 (CH), 128.1 (CH), 119.3 (CH), 118.0 (CH), 117.7 (CH), 112.9 (CH), 111.4 (CH), 111.3 (CH), 109.8 (CH), 109.7 (CH), 44.3 (CH_3), 34.2 (CH_3), 34.1 (CH_3); MS (ESI$^+$): m/z = 304.3 [M]$^+$; anal. calcd. for $C_{20}H_{22}IN_3$ (431.3): C 55.69, H 5.14, N 9.74; found: C 55.67, H 5.21, N 9.82.

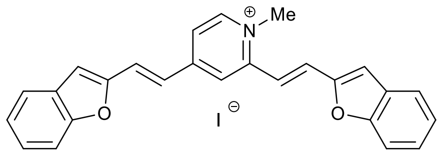

1-Methyl-2,4-bis((*E*)-2-(1-benzofuran-2-yl)vinyl)pyridinium iodide (1z): Prepared in 62% yield from **I1** and 1-benzofuran-2-carbaldehyde, followed by recrystallization from MeCN–H_2O. Dark-yellow crystals, m.p. > 280 °C; ^{1}H NMR (300 MHz, DMSO): δ 8.86 (d, J = 6.7 Hz, 1H), 8.76 (s, 1H), 8.23–8.03 (m, 3H), 7.84–7.73 (m, 2H), 7.68 (d, J = 8.3 Hz, 1H), 7.64 (d, J = 8.3 Hz, 1H), 7.52–7.39 (m, 5H), 7.39–7.27 (m, 3H), 4.32 (s, 3H); ^{13}C NMR (75 MHz, DMSO): δ 155.1 (C_q), 155.0 (C_q), 153.0 (C_q), 152.6 (C_q), 150.9 (C_q), 145.9 (CH), 128.9 (CH), 128.3 (C_q), 128.2 (C_q), 127.2 (CH), 127.1 (CH), 126.9 (CH), 123.9 (CH), 123.8 (CH), 123.7 (CH), 122.4 (CH), 122.3 (CH), 121.7 (CH), 121.4 (CH), 117.9 (CH), 112.4 (CH), 111.6 (CH), 111.4 (CH), 111.2 (CH), 45.1 (CH_3); MS (ESI$^+$): m/z = 378.3 [M]$^+$; anal. calcd. for $C_{26}H_{20}INO_2$ (505.3): C 61.79, H 3.99, N 2.77; found: C 61.72, H 4.13, N 2.77.

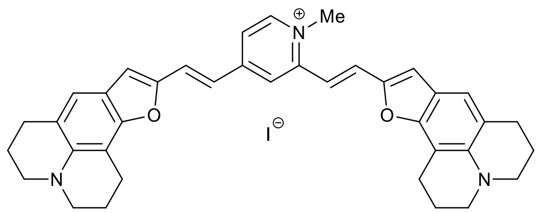

2,4-Bis((*E*)-2-(2,3,6,7-tetrahydro-1*H*,5*H*-furo[2,3-*f*]pyrido[3,2,1-*ij*]quinolin-10-yl)vinyl)-1-methylpyridinium iodide (1Þ): Prepared in 54% yield from **I1** and 2,3,6,7-tetrahydro-1*H*,5*H*-furo[2,3-*f*]-pyrido[3,2,1-*ij*]quinoline-10-carboxaldehyde [12], followed by recrystallization from MeOH–MeCN. Black solid, m.p. 237–238 °C; 1H NMR (300 MHz, DMSO): *δ* 8.54 (d, *J* = 6.8 Hz, 1H), 8.41(s, 1H), 7.99–7.71 (m, 3H), 7.16 (s, 1H), 7.08–6.91 (m, 5H), 4.14 (s, 3H), 3.30–3.10 (m, 8H), 2.96–2.81 (m, 4H), 2.81–2.71 (m, 4H), 2.04–1.80 (m, 8H); ^{13}C NMR (75 MHz, DMSO): *δ* 154.1 (C_q), 153.9 (C_q), 150.9 (C_q), 150.6 (C_q), 150.3 (C_q), 150.0 (C_q), 144.5 (CH), 142.9 (C_q), 142.7 (C_q), 128.6 (CH), 126.7 (CH), 119.8 (C_q), 119.6 (CH + C_q), 119.4 (CH), 118.9 (CH), 118.7 (CH), 118.6 (CH), 117.1 (C_q), 117.0 (C_q), 114.3 (CH), 113.3 (CH), 112.3 (CH), 102.1 (C_q), 102.0 (C_q), 49.5 (CH_2), 49.2 (CH_2), 44.2 (CH_3), 27.7 (CH_2), 21.5 (CH_2), 21.4 (CH_2), 20.3 (CH_2); MS (ESI$^+$): *m*/*z* = 568.5 [*M*]$^+$, 284.5 [*M* + H]$^{2+}$; anal. calcd. for $C_{38}H_{38}IN_3O_2$ × 0.5 H_2O (704.7): C 64.77, H 5.58, N 5.96; found: C 64.68, H 5.48, N 5.72.

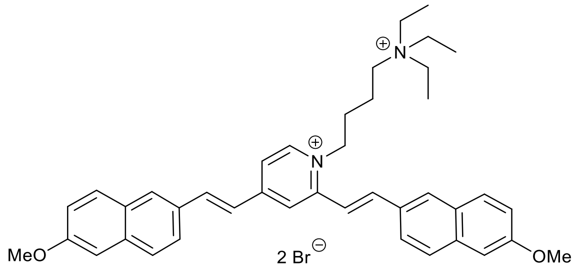

2,4-Bis((*E*)-2-(6-methoxynaphthalen-2-yl)vinyl)-1-(4-(triethylammonio)butyl)pyridinium dibromide (2i): Prepared in 37% yield from **I2** [2] and 6-methoxy-2-naphthaldehyde, followed by recrystallization from EtOH. Dark yellow solid, m.p. 227–228 °C; ^{1}H NMR (300 MHz, DMSO): *δ* 8.92 (d, *J* = 6.7 Hz, 1H), 8.76 (s, 1H), 8.35 (s, 1H), 8.30 (d, *J* = 16.4 Hz, 1H), 8.25–8.07 (m, 4H), 8.06–7.83 (m, 5H), 7.72 (d, *J* = 15.8 Hz, 1H), 7.62 (d, *J* = 16.3 Hz, 1H), 7.43 (dd, *J* = 5.8, 2.3 Hz, 2H), 7.32–7.20 (m, 2H), 4.91–4.76 (m, 2H), 3.93 (s, 3H), 3.92 (s, 3H), 3.30–3.19 (m, 8H), 1.99–1.75 (m, 4H), 1.18 (t, *J* = 6.9 Hz, 9H); ^{13}C NMR (75 MHz, DMSO): *δ* 158.7 (C_q), 158.6 (C_q), 152.3 (C_q), 151.6 (C_q), 145.0 (CH), 143.2 (CH), 140.7 (CH), 135.6 (C_q), 135.4 (C_q), 130.8 (C_q), 130.5 (C_q), 130.3 (CH), 130.2 (CH), 130.1 (CH), 129.6 (CH), 128.3 (C_q), 128.2 (C_q), 127.8 (CH), 127.6 (CH), 125.0 (CH), 124.1 (CH), 122.7 (CH), 121.5 (CH), 121.2 (CH), 119.5 (2 CH), 116.5 (CH), 106.4 (CH), 55.9 (CH_2), 55.4 (2 CH_3), 52.2 (CH_2), 26.7 (CH_2), 18.2 (CH_2), 7.3 (CH_3); MS (ESI$^+$): *m*/*z* = 713.7 [*M* + CF_3COO^-]$^+$, 300.4 [*M*]$^{2+}$; anal. calcd. for $C_{41}H_{48}Br_2N_2O_2$ × 0.5 H_2O (769.7): C 63.98, H 6.42, N 3.64; found: C 63.74, H 6.54, N 3.92.

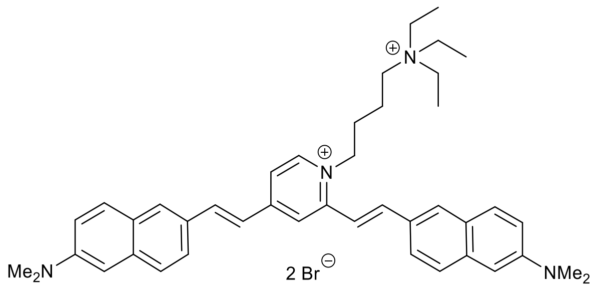

2,4-Bis((*E*)-2-(6-(dimethylamino)naphthalen-2-yl)vinyl)-1-(4-(triethylammonio)butyl)pyridinium dibromide (2n): Prepared in a 54% yield from **I2** and 6-dimethylamino-2-naphthaldehyde, followed by recrystallization from EtOH. Cherry-red solid, m.p. 251–252 °C; ^{1}H NMR (300 MHz, DMSO): δ 8.80 (d, J = 6.7 Hz, 1H), 8.66 (s, 1H), 8.21 (d, J = 16.2 Hz, 1H), 8.18–8.08 (m, 2H), 8.07–7.96 (m, 3H), 7.88–7.71 (m, 5H), 7.57 (d, J = 15.8 Hz, 1H), 7.49 (d, J = 16.3 Hz, 1H), 7.33–7.22 (m, 2H), 7.03–6.96 (m, 2H), 4.77 (t, J = 6.3 Hz, 2H), 3.30–3.17 (m, 8H), 3.08 (s, 6H), 3.07 (s, 6H), 1.97–1.70 (m, 4H), 1.18 (t, J = 6.9 Hz, 9H); ^{13}C NMR (75 MHz, DMSO): δ 152.2 (C_q), 151.5 (C_q), 149.6 (C_q), 149.5 (C_q), 144.6 (CH), 143.5 (CH), 141.0 (CH), 136.0 (C_q), 135.9 (C_q), 130.6 (CH), 130.1 (CH), 129.7 (CH), 129.6 (CH), 128.6 (C_q), 128.4 (C_q), 126.9 (CH), 126.6 (CH), 125.5 (C_q), 125.4 (C_q), 124.7 (CH), 123.8 (CH), 121.0 (CH), 120.8 (CH), 120.2 (CH), 116.5 (CH), 114.5 (CH), 105.5 (CH), 105.4 (CH), 55.6 (CH_2), 55.5 (CH_2), 52.2 (CH_2), 40.0 (CH_3), 26.7 (CH_2), 18.2 (CH_2), 7.3 (CH_3); MS (ESI$^+$): m/z = 713.7 [M + CF_3COO^-]$^+$, 300.4 [M]$^{2+}$; anal. calcd. for $C_{43}H_{54}Br_2N_4$ × 0.5 H_2O (795.7): C 64.90, H 6.97, N 7.04; found: C 64.61, H 7.18, N 7.28.

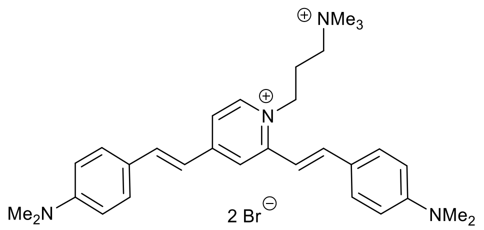

2,4-Bis((*E*)-4-(dimethylamino)styryl)-1-(3-(trimethylammonio)propyl)pyridinium dibromide (3a): Prepared in 51% yield from **I3** and 4-(dimethylamino)benzaldehyde, followed by recrystallization from EtOH. Black solid, m.p. 241–242 °C; ^{1}H NMR (300 MHz, DMSO): δ 8.63 (d, J = 6.9 Hz, 1H), 8.44 (s, 1H), 7.99 (d, J = 16.1 Hz, 1H), 7.98 (d, J = 15.5 Hz, 1H), 7.86 (d, J = 6.8 Hz, 1H), 7.79 (d, J = 8.9 Hz, 2H), 7.61 (d, J = 8.9 Hz, 2H), 7.22 (d, J = 15.5 Hz, 1H), 7.16 (d, J = 16.1 Hz, 1H), 6.80 (d, J = 8.9 Hz, 2H), 6.79 (d, J = 8.9 Hz, 2H), 4.66 (t, J = 7.1 Hz, 2H), 3.63–3.47 (m, 2H), 3.10 (s, 9H), 3.04 (s, 6H), 3.02 (s, 6H), 2.38–2.19 (m, 2H); ^{13}C NMR (75 MHz, DMSO): δ 152.3 (C_q), 152.0 (C_q), 151.8 (C_q), 151.7 (C_q), 143.8 (CH), 143.7 (CH), 141.1 (CH), 130.7 (CH), 129.9 (CH), 122.8 (C_q), 122.5 (C_q), 119.5 (CH), 118.6 (CH), 117.6 (CH), 112.0 (CH), 111.8 (CH), 110.5 (CH), 61.9 (CH_2), 52.5 (CH_2 + CH_3), 39.7 (CH_3), 23.3 (CH_2); MS (ESI$^+$): m/z = 583.6 [M + CF_3COO^-]$^+$, 235.4 [M]$^{2+}$; anal. calcd. for $C_{31}H_{42}Br_2N_4$ × 2 H_2O (666.5): C 55.86, H 6.96, N 8.41; found: C 55.59, H 6.98, N 8.22.

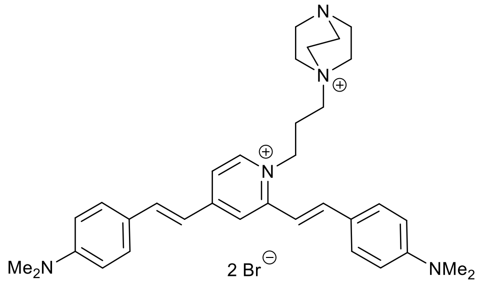

2,4-Bis((*E*)-4-(dimethylamino)styryl)-1-(3-(4-aza-1-azoniabicyclo[2.2.2]octyl)propyl)pyridinium dibromide (4a): Prepared in 62% yield from **I4** and 4-(dimethylamino)benzaldehyde, followed by recrystallization from MeOH. Black solid, m.p. 247–248 °C; ^{1}H NMR (300 MHz, DMSO): *δ* 8.63 (d, *J* = 6.9 Hz, 1H), 8.44 (s, 1H), 7.98 (d, *J* = 16.2 Hz, 2H), 7.86 (d, *J* = 6.8 Hz, 1H), 7.79 (d, *J* = 8.6 Hz, 2H), 7.60 (d, *J* = 8.6 Hz, 2H), 7.28–7.10 (m, 2H), 6.80 (d, *J* = 8.8 Hz, 4H), 4.66 (t, *J* = 6.5 Hz, 2H), 3.50–3.38 (m, 2H), 3.30–3.22 (m, 6H), 3.04 (s, 6H), 3.02 (s, 6H), 3.09–2.95 (m, 6H), 2.36–2.20 (m, 2H); ^{13}C NMR (75 MHz, DMSO): *δ* 152.3 (C_q), 152.0 (C_q), 151.8 (C_q), 151.7 (C_q), 143.8 (CH), 143.7 (CH), 141.1 (CH), 130.8 (CH), 129.9 (CH), 122.7 (C_q), 122.5 (C_q), 119.4 (CH), 118.6 (CH), 117.6 (CH), 112.0 (CH), 111.8 (CH), 110.5 (CH), 59.8 (CH_2), 52.6 (CH_2), 51.8 (CH_2), 44.7 (CH_2), 39.8 (CH_3), 22.3 (CH_2); MS (ESI$^+$): *m*/*z* = 636.6 [*M* + CF_3COO^-]$^+$, 261.9 [*M*]$^{2+}$; anal. calcd. for $C_{31}H_{45}Br_2N_5$ × 2.5 H_2O (728.6): C 56.05, H 6.92, N 9.61; found: C 56.18, H 6.88, N 9.52.

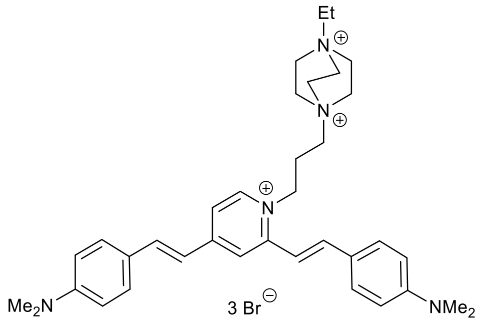

2,4-Bis((*E*)-4-(dimethylamino)styryl)-1-(3-(4-ethyl-1,4-diazoniabicyclo[2.2.2]octyl)-propyl)pyridinium tribromide (5a): Prepared in 13% yield from **I5** and 4-(dimethylamino)benzaldehyde, followed by recrystallization from $CHCl_3$–EtOH. Black solid, m.p. 248–249 °C; ^{1}H NMR (300 MHz, DMSO): *δ* 8.67 (d, *J* = 6.9 Hz, 1H), 8.46 (s, 1H), 8.00 (d, *J* = 16.3 Hz, 2H), 7.87 (d, *J* = 7.1 Hz, 1H), 7.83 (d, *J* = 8.8 Hz, 2H), 7.61 (d, *J* = 8.8 Hz, 2H), 7.29–7.02 (m, 2H), 6.85–6.73 (m, 4H), 4.71 (t, *J* = 6.7 Hz, 2H), 4.05–3.76 (m, 14H), 3.62 (q, *J* = 7.1 Hz, 2H), 3.04 (s, 6H), 3.02 (s, 6H), 2.43–2.29 (m, 2H), 1.28 (t, *J* = 7.1 Hz, 3H); ^{13}C NMR (75 MHz, DMSO): *δ* 152.3 (C_q), 152.0 (C_q), 151.9 (C_q), 151.8 (C_q), 143.8 (CH), 143.7 (CH), 141.1 (CH), 130.9 (CH), 129.9 (CH), 122.7 (C_q), 122.5 (C_q), 119.4 (CH), 118.5 (CH), 117.6 (CH), 112.0 (CH), 111.7 (CH), 110.4 (CH), 60.1 (CH_2), 59.2 (CH_2), 52.2 (CH_2), 50.7 (CH_2), 49.9 (CH_2), 39.7 (2 CH_3), 22.7 (CH_2), 7.5 (CH_3); MS (ESI$^+$): *m*/*z* = 778.6 [*M* + 2 CF_3COO^-]$^+$,

332.9 [M + CF_3COO^-]$^{2+}$, 275.9 [M − H]$^{2+}$, 261.8 [M − Et]$^{2+}$; anal. calcd. for $C_{36}H_{50}Br_3N_5$ × H_2O (810.6): C 53.35, H 6.47, N 8.64; found: C 53.45, H 6.61, N 8.64.

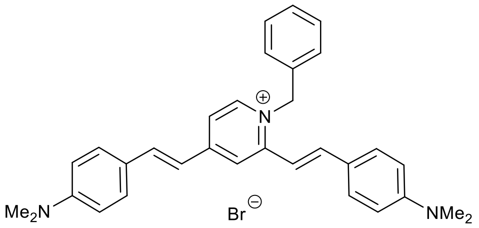

1-Benzyl-2,4-bis((*E*)-4-(dimethylamino)styryl)pyridinium bromide (6a): A mixture of 2,4-bis((*E*)-4-dimethylaminostyryl)pyridine [13] (280 mg, 758 mmol) and benzyl bromide (0.18 mL, 260 mg, 1.52 mmol) in MeCN (7 mL) was heated at reflux for 16 h. After cooling, the precipitated solid was collected, washed with MeCN and recrystallized from $CHCl_3$–EtOH, to give **6a** (230 mg, 56%) as dark-red crystals, m.p. 243–244 °C; ^{1}H NMR (300 MHz, DMSO): δ 8.76 (d, J = 6.7 Hz, 1H), 8.39 (s, 1H), 7.96 (d, J = 16.1 Hz, 1H), 7.88 (d, J = 6.6 Hz, 1H), 7.81 (d, J = 15.6 Hz, 1H), 7.68–7.47 (m, 4H), 7.46–7.25 (m, 5H), 7.18 (d, J = 15.7 Hz, 1H), 7.16 (d, J = 16.0 Hz, 1H), 6.89–6.68 (m, 4H), 5.90 (s, 2H), 3.02 (s, 12H); ^{13}C NMR (75 MHz, DMSO): δ 152.5 (C_q), 152.0 (C_q), 151.9 (C_q), 151.8 (C_q), 144.4 (CH), 142.8 (CH), 141.2 (CH), 135.1 (C_q), 130.3 (CH), 129.9 (CH), 129.1 (CH), 128.4 (CH), 127.1 (CH), 122.8 (C_q), 122.4 (C_q), 119.5 (CH), 118.6 (CH), 117.7 (CH), 112.1 (CH), 111.9 (CH), 111.3 (CH), 58.5 (CH_2), 39.8 (CH_3), 39.7 (CH_3); MS (ESI$^+$): m/z = 460.5 [M]$^+$; anal. calcd. for $C_{32}H_{34}BrN_3$ × 0.5 H_2O (549.5): C 69.94, H 6.42, N 7.65; found: C 69.70, H 6.34, N 7.60.

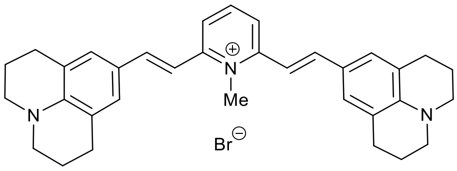

2,6-Bis((*E*)-2-(julolidin-9-yl)vinyl]-1-methylpyridinium bromide (7b): Prepared in 77% yield from **I7** and julolidine-9-carbaldehyde, followed by ion exchange to prepare the bromide salt and recrystallization from MeOH. Crimson-red solid, m.p. > 310 °C; ^{1}H NMR (300 MHz, DMSO): δ 8.11 (t, J = 7.8 Hz, 1H), 7.97 (d, J = 8.0 Hz, 2H), 7.54 (d, J = 15.6 Hz, 2H), 7.24 (s, 4H), 7.15 (d, J = 15.7 Hz, 2H), 4.12 (s, 3H), 3.29–3.16 (m, 8H), 2.71 (br s, 8H), 1.88 (br s, 8H); ^{13}C NMR (75 MHz, DMSO): δ 153.6 (C_q), 144.9 (C_q), 143.0 (CH), 140.8 (CH), 127.9 (CH), 121.5 (C_q), 120.6 (C_q), 120.2 (CH), 111.4 (CH), 49.3 (CH_2), 40.7 (CH_3), 27.1 (CH_2), 21.0 (CH_2); MS (ESI$^+$): m/z = 488.6 [M]$^+$, 244.4 [M + H]$^{2+}$; anal. calcd. for $C_{34}H_{38}BrN_3$ × H_2O (586.6): C 69.61, H 6.87, N 7.16; found: C 69.69, H 6.20, N 6.91.

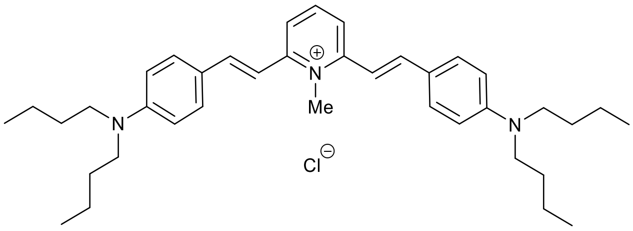

2,6-Bis((*E*)-4-(dibutylamino)styryl)-1-methylpyridinium chloride (7ð): Prepared in 51% yield from **I7** and 4-(dibutylamino)benzaldehyde, followed by ion exchange to prepare the chloride salt and recrystallization from MeCN. Cherry red solid, m.p. 184–185 °C; ^{1}H NMR (300 MHz, DMSO): *δ* 8.17 (t, *J* = 7.9 Hz, 1H), 8.05 (d, *J* = 7.9 Hz, 2H), 7.78–7.55 (m, 6H), 7.25 (d, *J* = 15.7 Hz, 2H), 6.71 (d, *J* = 8.8 Hz, 4H), 4.16 (s, 3H), 3.46–3.30 (m, 8H), 1.61–1.43 (m, 8H), 1.34 (dq, *J* = 14.3, 7.2 Hz, 8H), 0.93 (t, *J* = 7.2 Hz, 12H); ^{13}C NMR (75 MHz, DMSO): *δ* 153.6 (C_q), 149.7 (C_q), 142.7 (CH), 141.2 (CH), 130.6 (CH), 121.8 (C_q), 120.8 (CH), 112.2 (CH), 111.3 (CH), 49.8 (CH_2), 40.9 (CH_3), 29.0 (CH_2), 19.6 (CH_2), 13.9 (CH_3); MS (ESI$^+$): *m*/*z* = 552.7 [*M*]$^+$; anal. calcd. for $C_{38}H_{54}ClN_3$ × 1.5 H_2O (615.3): C 74.17, H 9.34, N 6.83; found: C 74.22, H 9.42, N 6.81.

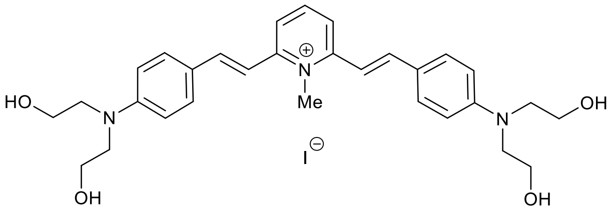

2,6-Bis((*E*)-4-(bis(2-hydroxyethyl)amino)styryl)-1-methylpyridinium iodide (7e): Prepared in 55% yield from **I7** and 4-[bis(2-hydroxyethyl)amino]benzaldehyde, followed by recrystallization from MeOH. Dark-red crystals, m.p. 138–140 °C; ^{1}H NMR (300 MHz, DMSO): *δ* 8.18 (t, *J* = 7.9 Hz, 1H), 8.06 (d, *J* = 7.9 Hz, 2H), 7.75–7.58 (m, 6H), 7.27 (d, *J* = 15.7 Hz, 2H), 6.79 (d, *J* = 8.5 Hz, 4H), 4.80 (br s, 4H, *OH*), 4.18 (s, 3H), 3.65 – 3.43 (m, 16H); ^{13}C NMR (75 MHz, DMSO): *δ* 153.7 (C_q), 150.2 (C_q), 142.7 (CH), 141.4 (CH), 130.5 (CH), 122.2 (C_q), 121.0 (CH), 112.5 (CH), 111.5 (CH), 58.2 (CH_2), 53.2 (CH_2), 41.0 (CH_3); MS (ESI$^+$): *m*/*z* = 504.5 [*M*]$^+$, 252.8 [*M* + H]$^{2+}$, 243.8 [*M* – H_2O + H]$^{2+}$; anal. calcd. for $C_{30}H_{38}IN_3O_4$ × H_2O (649.6): C 55.47, H 6.21, N 6.47; found: C 55.51, H 6.10, N 6.33.

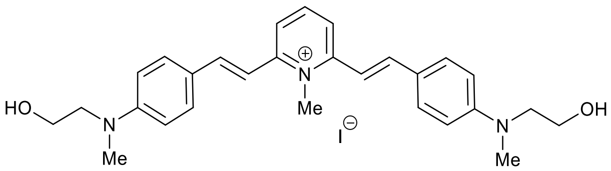

2,6-Bis((*E*)-4-((2-hydroxyethyl)(methyl)amino)styryl)-1-methylpyridinium iodide (7f): Prepared in 39% yield from **I7** and *N*-methyl-*N*-(2-hydroxyethyl)-4-aminobenzaldehyde, followed by recrystallization from MeCN–MeOH. Black solid, m.p. 244–245 °C; ^{1}H NMR (300 MHz, DMSO): *δ* 8.18 (t, *J* = 8.1 Hz, 1H), 8.06 (d, *J* = 8.1 Hz, 2H), 7.73–7.60 (m, 6H), 7.28 (d,

J = 15.7 Hz, 2H), 6.78 (d, *J* = 8.7 Hz, 4H), 4.75 (t, *J* = 5.1 Hz, 2H, *OH*), 4.18 (s, 3H), 3.63–3.54 (m, 4H), 3.54–3.45 (m, 4H), 3.03 (s, 6H); ^{13}C NMR (75 MHz, DMSO): *δ* ^{13}C NMR (75 MHz, DMSO) δ 153.6 (C_q), 151.0 (C_q), 142.8 (CH), 141.3 (CH), 130.4 (CH), 122.3 (C_q), 121.0 (CH), 112.5 (CH), 111.5 (CH), 58.2 (CH_2), 53.9 (CH_2), 41.0 (CH_3), 38.8 (CH_3); MS (ESI^+): *m*/*z* = 444.4 [*M*]$^+$, 222.8 [*M* + H]$^{2+}$, 213.8 [*M* – H_2O + H]$^{2+}$; anal. calcd. for $C_{28}H_{34}IN_3O_2$ (571.5): C 58.85, H 6.00, N 7.35; found: C 58.68, H 5.95, N 7.34.

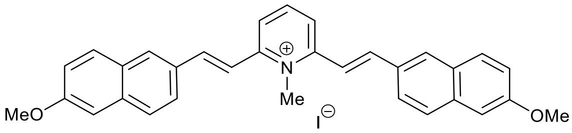

2,6-Bis((*E*)-2-(6-methoxynaphthalen-2-yl)vinyl)-1-methylpyridinium iodide (7i): Prepared in 35% yield from **I7** and 6-methoxy-2-naphthaldehyde, followed by recrystallization from $MeNO_2$. Orange crystalline solid, m.p. 250 °C (decomp.); ^{1}H NMR (300 MHz, DMSO): *δ* 8.44 (t, *J* = 8.0 Hz, 1H), 8.30 (d, *J* = 8.0 Hz, 2H), 8.23 (s, 2H), 8.09 (d, *J* = 8.7 Hz, 2H), 7.96–7.85 (m, 6H), 7.73 (d, *J* = 15.9 Hz, 2H), 7.41 (d, *J* = 2.3 Hz, 2H), 7.24 (dd, *J* = 9.0, 2.5 Hz, 2H), 4.33 (s, 3H), 3.90 (s, 6H); ^{13}C NMR (75 MHz, DMSO): *δ* 158.6 (C_q), 153.3 (C_q), 142.8 (CH), 142.6 (CH), 135.4 (C_q), 130.5 (C_q), 130.2 (CH), 129.9 (CH), 128.2 (C_q), 127.5 (CH), 124.8 (CH), 123.5 (CH), 119.4 (CH), 118.2 (CH), 106.4 (CH), 55.4 (CH_3), 41.7 (CH_3); MS (ESI^+): *m*/*z* = 458.4 [*M*]$^+$; anal. calcd. for $C_{32}H_{28}INO_2$ (585.5): C 65.65, H 4.82, N 2.39; found: C 65.42, H 5.04, N 2.23.

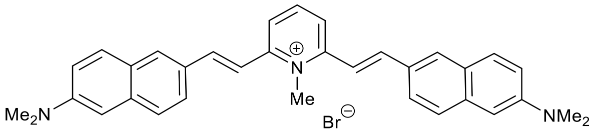

2,6-Bis((*E*)-2-(6-(dimethylamino)naphthalen-2-yl)vinyl)-1-methylpyridinium bromide (7n): Prepared in 54% yield from **I7** and 6-dimethylamino-2-naphthaldehyde, followed by ion exchange to prepare the bromide salt and recrystallization from MeOH. Dark-green crystals, m.p. 278–279 °C; ^{1}H NMR (300 MHz, DMSO): δ 8.35 (t, *J* = 8.0 Hz, 1H), 8.22 (d, *J* = 8.0 Hz, 2H), 8.07 (s, 2H), 7.95 (dd, *J* = 8.8, 1.2 Hz, 2H), 7.90–7.78 (m, 4H), 7.74 (d, *J* = 8.8 Hz, 2H), 7.59 (d, *J* = 15.8 Hz, 2H), 7.26 (dd, *J* = 9.2, 2.4 Hz, 2H), 6.97 (d, *J* = 2.2 Hz, 2H), 4.27 (s, 3H), 3.06 (s, 12H); ^{13}C NMR (75 MHz, DMSO): *δ* 153.4 (C_q), 149.5 (C_q), 142.9 (CH), 142.2 (CH), 135.9 (C_q), 130.3 (CH), 129.6 (CH), 128.4 (C_q), 126.6 (CH), 125.4 (C_q), 124.4 (CH), 122.6 (CH), 116.5 (CH), 116.4 (CH), 105.4 (CH), 41.4 (CH_3), 40.03 (CH_3); MS (ESI^+): *m*/*z* = 484.5 [*M*]$^+$, 242.8 [*M* + H]$^{2+}$; anal. calcd. for $C_{34}H_{34}BrN_3 \times 0.5\ H_2O$ (573.6): C 71.20, H 6.15, N 7.33; found: C 71.18, H 6.21, N 7.08.

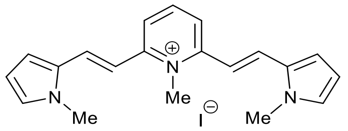

1-Methyl-2,6-bis((*E*)-2-(1-methylpyrrol-2-yl)vinyl)pyridinium iodide (7x): Prepared in 53% yield from **I7** and pyrrole-2-carboxaldehyde, followed by recrystallization from MeOH. Red needles, m.p. 252 °C (decomp.); ^{1}H NMR (300 MHz, DMSO): δ 8.28–8.11 (m, 3H), 7.68 (d, J = 15.5 Hz, 2H), 7.20 (d, J = 15.5 Hz, 2H), 7.12–7.00 (m, 4H), 6.21 (dd, J = 3.7, 2.7 Hz, 2H), 4.15 (s, 3H), 3.80 (s, 6H); ^{13}C NMR (75 MHz, DMSO): δ 153.3 (C_q), 141.2 (CH), 130.4 (CH), 130.3 (C_q), 128.3 (CH), 121.2 (CH), 112.8 (CH), 112.5 (CH), 109.6 (CH), 40.9 (CH_3), 34.0 (CH_3); MS (ESI$^+$): m/z = 304.3 [M]$^+$; anal. calcd. for $C_{20}H_{22}IN_3$ × 0.3 H2O (436.7): C 55.00, H 5.22, N 9.62; found: C 55.00, H 5.17, N 9.82.

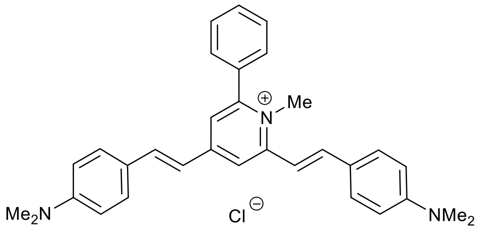

2,4-Bis((*E*)-4-(dimethylamino)styryl)-1-methyl-6-phenylpyridinium chloride (8a): Prepared in 31% yield from **I8** and 4-(dimethylamino)benzaldehyde, followed by ion exchange to prepare the chloride salt and recrystallization from EtOH. Dark-green solid, m.p. 192–193 °C; ^{1}H NMR (300 MHz, DMSO): δ 8.40 (d, J = 1.1 Hz, 1H), 8.02 (d, J = 16.2 Hz, 1H), 7.91 (d, J = 15.7 Hz, 1H), 7.76 (d, J = 1.2 Hz, 1H), 7.73 (d, J = 8.8 Hz, 2H), 7.67 (s, 5H), 7.57 (d, J = 8.8 Hz, 2H), 7.27 (d, J = 15.7 Hz, 1H), 7.16 (d, J = 16.1 Hz, 1H), 6.86–6.71 (m, 4H), 3.87 (s, 3H), 3.03 (s, 6H), 3.01 (s, 6H); ^{13}C NMR (75 MHz, DMSO): δ 153.8 (C_q), 153.6 (C_q), 151.9 (C_q), 151.7 (C_q), 151.3 (C_q), 143.1 (CH), 141.0 (CH), 133.8 (C_q), 130.5 (CH), 130.4 (CH), 129.8 (CH), 129.1 (CH), 129.0 (CH), 122.8 (C_q), 122.6 (C_q), 120.7 (CH), 118.5 (CH), 117.5 (CH), 112.6 (CH), 112.0 (CH), 111.8 (CH), 42.0 (CH_3), 39.7 (CH_3); MS (ESI$^+$): m/z = 460.5 [M]$^+$; anal. calcd. for $C_{32}H_{34}ClN_3$ × H_2O (514.1): C 74.76, H 7.06, N 8.17; found: C 74.46, H 6.91, N 8.10.

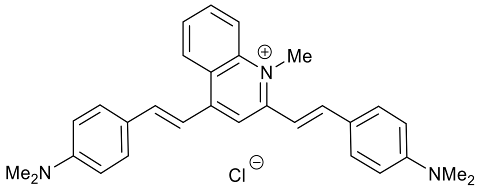

2,4-Bis((*E*)-4-(dimethylamino)styryl)-1-methylquinolinium chloride (9a): Prepared in 43% yield from **I9** and 4-(dimethylamino)benzaldehyde, followed by ion exchange to prepare the chloride salt and recrystallization from AcOEt–MeOH. Shiny dark-green solid, m.p. 223–234 °C; ^{1}H NMR (300 MHz, DMSO): δ 8.80 (d, J = 8.1 Hz, 1H), 8.54 (s, 1H), 8.33 (d, J = 8.9 Hz, 1H), 8.24 (d, J = 15.8 Hz, 1H), 8.18 (d, J = 16.2 Hz, 1H), 8.05 (t, J = 7.7 Hz, 1H), 7.92–7.76 (m, 6H), 7.48 (d, J = 15.6 Hz, 1H), 6.80 (d, J = 8.9 Hz, 4H), 4.33 (s, 3H), 3.05 (s, 6H), 3.04 (s,

6H); ^{13}C NMR (75 MHz, DMSO): *δ* 154.1 (C_q), 152.2 (C_q), 151.7 (C_q), 149.0 (C_q), 146.4 (CH), 142.4 (CH), 139.6 (C_q), 133.7 (CH), 131.2 (CH), 130.6 (CH), 127.2 (CH), 125.9 (CH), 124.5 (C_q), 123.5 (C_q), 122.7 (C_q), 118.9 (CH), 113.7 (CH), 112.9 (CH), 112.8 (CH), 111.8 (CH), 111.7 (CH), 39.7 (CH_3), 39.6 (CH_3), 38.6 (CH_3); MS (ESI$^+$): *m*/*z* = 434.5 [*M*]$^+$; anal. calcd. for $C_{30}H_{32}ClN_3$ × 0.5 H_2O (479.1): C 75.22, H 6.94, N 8.77; found: C 75.00, H 6.89, N 8.75.

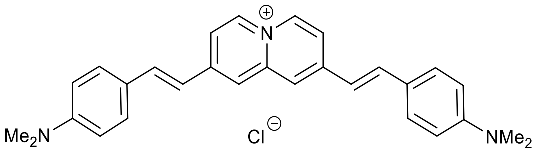

2,8-Bis((*E*)-4-(dimethylamino)styryl)quinolizinium chloride (10a): Prepared in 38% yield through the reaction of **I10** with 4-(dimethylamino)benzaldehyde in MeCN, followed by ion exchange to prepare the chloride salt and recrystallization from AcOEt–MeOH. Dark green solid, m.p. > 310 °C; ^{1}H NMR (300 MHz, DMSO): *δ* 8.94 (d, *J* = 7.1 Hz, 2H), 8.18–7.97 (m, 4H), 7.73 (d, *J* = 16.1 Hz, 2H), 7.55 (d, *J* = 8.7 Hz, 4H), 7.16 (d, *J* = 16.1 Hz, 2H), 6.75 (d, *J* = 8.8 Hz, 4H), 3.00 (s, 12H); ^{13}C NMR (75 MHz, DMSO): *δ* 151.4 (C_q), 145.6(C_q), 142.9(C_q), 138.8 (CH), 135.5 (CH), 129.5 (CH), 122.9 (C_q), 120.7 (CH), 118.2 (CH), 117.9 (CH), 112.0 (CH), 39.7 (CH_3); MS (ESI$^+$): *m*/*z* = 420.4 [*M*]$^+$; anal. calcd. for $C_{29}H_{30}ClN_3$ × H_2O (474.4): C 73.48, H 6.80, N 8.86; found: C 73.38, H 7.13, N 8.63.

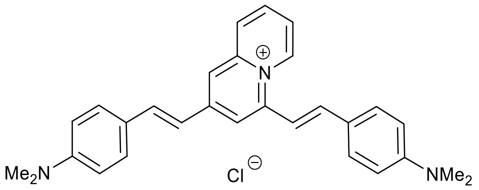

2,4-Bis((*E*)-4-(dimethylamino)styryl)quinolizinium chloride (11a): Prepared in 18% yield through the reaction of **I11** with 4-(dimethylamino)benzaldehyde in MeCN, followed by ion exchange to prepare the chloride salt and recrystallization from EtOH. Black solid, m.p. 208–209 °C; ^{1}H NMR (300 MHz, DMSO): *δ* 9.40 (d, *J* = 7.0 Hz, 1H), 8.49 (s, 1H), 8.31 (s, 2H), 8.24–8.07 (t, 1H), 7.94 (d, *J* = 16.1 Hz, 1H), 7.85–7.50 (m, 7H), 7.21 (d, *J* = 16.2 Hz, 1H), 6.93–6.66 (m, 4H), 3.02 (s, 6H), 3.01 (s, 6H); ^{13}C NMR (75 MHz, DMSO): *δ* 151.4 (2 C_q), 145.5 (C_q), 145.1 (C_q), 143.2 (C_q), 141.4 (CH), 139.2 (CH), 135.0 (CH), 132.4 (CH), 129.9 (CH), 129.4 (CH), 127.4 (CH), 123.0 (C_q), 121.4 (CH), 119.3 (CH), 118.2 (CH), 118.1 (CH), 112.0 (CH), 111.8 (CH), 39.8 (CH_3); MS (ESI$^+$): *m*/*z* = 420.4 [*M*]$^+$; anal. calcd. for $C_{29}H_{30}ClN_3$ × 1.3 H_2O (479.4): C 72.65, H 6.85, N 8.76; found: C 72.69, H 6.86, N 8.81.

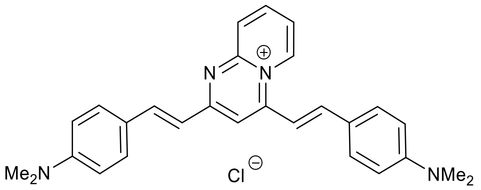

2,4-Bis((*E*)-4-(dimethylamino)styryl)-1-azaquinolizinium chloride (12a): Prepared in 14% yield through the reaction of **I12** with 4-(dimethylamino)benzaldehyde in acetic anhydride, followed by ion exchange to prepare the chloride salt and recrystallization from AcOEt–MeOH. Black solid, m.p. 261–262 °C; ^{1}H NMR (300 MHz, DMSO): *δ* 9.47 (d, *J* = 6.9 Hz, 1H), 8.47 (s, 1H), 8.39–8.29 (m, 1H), 8.26 (d, *J* = 15.8 Hz, 1H), 8.18–8.00 (m, 2H), 7.90–7.56 (m, 6H), 7.19 (d, *J* = 15.8 Hz, 1H), 6.90–6.71 (m, 4H), 3.05 (s, 6H), 3.04 (s, 6H); ^{13}C NMR (75 MHz, DMSO): *δ* 162.1 (C_q), 152.2 (C_q), 152.1 (C_q), 150.5 (C_q), 149.4 (C_q), 144.9 (CH), 144.1 (CH), 139.9 (CH), 132.3 (CH), 131.0 (CH), 130.7 (CH), 127.1 (CH), 122.7 (C_q), 122.4 (C_q), 120.3 (CH), 118.7 (CH), 112.0 (CH), 111.8 (CH), 111.3 (CH), 109.1 (CH), 39.7 (CH_3); MS (ESI$^+$): *m*/*z* = 421.5 [*M*]$^+$; anal. calcd. for $C_{28}H_{29}ClN_4$ × 0.5 H_2O (466.0): C 72.17, H 6.49, N 12.02; found: C 71.76, H 6.57, N 11.83.

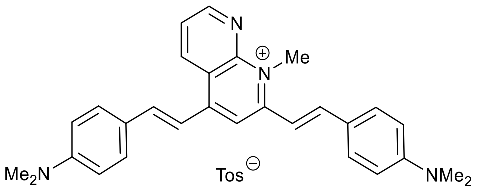

2,4-Bis((*E*)-4-(dimethylamino)styryl)-1-methyl-1,8-naphthyridinium tosylate (13a): Prepared in 39% yield from the reaction of **I13** and 4-(dimethylamino)benzaldehyde in acetic anhydride, followed by recrystallization from EtOH. Shiny black crystals, m.p. 166–168 °C; ^{1}H NMR (300 MHz, DMSO): *δ* 9.24 (d, *J* = 8.0 Hz, 1H), 9.08 (d, *J* = 3.5 Hz, 1H), 8.56 (s, 1H), 8.31 (d, *J* = 15.4 Hz, 1H), 8.15 (d, *J* = 15.6 Hz, 1H), 7.88–7.72 (m, 6H), 7.55–7.38 (m, 3H), 7.10 (d, *J* = 7.9 Hz, 2H), 6.78 (d, *J* = 8.4 Hz, 4H), 4.41 (s, 3H), 3.07 (s, 6H), 3.05 (s, 6H), 2.27 (s, 3H); ^{13}C NMR (75 MHz, DMSO): *δ* 155.4 (C_q), 153.8 (CH), 152.6 (C_q), 151.9 (C_q), 148.9 (C_q), 147.7 (C_q), 147.6 (CH), 145.9 (C_q), 142.7 (CH), 137.5 (C_q), 135.6 (CH), 131.7 (CH), 130.8 (CH), 128.0 (CH), 125.5 (CH), 123.2 (C_q), 122.9 (CH), 122.6 (C_q), 119.7 (C_q), 113.1 (CH), 112.3 (CH), 112.2 (CH), 111.8 (2 CH), 111.7 (CH), 39.7 (CH_3), 34.9 (2 CH_3), 20.8 (CH_3); MS (ESI$^+$): *m*/*z* = 435.5 [*M*]$^+$; anal. calcd. for $C_{36}H_{38}N_4O_3S$ × 0.5 H_2O (615.8): C 70.22, H 6.38, N 9.10; found: C 70.18, H 6.40, N 9.10.

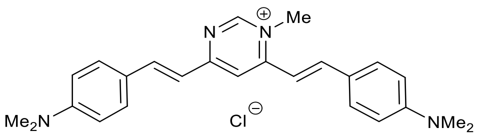

4,6-Bis((*E*)-4-(dimethylamino)styryl)-1-methylpyrimidinium chloride, hydrochloride (14a): Prepared in 33% yield through the reaction of **I14** with 4-(dimethylamino)benzaldehyde,

followed by ion exchange to prepare the chloride salt and recrystallization from MeCN–MeOH. Dark navy blue solid, m.p. 258–259 °C; ^{1}H NMR (300 MHz, DMSO): δ 9.08 (s, 1H), 8.30 (s, 1H), 8.21 (d, J = 15.5 Hz, 1H), 8.11 (d, J = 15.6 Hz, 1H), 7.79 (d, J = 8.8 Hz, 2H), 7.65 (d, J = 8.8 Hz, 2H), 7.10 (d, J = 15.6 Hz, 2H), 6.84 (d, J = 8.1 Hz, 4H), 4.04 (s, 3H), 3.08 (s, 6H), 3.04 (s, 6H); ^{13}C NMR (75 MHz, DMSO) δ 163.6 (C_q), 156.5 (C_q), 153.2 (CH), 152.8 (C_q), 152.0 (C_q), 147.2 (CH), 143.2 (CH), 131.9 (CH), 130.7 (CH), 122.7 (C_q), 122.3 (C_q), 118.1 (CH), 112.4 (CH), 112.1 (CH), 112.0 (CH), 108.7 (CH), 40.6 (CH_3), 39.9 (CH_3); MS (ESI$^+$): m/z = 385.5 [M]$^+$; anal. calcd. for $C_{25}H_{29}ClN_4$ × 2.4 H_2O (464.2): C 64.68, H 7.34, N 12.07; found: C 64.32, H 6.87, N 12.29.

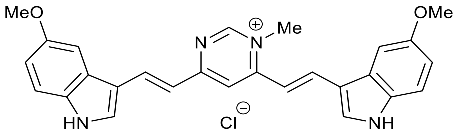

4,6-Bis((*E*)-2-(5-methoxy-1*H*-indol-3-yl)vinyl)-1-methylpyrimidinium chloride (14p): Prepared in 48% yield through the reaction of **I14** with 5-methoxyindole-3-carbaldehyde, followed by ion exchange to prepare the chloride salt and recrystallization from MeCN–MeOH. Dark-green microcrystalline solid, m.p. 295–296 °C; ^{1}H NMR (300 MHz, DMSO): δ 12.33 (s, 1H), 12.04 (s, 1H), 9.05 (s, 1H), 8.58 (d, J = 15.5 Hz, 1H), 8.51–8.39 (m, 2H), 8.30 (d, J = 2.6 Hz, 1H), 8.08 (d, J = 2.6 Hz, 1H), 7.61 (dd, J = 7.7, 1.4 Hz, 2H), 7.53–7.35 (m, 2H), 7.22 (d, J = 15.6 Hz, 1H), 7.06 (d, J = 15.6 Hz, 1H), 6.94 (d, J = 7.5 Hz, 2H), 4.07 (s, 3H), 3.90 (s, 3H), 3.89 (s, 3H); ^{13}C NMR (75 MHz, DMSO): δ 163.9 (C_q), 156.5 (C_q), 155.5 (C_q), 155.3 (C_q), 152.9 (CH), 140.7 (CH), 137.7 (CH), 134.4 (CH), 134.0 (CH), 132.7 (C_q), 132.3 (C_q), 126.2 (C_q), 125.6 (C_q), 116.8 (CH), 114.0 (C_q), 113.6 (CH), 113.5 (C_q), 113.4 (CH), 112.6 (CH), 112.1 (CH), 111.2 (CH), 107.7 (CH), 103.5 (CH), 102.8 (CH), 55.9 (CH_3), 55.6 (CH_3), 40.2 (CH_3); MS (ESI$^+$): m/z = 437.4 [M]$^+$; anal. calcd. for $C_{27}H_{25}ClN_4O_2$ × 0.5 H_2O (482.0): C 67.28, H 5.44, N 11.62; found: C 67.50, H 5.29, N 11.72.

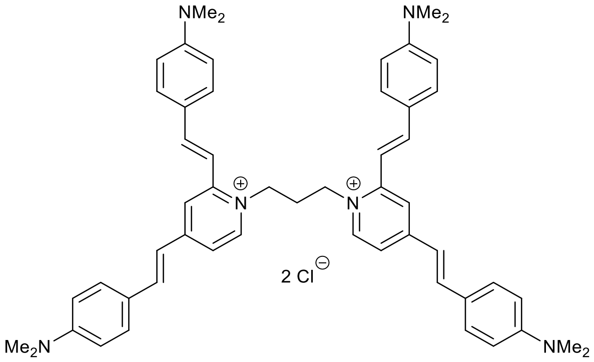

1,1′-(1,3-Propanediyl)-bis(2,4-bis((*E*)-4-(dimethylamino)styryl)pyridinium) dichloride (15a): Prepared in 35% yield through the reaction of **I15** with 4-(dimethylamino)benzaldehyde (6 molar equiv.), followed by ion exchange to prepare the chloride salt and recrystallization from EtOAc–MeOH. Dark-green solid, m.p. 295–296 °C; ^{1}H NMR (300 MHz, DMSO): δ 8.73

(d, J = 6.9 Hz, 2H), 8.37 (s, 2H), 7.93 (d, J = 16.1 Hz, 2H), 7.86 (d, J = 15.6 Hz, 2H), 7.80 (d, J = 6.7 Hz, 2H), 7.72 (d, J = 8.8 Hz, 4H), 7.56 (d, J = 8.8 Hz, 4H), 7.22 (d, J = 15.6 Hz, 2H), 7.12 (d, J = 16.1 Hz, 2H), 6.76 (d, J = 8.9 Hz, 4H), 6.68 (d, J = 8.9 Hz, 4H), 4.89 (t, J = 6.5 Hz, 4H), 3.00 (s, 12H), 2.97 (s, 12H), 2.44–2.34 (m, 2H); ^{13}C NMR (75 MHz, DMSO): δ 152.1 (C_q), 151.8 (C_q), 151.7 (2 C_q), 143.7 (CH), 143.5 (CH), 140.9 (CH), 130.7 (CH), 129.8 (CH), 122.7 (C_q), 122.5 (C_q), 119.4 (CH), 118.6 (CH), 117.7 (CH), 112.0, (CH) 111.7 (CH), 110.4 (CH), 52.7 (CH_2), 39.7 (CH_3), 39.6 (CH_3), 30.0 (CH_2); MS (ESI$^+$): m/z = 894.0 [M + CF_3COO^-]$^+$, 390.5 [M]$^{2+}$; anal. calcd. for $C_{53}H_{60}Cl_2N_6$ × 1.5 H_2O (879.0): C 72.42, H 7.22, N 9.56; found: C 72.43, H 7.26, N 9.51.

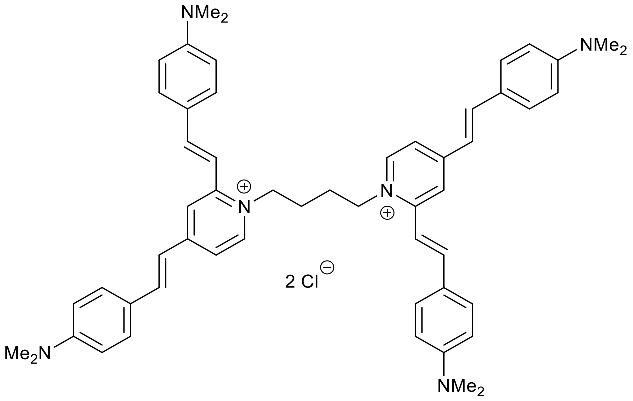

1,1′-(1,4-Butanediyl)-bis(2,4-bis((*E*)-4-(dimethylamino)styryl)pyridinium) dichloride (16a): Prepared in 28% yield through the reaction of **I16** with 4-(dimethylamino)benzaldehyde (6 molar equiv.), followed by ion exchange to prepare the chloride salt and recrystallization from EtOAc–MeOH. Brick-red solid, m.p. > 280 °C; ^{1}H NMR (300 MHz, DMSO): δ 8.55 (d, J = 6.8 Hz, 2H), 8.29 (s, 2H), 7.83 (d, J = 16.2 Hz, 2H), 7.82 (d, J = 15.4 Hz, 2H), 7.70 (d, J = 7.3 Hz, 2H), 7.66 (d, J = 8.8 Hz, 4H), 7.55 (d, J = 8.8 Hz, 4H), 7.15 (d, J = 15.6 Hz, 2H), 7.03 (d, J = 16.0 Hz, 2H), 6.80 (d, J = 8.8 Hz, 4H), 6.71 (d, J = 8.8 Hz, 4H), 4.67 (br s, 4H), 3.03 (s, 12H), 2.97 (s, 12H), 1.87 (br s, 4H); ^{13}C NMR (75 MHz, DMSO): δ 152.1 (C_q), 151.8 (C_q), 151.7 (C_q), 151.5 (C_q), 143.8 (CH), 143.0 (CH), 140.7 (CH), 130.4 (CH), 129.8 (CH), 122.7 (C_q), 122.4 (C_q), 119.4 (CH), 118.3 (CH), 117.5 (CH), 112.0 (CH), 111.7 (CH), 110.8 (CH), 55.0 (CH_2), 39.7 (CH_3), 39.6 (CH_3), 25.6 (CH_2); MS (ESI$^+$): m/z = 908.0 [M + CF_3COO^-]$^+$, 397.6 [M]$^{2+}$; anal. calcd. for $C_{54}H_{62}Cl_2N_6$ × 2 H_2O (902.1): C 71.90, H 7.38, N 9.32; found: C 72.30, H 7.39, N 9.20.

General procedure for the synthesis of mono-styryl dyes by Knoevenagel condensation[2]: A mixture of the heterocyclic salt **I17–I18** (1 mmol), aldehyde (1.5 mmol), and

[2] Except for the dye **19a** whose preparation is detailed below.

piperidine (0.10 mL, 1 mmol) in MeOH (5 mL) was heated under reflux for 2 h. After cooling to room temperature, the precipitated solid was collected by filtration, washed with MeOH (2 × 5 mL), Et_2O (2 × 5 mL), dried and recrystallized from a suitable solvent (as indicated below), to give the analytically pure dye as an iodide salt.

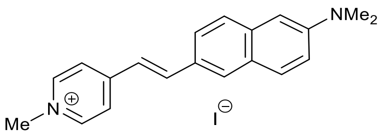

4-((*E*)-2-(6-(dimethylamino)naphthalen-2-yl)vinyl)-1-methylpyridinium iodide (17n): Prepared in 24% yield through the reaction of **I17** with 6-dimethylamino-2-naphthaldehyde, followed by recrystallization from MeOH. Violet needles, m.p. 275–276 °C; ^{1}H NMR (300 MHz, DMSO): δ 8.79 (d, J = 6.4 Hz, 2H), 8.18 (d, J = 6.5 Hz, 2H), 8.09 (d, J = 16.2 Hz, 1H), 8.00 (s, 1H), 7.88–7.66 (m, 3H), 7.47 (d, J = 16.2 Hz, 1H), 7.26 (dd, J = 9.1, 2.1 Hz, 1H), 6.97 (d, J = 1.0 Hz, 1H), 4.23 (s, 3H), 3.06 (s, 6H); ^{13}C NMR (75 MHz, DMSO): δ 152.9 (C_q), 149.6 (C_q), 144.8 (CH), 141.5 (CH), 135.9 (C_q), 130.2 (CH), 129.7 (CH), 128.4 (C_q), 126.8 (CH), 125.5 (C_q), 123.9 (CH), 122.9 (CH), 120.7 (CH), 116.5 (CH), 105.4 (CH), 46.7 (CH3), 40.1 (CH_3); MS (ESI^+): m/z = 289.4 $[M]^+$; anal. calcd. for $C_{20}H_{21}IN_2$ (416.3): C 57.70, H 5.08, N 6.73; found: C 58.02, H 5.13, N 6.43.

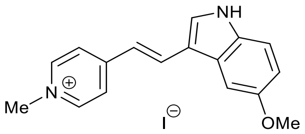

4-((*E*)-2-(5-methoxy-1*H*-indol-3-yl)vinyl)-1-methylpyridinium iodide (17p): Prepared in 58% yield through the reaction of **I17** with 5-methoxyindole-3-carbaldehyde, followed by recrystallization from MeCN–EtOH. Strawberry-colored solid, m.p. 220–222 °C; ^{1}H NMR (300 MHz, DMSO): δ 11.81 (br s, 1H), 8.67 (d, J = 6.8 Hz, 2H), 8.25 (d, J = 16.2 Hz, 1H), 8.11 (d, J = 6.9 Hz, 2H), 7.96 (d, J = 2.7 Hz, 1H), 7.59 (d, J = 2.2 Hz, 1H), 7.40 (d, J = 8.8 Hz, 1H), 7.20 (d, J = 16.2 Hz, 1H), 6.90 (dd, J = 8.8, 2.3 Hz, 1H), 4.17 (s, 3H), 3.87 (s, 3H); ^{13}C NMR (75 MHz, DMSO): δ 155.0 (C_q), 154.2 (C_q), 144.0 (CH), 136.2 (CH), 132.3 (C_q), 132.1 (CH), 125.8 (C_q), 121.5 (CH), 116.2 (CH), 113.4 (C_q), 113.2 (CH), 112.3 (CH), 102.9 (CH), 55.7 (CH_3), 46.2 (CH_3); MS (ESI^+): m/z = 265.3 $[M]^+$; anal. calcd. for $C_{17}H_{17}IN_2O$ × 0.3 H_2O (397.6): C 51.35, H 4.46, N 7.04; found: C 51.30, H 4.58, N 7.06.

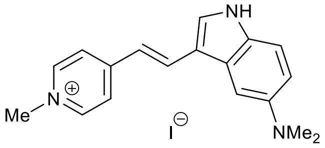

4-((*E*)-2-(5-dimethylamino-1*H*-indol-3-yl)vinyl)-1-methylpyridinium iodide (17s): Prepared in 29% yield through the reaction of **I17** with 5-(dimethylamino)indole-3-carbaldehyde [11], followed by recrystallization from MeCN–EtOH. Black solid, m.p. 268–

269 °C; ^{1}H NMR (300 MHz, DMSO): *δ* 11.70 (s, 1H), 8.63 (d, *J* = 6.7 Hz, 2H), 8.24 (d, *J* = 16.1 Hz, 1H), 8.09 (d, *J* = 6.8 Hz, 2H), 7.88 (s, 1H), 7.34 (t, *J* = 8.9 Hz, 1H), 7.31 (d, *J* = 1.8 Hz, 1H), 7.11 (d, *J* = 16.2 Hz, 1H), 6.87 (dd, *J* = 8.9, 2.1 Hz, 1H), 4.16 (s, 3H), 2.96 (s, 6H); ^{13}C NMR (75 MHz, DMSO): *δ* 154.2 (C_q), 147.0 (C_q), 143.9 (CH), 136.7 (CH), 131.6 (CH), 130.8 (C_q), 126.2 (C_q), 121.3 (CH), 115.5 (CH), 113.1 (C_q), 112.8 (CH), 111.9 (CH), 103.1 (CH), 46.1 (CH_3), 41.8 (CH_3); MS (ESI$^+$): *m*/*z* = 278.3 [*M*]$^+$, 132.2 [*M* – Me + H$^+$]$^{2+}$; anal. calcd. for $C_{18}H_{20}IN_3$ (405.3): C 53.34, H 4.97, N 10.37; found: C 53.29, H 5.09, N 10.34.

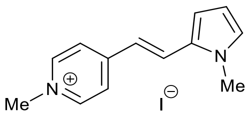

4-((*E*)-2-(1-methylpyrrol-2-yl)vinyl)-1-methylpyridinium iodide (17x): Prepared in 63% yield through the reaction of **I17** with 1-methylpyrrole-2-carboxaldehyde, followed by recrystallization from MeOH. Violet prisms, m.p. 252–254 °C; ^{1}H NMR (300 MHz, DMSO): *δ* 8.69 (d, *J* = 6.7 Hz, 2H), 8.10 (d, *J* = 6.7 Hz, 2H), 7.91 (d, *J* = 15.9 Hz, 1H), 7.08 (d, J = 2.2 Hz, 1H), 7.07 (d, *J* = 15.8 Hz, 1H), 6.88 (d, *J* = 2.6 Hz, 1H), 6.20 (dd, *J* = 3.8, 2.6 Hz, 1H), 4.17 (s, 3H), 3.80 (s, 3H); ^{13}C NMR (75 MHz, DMSO): *δ* 153.2 (C_q), 144.2 (CH), 130.4 (C_q), 129.4 (CH), 128.6 (CH), 122.1 (CH), 117.4 (CH), 112.3 (CH), 109.8 (CH), 46.3 (CH_3), 34.1 (CH_3); MS (ESI$^+$): *m*/*z* = 199.3 [*M*]$^+$; anal. calcd. for $C_{13}H_{15}IN_2$ (326.2): C 47.87, H 4.64, N 8.59; found: C 47.90, H 4.63, N 8.59.

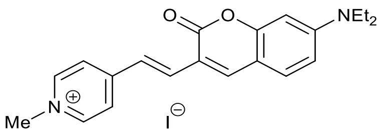

4-((*E*)-2-(7-diethylamino-3-coumarinyl)vinyl)-1-methylpyridinium iodide (17y): Prepared in a 52% yield through the reaction of **I17** with 7-diethylamino-3-formylcoumarin, followed by recrystallization from MeOH. Red solid, m.p. > 280 °C; ^{1}H NMR (300 MHz, DMSO): *δ* 8.78 (d, *J* = 6.4 Hz, 2H), 8.23 (s, 1H), 8.15 (d, *J* = 6.5 Hz, 2H), 7.82 (d, *J* = 16.1 Hz, 1H), 7.67 (d, *J* = 16.0 Hz, 1H), 7.55 (d, *J* = 8.9 Hz, 1H), 6.80 (d, *J* = 8.7 Hz, 1H), 6.60 (s, 1H), 4.22 (s, 3H), 3.49 (q, *J* = 6.4 Hz, 4H), 1.15 (t, *J* = 6.8 Hz, 6H); ^{13}C NMR (75 MHz, DMSO): *δ* 159.6 (C_q), 156.3 (C_q), 152.9 (C_q), 151.9 (C_q), 145.3 (CH), 144.8 (CH), 136.7 (CH), 130.7 (CH), 123.0 (CH), 122.6 (CH), 113.6 (C_q), 110.0 (CH), 108.3 (C_q), 96.2 (CH), 46.7 (CH_3), 44.4 (CH_2), 12.4 (CH_3); MS (ESI$^+$): *m*/*z* = 335.4 [*M*]$^+$; anal. calcd. for $C_{21}H_{23}IN_2O_2$ (462.3): C 54.56, H 5.01, N 6.06; found: C 54.53, H 5.01, N 6.05.

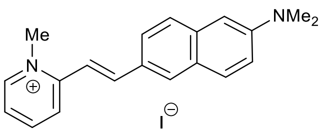

2-((*E*)-2-(6-(dimethylamino)naphthalen-2-yl)vinyl)-1-methylpyridinium iodide (18n): Prepared in 38% yield through the reaction of **I18** with 6-dimethylamino-2-naphthaldehyde,

followed by recrystallization from MeOH. Red crystals, m.p. 233–234 °C; ^{1}H NMR (300 MHz, DMSO): *δ* 8.88 (d, *J* = 6.1 Hz, 1H), 8.54 (d, *J* = 7.5 Hz, 1H), 8.45 (t, *J* = 7.8 Hz, 1H), 8.11 (s, 1H), 8.06 (d, *J* = 15.9 Hz, 1H), 7.95 (dd, *J* = 8.7, 1.3 Hz, 1H), 7.89 – 7.79 (m, 2H), 7.76 (d, *J* = 8.7 Hz, 1H), 7.55 (d, *J* = 15.9 Hz, 1H), 7.29 (dd, *J* = 9.1, 2.3 Hz, 1H), 7.04 (s, 1H), 4.38 (s, 3H), 3.08 (s, 6H); ^{13}C NMR (75 MHz, DMSO): *δ* 152.7 (C_q), 149.3 (C_q), 145.8 (CH), 143.8 (CH), 143.7 (CH), 136.0 (C_q), 130.7 (CH), 129.8 (CH), 128.4 (C_q), 126.7 (CH), 125.6 (C_q), 124.4 (CH), 124.3 (CH), 124.2 (CH), 116.6 (CH), 114.7 (CH), 105.9 (CH), 45.9 (CH_3), 40.2 (CH_3); MS (ESI$^+$): *m*/*z* = 289.4 [*M*]$^+$; anal. calcd. for $C_{20}H_{21}IN_2$ × H_2O (434.3): C 55.31, H 5.34, N 6.45; found: C 54.92, H 5.04, N 6.26.

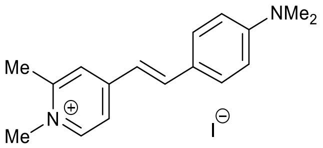

Synthesis of 4-((*E*)-4-(dimethylamino)styryl)-1,2-dimethylpyridinium iodide (19a)

4-((*E*)-4-(dimethylamino)styryl)-2-methylpyridine: A stirred solution of 4-(dimethylamino)-benzaldehyde (1.49 g, 10.0 mmol) and *t*-BuOK (1.23 g, 11.0 mmol) in anhydrous DMF (20 mL) was degassed by repeated vacuum–argon cycles and neat 2,4-lutidine (1.73 mL, 1.61 g, 15.0 mmol) was added via a syringe under argon atmosphere at room temperature. The mixture was stirred at room temperature for 24 h, and a second portion of *t*-BuOK (1.12 g, 10 mmol) was added under argon. After stirring for additional 24 h, the mixture was poured into water (200 mL) and the product was extracted with toluene (3 × 50 mL). The combined organic fractions were washed with water and brine and dried over Na_2SO_4. The solvent was removed under vacuum. Excess 2,4-lutidine was removed by co-evaporation with mesitylene (4 × 10 mL) in vacuo. The crude residue was purified by flash chromatography (SiO_2, eluent: cyclohexane–MTBE, 60:40 to 30:70), to give 4-((*E*)-4-dimethylaminostyryl)-2-methylpyridine (0.82 g, 34%) as a dark-yellow solid, m.p. 156–158 °C; ^{1}H NMR (300 MHz, $CDCl_3$): *δ* 8.40 (d, *J* = 5.2 Hz, 1H), 7.43 (d, *J* = 8.8 Hz, 2H), 7.23 (d, *J* = 16.5 Hz, 1H), 7.19 (s, 1H), 7.15 (d, *J* = 5.3 Hz, 1H), 6.78 (d, *J* = 16.3 Hz, 1H), 6.71 (d, *J* = 8.9 Hz, 2H), 3.00 (s, 6H), 2.55 (s, 3H); ^{13}C NMR (75 MHz, $CDCl_3$) δ 158.6 (C_q), 150.9 (C_q), 149.4 (CH), 146.0 (C_q), 133.1 (CH), 128.4 (CH), 124.6 (C_q), 121.6 (CH), 120.1 (CH), 117.7 (CH), 112.3 (CH), 40.4 (CH_3), 24.6 (CH_3); MS (ESI$^+$): *m*/*z* = 239.2 [*M* + H]$^+$.

4-((*E*)-4-(dimethylamino)styryl)-1,2-dimethylpyridinium iodide (19a): A solution of **4-((*E*)-4-dimethylaminostyryl)-2-methylpyridine** (0.650 g, 2.73 mmol) and iodomethane (0.26 mL, 0.58 g, 4.1 mmol) in anhydrous acetone (27 mL) was stirred at room temperature for 48 h. The precipitate was collected, washed with acetone and dried, to give **19a** (0.816 g, 79%) as a red solid, m.p. 149–150 °C; ^{1}H NMR (300 MHz, DMSO): *δ* 8.68 (d, *J* = 6.7 Hz, 1H), 8.01 (d,

J = 1.6 Hz, 1H), 7.92–7.82 (m, 2H), 7.58 (d, *J* = 8.9 Hz, 2H), 7.10 (d, *J* = 16.1 Hz, 1H), 6.78 (d, *J* = 8.9 Hz, 2H), 4.08 (s, 3H), 3.01 (s, 6H), 2.69 (s, 3H); ^{13}C NMR (75 MHz, DMSO): *δ* 154.1 (C_q), 153.3 (C_q), 151.8 (C_q), 145.0 (CH), 141.4 (CH), 130.0 (CH), 123.4 (CH), 122.5 (C_q), 120.1 (CH), 117.0 (CH), 112.0 (CH), 44.0 (CH_3), 39.7 (CH_3), 19.81 (CH_3); MS (ESI$^+$): *m*/*z* = 253.4 [*M*]$^+$; anal. calcd. for $C_{17}H_{21}IN_2$ × 0.33 H_2O (386.2): C 52.87, H 5.65, N 7.25; found: C 52.93, H 5.63, N 7.28.

Photophysical studies

Buffer and dyes stock solutions. Experiments with nucleic acids were performed in 'K-100' buffer, containing 0.1 M KCl and 0.01 M lithium cacodylate ($LiAsO_2Me_2$) in MilliQ water at pH 7.2 (adjusted with HCl). Absorption studies were performed either in the same buffer or in spectroscopic-grade solvents (methanol and DMSO). Dyes were dissolved in DMSO to obtain 4 mM stock solutions, except for the dyes **1d**, **1ð** and **16a**, which were dissolved at a concentration of 1 mM, and **1m**, which was dissolved at a concentration of 0.5 mM, due to their lower solubility. Stock solutions of dyes were stored at −20 °C. Dye solutions were kept in the dark at all times, to avoid photoinduced degradation.

DNA and RNA stock solutions. Oligonucleotides were purchased from Eurogentec (RP- HPLC purification grade) and used without further purification. Stock solutions were prepared at 100 µM (except for *46AG*: 50 µM) in K-100 buffer and stored at 4 °C. Working solutions (*c* = 5.6 µM, except for *46AG*: *c* = 2.8 µM) were prepared by dilution of stock solutions in the same buffer. Heteroduplexes (*ds 3+4*, *ds 5+8*, and *ds 6+7*, Table S2) were prepared by mixing equal volumes of the corresponding single strands. The final concentration of heteroduplexes was 2.8 µM, to account for the doubled number of nucleotides. The working solutions were subsequently annealed (5 min at 95 °C) and then allowed to cool down to room temperature overnight. Annealed solutions were stored at 4 °C. Calf thymus DNA (*ct DNA*, Invitrogen, 10 mg mL^{-1}) and RNA from calf liver (*cl RNA*, Sigma-Aldrich, Type IV) were diluted with K- 100 buffer to *c* ≈ 3 mM in base pairs, as calculated by the absorption measurement at 260 nm (*ct DNA*: $\varepsilon_{nucleotide}$ = 6650 M^{-1} cm^{-1}, *cl RNA*: $\varepsilon_{nucleotide}$ = 8500 M^{-1} cm^{-1}), and then further diluted so as to obtain working solutions with a nucleotide concentration comparable to the oligonucleotide samples (i.e., 110 µM, considering 22 as the average length of oligonucleotides).

Absorption studies. Absorption spectra of the dyes in K-100 buffer, MeOH and (for some dyes) DMSO were recorded at 20 °C and dye concentration of 10 µM in quartz cells with a path length of 1 cm, using a double-beam spectrophotometer (Hitachi U2900) operating at a

spectral bandpass of 1.5 nm. All samples were checked for the absence of visible precipitates at the moment of the measurements.

Fixed-wavelength fluorescence measurements. All fluorescence analyses were performed with a microplate reader (BMG FluoStar Omega), using a 96-well quartz plate with a transparent bottom (Hellma). Samples were prepared by mixing working solutions of DNA samples (5.6 µM or equivalent in K-100 buffer, 90 µL) or the buffer alone with working solutions of dyes in K-100 buffer (25 µM in K-100 buffer containing 2.5% v/v DMSO, 10 µL). The final concentrations were 2.5 µM for the dye and 5 µM (or equivalent) for DNA in a total volume of 100 µL per well. The plates were stirred for 3 min at 300 rpm and then left to equilibrate for 1 h at room temperature in the dark. Fluorescence emission was recorded by using a microplate reader, exciting each dye at the appropriate wavelength with the aid of appropriate filters (Table S1). The instrument gain was set for each channel and kept constant throughout all the analyses.

Multivariate analysis. The light-up data matrix reported in Table S1 was normalized to a scale of 0 to 1; normalization was always performed for each nucleic acid sequence separately. Multivariate analysis (PCA) was performed with Origin Pro 2018b (OriginLab, Northampton, MA). The PCA data are presented as score plots of PC1 versus PC2.

Fluorescence quantum yield and brightness measurements. Dyes **1p**, **1u**, **17a** and **18a** were diluted in K-100 buffer at varying concentration (1.2–3 µM) alone or in the presence of G4 structures (*c-myc* or *22AG*, 6 µM). Absorption and emission spectra (λ_{ex} = 500 nm, slits width 5 nm, PMT voltage 550 V) of the resulting solutions were measured on a UV Cary-300 spectrophotometer and on a Cary Eclipse fluorimeter, respectively, using a transparent and asymmetric quartz cuvette (1 × 0.4 cm pathlength). Fluorescence emission spectra were integrated between 510 and 800 nm and the obtained values were plotted as a function of the absorbance at 500 nm for each dye. The slope of the resulting plots was used for the quantum yield (Φ) calculations, using the data recorded for Rhodamine 6G at identical settings as a reference (Φ = 0.95 in EtOH [14]):

$$\Phi_{sample} = \Phi_{ref} \times \frac{S_{sample}}{S_{ref}} \times \frac{n^2_{sample}}{n^2_{ref}},$$

where Φ is fluorescence quantum yield, *S* is the slope obtained from the *Area* vs *absorbance* plots and *n* is the refractive index of the solvents; subscripts *ref* and *sample* denote rhodamine 6G and dye or dye-G4 complex, respectively.

Molar absorptivity coefficients for the complexes at the absorption maximum were calculated from the absorbance and concentration data obtained for each dye–G4 complex. Brightness

(*B*) of each complex and of the dyes alone was subsequently calculated through the following formula:

$$B_{sample} = \varepsilon_{max\ sample} \times \Phi_{sample}$$

where Φ is fluorescence quantum yield and ε_{max} is the molar absorptivity coefficient in the absorption maximum. As above, the subscript *sample* denotes the dye or dye-G4 complex.

Table S1: Excitation / emission wavelengths of optical filters (passband: 10 nm) and relative emission intensity enhancement (I/I_0) of styryl dyes and Thioflavin T (**ThT**) (c = 2.5 µM in K-100 buffer) in the presence of 2 molar equiv. of G4-DNA (except for 46AG: 1 molar equiv.), G4-RNA, or ct DNA and cl RNA used at equivalent nucleotide concentration.

**Dye**	**ex / em filters (nm)**	**G4 DNA**										**G4 RNA**		**non-G4**	
		c-kit2	*25CEB*	*c-kit87*	*c-myc*	*c-src1*	*c-myb*	*22AG*	*46AG*	*HRAS*	*TBA*	*TERRA*	*NRAS*	*ct DNA*	*cl RNA*
**1a**	544 / 620	30	22	34	40	10	6	82	99	36	4	170	15	3	4
**1b**	620 / 670	13	14	8	38	5	6	11	28	18	3	111	11	3	2
**1c**	485 / 544	27	26	27	26	12	4	34	97	63	7	59	12	7	6
**1d**	544 / 620	39	24	50	46	7	6	68	346	35	5	120	21	2	3
**1ð**	620 /670	3	3	4	4	2	3	2	2	2	2	46	2	2	2
**1e**	544 / 620	49	41	45	59	15	13	62	112	41	10	114	20	14	7
**1f**	590 / 670	85	72	109	112	32	14	104	113	118	22	147	60	11	7
**1g**	485 / 544	39	19	57	42	8	8	19	73	23	3	45	32	3	3
**1h**	485 / 544	46	17	47	28	18	12	20	59	22	5	144	33	4	7
**1i**	485 / 544	7	6	19	10	3	4	13	30	5	2	39	14	6	1
**1j**	544 / 620	1	1	1	1	1	1	1	1	1	1	1	1	1	1
**1k**	584 / 670	3	4	4	3	2	2	4	7	4	2	5	3	1	1
**1l**	544 / 620	2	2	2	1	1	1	2	2	2	1	3	2	1	2
**1m**	520 / 620	3	4	4	4	3	3	4	4	3	3	5	3	2	3
**1o**	544 / 584	85	49	81	76	51	18	52	115	119	21	353	69	7	16
**1p**	544 / 584	185	141	269	163	75	33	553	352	367	45	268	176	4	7
**1q**	544 / 590	93	29	67	67	13	10	39	52	28	3	355	61	1	11
**1r**	485 / 544	88	27	35	204	8	8	39	98	177	28	320	55	2	13
**1s**	485 / 544	31	54	23	238	8	11	17	50	46	5	16	26	48	9
**1t**	485 / 520	8	18	25	11	6	5	54	80	25	5	40	5	5	2
**1u**	544 / 584	193	27	34	252	11	8	45	41	205	99	383	128	1	33
**1v**	485 / 520	215	168	149	344	49	82	202	209	232	97	497	153	9	35
**1w**	485 /544	6	3	9	4	2	2	3	6	2	2	7	6	3	2
**1x**	544 / 620	346	115	393	282	111	56	148	485	341	26	689	200	36	63
**1y**	544 / 620	13	26	13	38	6	5	23	35	27	2	50	15	2	1
**1z**	544 / 620	1	1	1	1	1	1	1	1	1	1	1	1	1	1
**1þ**	544 / 620	1	1	1	1	1	1	1	1	1	1	5	1	1	1
**2a**	544 / 620	22	20	24	39	9	9	61	76	69	4	122	16	6	3
**2i**	485 / 544	17	13	21	19	9	7	20	49	11	7	19	21	12	6
**2n**	584 / 670	3	3	3	3	2	1	7	7	7	1	21	5	1	1
**3a**	544 / 620	16	13	16	27	7	5	38	41	27	3	103	11	4	3
**4a**	584 / 670	45	39	44	52	19	7	124	112	147	9	267	39	10	8
**5a**	590 / 670	102	110	118	148	57	20	326	215	190	39	311	102	25	11
**6a**	544 / 620	8	9	9	12	3	3	32	30	8	2	20	4	2	2
**7a**	544 / 620	19	12	22	20	9	9	14	28	19	3	168	9	12	4
**7b**	584 / 670	5	4	4	4	2	1	3	5	3	2	22	2	2	2
**7e**	544 / 620	62	76	74	105	20	11	51	118	68	10	69	45	35	3
**7f**	544 / 620	30	35	39	46	18	8	25	55	62	4	52	28	22	2
**7i**	485 / 544	3	4	3	5	4	1	3	5	4	2	34	2	2	2
**7n**	584 / 670	2	2	2	3	2	3	2	3	3	2	4	3	2	2
**7x**	544 / 590	216	86	186	204	33	28	90	181	100	6	195	113	3	9
**7ð**	485 /544	1	1	1	1	1	1	1	1	1	1	1	1	1	1
**8a**	584 / 670	29	25	35	28	7	6	50	66	51	4	97	12	7	3
**9a**	544 / 620	5	4	5	2	2	11	11	20	12	2	6	3	3	2
**10a**	544 / 620	5	4	7	5	3	2	9	12	2	0	8	1	2	0
**11a**	544 / 620	10	23	32	48	15	7	51	96	45	5	154	18	5	6
**12a**	620 / 670	6	9	5	12	5	3	15	43	17	2	56	6	2	3
**13a**	620 / 720	7	10	4	2	7	2	4	19	14	2	20	5	2	2
**14a**	620 /670	12	11	18	17	4	4	89	62	21	3	54	6	2	2
**14p**	520 / 620	80	57	83	111	23	22	132	121	139	32	178	43	3	12
**15a**	584 / 670	11	13	20	10	6	5	19	55	3	4	24	8	2	2
**16a**	584 /670	25	28	41	16	12	60	42	68	32	6	117	33	6	5
**17a**	544 / 620	161	83	244	338	44	15	84	184	83	5	169	92	5	8
**17n**	520 / 670	19	13	22	14	5	4	24	38	16	2	16	8	3	2
**17p**	485 / 544	101	34	81	94	14	14	51	166	78	10	78	100	12	16
**17s**	485 /544	21	8	11	21	3	4	7	17	8	2	21	32	3	7
**17x**	485 / 544	6	2	7	9	2	2	2	6	2	1	6	11	1	1
**17y**	544 / 584	7	5	7	13	2	3	7	11	9	3	10	6	6	2
**18a**	544 / 620	215	61	265	302	70	1	60	166	59	3	10	13	2	11
**18n**	544 / 620	11	15	35	29	8	8	31	102	35	2	62	20	5	3
**19a**	544 / 620	167	118	239	185	50	18	90	172	53	4	144	61	2	8
**ThT**	460 / 544	76	38	102	111	21	16	168	176	102	9	94	203	2	10

Table S2: DNA sequences used in the double-dye analysis.

Acronym	Sequence (5′ → 3′)	Conformation	PDB	*c* (μM)[a]
22AG	AGGGTTAGGGTTAGGGTTAGGG	Hybrid G4	-	5
UpsB-Q3	CAGGGTTAAGGGTATACATTTAGGGGT TAGGGTT	Hybrid G4	-	5
26TTA	TTAGGGTTAGGGTTAGGGTTAGGGTT	Hybrid G4	2JPZ	5
25TAG	TAGGGTTAGGGTTAGGGTTAGGGTT	Hybrid G4	2JSL	5
23TAG	TAGGGTTAGGGTTAGGGTTAGGG	Hybrid G4	2JSK	5
24TTA	TTAGGGTTAGGGTTAGGGTTAGGG	Hybrid G4	2JSL	5
46AG	A(GGGTTA)$_7$GGG	Hybrid G4	-	2.5
Bcl2Mid	GGGCGCGGGAGGAATTGGGCGGG	Hybrid G4	2F8U	5
26CEB	AAGGGTGGGTGTAAGTGTGGGTGGGT	Parallel G4	2LPW	5
c-kit2-T12T21	CGGGCGGGCGCTAGGGAGGGT	Parallel G4	2KYP	5
KRAS-22RT	AGGGCGGTGTGGGAATAGGGAA	Parallel G4	5I2V	5
c-myc	TGAGGGTGGGTAGGGTGGGTAA	Parallel G4	1XAV	5
VEGF	CGGGGCGGGCCTTGGGCGGGGT	Parallel G4	2M27	5
T95-2T	TTGGGTGGGTGGGTGGGT	Parallel G4	2LK7	5
TBA	GGTTGGTGTGGTTGG	Anti-parallel G4	148D	5
HIV-PRO-1	TGGCCTGGGCGGGACTGGG	Anti-parallel G4	-	5
Bm-U16	TAGGTTAGGTTAGGTUAGG	Anti-parallel G4	-	5
Bom17	GGTTAGGTTAGGTTAGG	Anti-parallel G4	-	5
G4CT	GGGGCTGGGGCTGGGGCTGGGG	Anti-parallel G4	-	5
ct DNA	Calf thymus DNA, highly polymerized	Duplex	-	110[b]
ds26	CAATCGGATCGAATTCGATCCGATTG	Duplex (self-complementary)	-	5
ds-lac	GAATTGTGAGCGCTCACAATTC	Duplex (self-complementary)	-	5
ds 5 + 8	GGAGAGAGAGTGTGTGTGTGGG + CCCACACACACACTCTCTCTCC	Heteroduplex	-	2.5[c]

ds 3 + 4	GTCGCCGGGCCAGTCGTCCATAC + GTATGGACGACTGGCCCGGCGAC	Heteroduplex	-	2.5[c]
ds 6 + 7	GACGTGTCGAAAGAGCTCCGATTA + TAATCGGAGCTCTTTCGACACGTC	Heteroduplex	-	2.5[c]
ss1	CACTAAACCTAACACTAACCAT	Single strand	-	5
ss2	ATGCCCTACGCGTCTTCTACTT	Single strand	-	5
ss3	GTCGCCGGGCCAGTCGTCCATAC	Single strand	-	5
ss4	GTATGGACGACTGGCCCGGCGAC	Single strand	-	5
ss5	CCCACACACACACTCTCTCTCC	Single strand	-	5
ss6	GACGTGTCGAAAGAGCTCCGATTA	Single strand	-	5
ss7	TAATCGGAGCTCTTTCGACACGTC	Single strand	-	5
dT_{26}	TTTTTTTTTTTTTTTTTTTTTTTTTT	Single strand	-	5

[a] Final concentration used in the double dye assay, expressed in oligonucleotide strands unless stated otherwise.
[b] Nucleotide concentration. [c] Concentration of duplexes.

REFERENCES

1. Matsui, M.; Kawamura, S.; Shibata, K.; Muramatsu, H. *Bull. Chem. Soc. Jpn.* **1992**, *65*, 71–74.
2. Xie, X.; Choi, B.; Largy, E.; Guillot, R.; Granzhan, A.; Teulade-Fichou, M.-P. *Chem. Eur. J.* **2013**, *19*, 1214–1226.
3. Sato, K.; Okazaki, S.; Yamagishi, T.; Arai, S. *J. Heterocycl. Chem.* **2004**, *41*, 443–447.
4. Bridger, G. J.; Skerlj, R. T.; Padmanabhan, S.; Martellucci, S. A.; Henson, G. W.; Abrams, M. J.; Joao, H. C.; Witvrouw, M.; De Vreese, K.; Pauwels, R.; De Clercq, E. *J. Med. Chem.* **1996**, *39*, 109–119.
5. Campbell, K. N.; Schaffner, I. J. *J. Am. Chem. Soc.* **1945**, *67*, 86–89.
6. Nawrat, C. C.; Lewis, W.; Moody, C. J. *J. Org. Chem.* **2011**, *76*, 7872–7881.
7. Khmaruk, A. M.; Volovenko, Y. M.; Chuiguk, V. A. *Ukr. Khim. Zh.* **1972**, *38*, 262–264.
8. Nam, T.; Wijtmans, M.; Pratt, D. A.; Porter, N. A. *Synthesis* **2005**, 1397–1404.
9. Hamada, Y.; Takeuchi, I.; Sato, M. *Yakugaku Zasshi* **1974**, *94*, 1328–1334.
10. Xie, X.; Renvoisé, A.; Granzhan, A.; Teulade-Fichou, M.-P. *New J. Chem.* **2015**, *39*, 5931–5935.
11. DeGraw, J. I. *Can. J. Chem.* **1966**, *44*, 387–393.
12. Klymchenko, A. S.; Pivovarenko, V. G.; Ozturk, T.; Demchenko, A. P. *New J. Chem.* **2003**, *27*, 1336–1343.
13. Wang, H.; Helgeson, R.; Ma, B.; Wudl, F. *J. Org. Chem.* **2000**, *65*, 5862–5867.
14. Kubin, R. F.; Fletcher, A. N. *J. Lumin.* **1982**, *27*, 455–462.
